# Supplementary material for: Cross-sectional study of association between socioeconomic indicators and chronic kidney disease in rural–urban Ghana: the RODAM study
Source: BMJ Open. 2019 May 24;9(5):e022610. doi: 10.1136/bmjopen-2018-022610 (PMC6537994; doi:10.1136/bmjopen-2018-022610)
Supplement: Author's manuscript [file bmjopen-2018-022610.draft_revisions.pdf]

BMJ Open is committed to open peer review. As part of this commitment we make the peer review history of every article we publish publicly available.

When an article is published we post the peer reviewers' comments and the authors' responses online. We also post the versions of the paper that were used during peer review. These are the versions that the peer review comments apply to.

The versions of the paper that follow are the versions that were submitted during the peer review process. They are not the versions of record or the final published versions. They should not be cited or distributed as the published version of this manuscript.

BMJ Open is an open access journal and the full, final, typeset and author-corrected version of record of the manuscript is available on our site with no access controls, subscription charges or pay-per-view fees (http://bmjopen.bmj.com).

If you have any questions on BMJ Open's open peer review process please email info.bmjopen@bmj.com

BMJ Open

ASSOCIATION BETWEEN SOCIOECONOMIC INDICATORS AND CHRONIC KIDNEY DISEASE IN RURAL-URBAN GHANA- THE RODAM STUDY

Journal:	*BMJ Open*
Manuscript ID	bmjopen-2018-022610
Article Type:	Research
Date Submitted by the Author:	01-Mar-2018

Complete List of Authors:	Adjei Nana, David ; University of Ghana, Department of Medical Laboratory Sciences; University of Amsterdam, Department of Public Health<br>Stronks, Karien; Academic Medical Center , Department of Public Health<br>Adu, Dwomoa; Korle-bu Teaching Hospital, Department of Medicine<br>Beune, Erik; AMC<br>Meeks, Karlijn; AMC, Public Health<br>Smeeth, Liam; London School of Hygiene and Tropical Medicine,<br>Addo, Juliet; London School of Hygiene and Tropical Medicine, Non Communicable Disease Epidemiology<br>Owusu-Dabo, Ellis; Kwame Nkrumah University of Science and Technology, Kumasi Centre for Collaborative Research in Tropical Medicine<br>Klipstein-Grobusch, Kerstin; 1 Julius Global Health, Julius Center for Health Sciences and Primary Care, University Medical Centre Utrecht, Utrecht, The Netherlands<br>Mockenhaupt, Frank; Charité – University Medicine, Berlin, Institute of Tropical Medicine and International Health<br>Danquah, Ina; German Institute of Human Nutrition, Molecular Epidemiology; Charite Universitatsmedizin Berlin, Institute for Social Medicine, Epidemiology and Health Economy<br>Spranger, Joachim; Department of Endocrinology and Metabolism, 1. Charité-University Medicine Berlin, Berlin, Germany.<br>Bahendeka, Silver; 1. MKPGMS - Uganda Martyrs University<br>De-Graft Aikins, Ama; University of Ghana, Regional Institute for Population Studies<br>Agyemang, Charles; Academic Medical centre, University of Amsterdam, Department of Public Health

Keywords:	Chronic Kidney Disease, Socioeconomic status, Health inequalities, RODAM study, rural, urban

SCHOLARONE™
Manuscripts

ASSOCIATION BETWEEN SOCIOECONOMIC INDICATORS AND CHRONIC KIDNEY DISEASE IN RURAL-URBAN GHANA-THE RODAM STUDY

David N. Adjei, MSc[1,2]; Karien Stronks, MSc, PhD[1]; Dwomoa Adu, MD[3]; Erik Beune, MSc, PhD [1]; Karlijn Meeks, MSc [1]; Liam Smeeth, MD, PhD[4] ; Juliet, Addo, MD, PhD [4]; Ellis Owuso-Dabo, MSc, PhD [5], Kerstin Klipstein-Grobusch, MSc, PhD [6,7]; Frank P. Mockenhaupt, MD, PhD [8] ; Ina, Danquah, MSc, PhD [9] ; Joachim, Spranger, MD, PhD [10,11,12]; Silver Bahendeka, MD, PhD[13]; Ama de-Graft, Aikins, MSc, PhD[14]; Charles Agyemang, MPH, PHD[1]

1. Department of Public Health, Academic Medical Center, University of Amsterdam, Amsterdam Public Health Research Institute, Amsterdam, The Netherlands.
2. Department of Medical Laboratory Sciences, School of Biomedical and Allied Health Sciences, College of Health Sciences, University of Ghana, Accra, Ghana.
3. Department of Medicine, School of Medicine and Dentistry, University of Ghana and Korle-Bu Teaching Hospital, Accra, Ghana.
4. Department of Non-communicable Disease Epidemiology, London School of Hygiene and Tropical Medicine, London, United Kingdom.
5. Kumasi Centre for collaborative Research, KNUST, Kumasi, Ghana
6. Julius Global Health, Julius Center for Health Sciences and Primary Care, University Medical Centre, Utrecht University, The Netherlands
7. Division of Epidemiology & Biostatistics, School of Public Health, Faculty of Healths, University of the Witwatersrand, Johannesburg, South Africa
8. Institute of Tropical Medicine and International Health, Charité – University Medicine Berlin, Augustenburger Platz 1, 13353 Berlin, Germany.
9. Department of Molecular Epidemiology, German Institute of Human Nutrition Potsdam-Rehbrücke, Nuthetal, Germany.
10. Department of Endocrinology and Metabolism, Charité-University Medicine Berlin, Berlin, Germany.
11. German Centre for Cardiovascular Research (DZHK), Berlin, Germany.
12. Center for Cardiovascular Research (CCR), Charité-University Medicine Berlin, Berlin, Germany.
13. MKPGMS - Uganda Martyrs University, Kampala, Uganda.
14. Regional Institute for Population Studies, University of Ghana, Legon, Ghana.

Address correspondence to David Nana Adjei, MSc, Department of Public Health, Academic Medical Centre, University of Amsterdam, Meibergdreef 9, 1105 AZ Amsterdam, the Netherlands, School Biomedical and Allied Health Sciences, Medical Laboratory Sciences, University of Ghana, E-mail: dna@chs.edu.gh, d.n.adjei@amc.uva.nl, Tel: +233236717850

For peer review only

Abstract

Objectives Studies from high income countries suggest higher prevalence of Chronic Kidney Disease (CKD) among individuals in low socio economic groups. However, some studies from low and middle-income countries (LMICs) show the reverse pattern among those in high socioeconomic groups. It is unknown which pattern applies to individuals living in rural and urban Ghana. We therefore assessed the association between Socio-Economic Status (SES) indicators and CKD in rural and urban Ghana and to what extent the higher SES of people in urban areas of Ghana could account for differences in CKD between rural and urban populations.

Setting: We used baseline data from multi-centre Research on Obesity and Diabetes among African Migrants (RODAM) study.

Participants: A sample of 2492 adults (Rural Ghana, 1043, Urban Ghana, 1,449 aged 25 to 70 years living in Ghana.

Outcomes & Measurements Three CKD outcomes were considered using the 2012 KDIGO (Kidney Disease: Improving Global Outcomes) severity of CKD classification: albuminuria (albumin-creatinine ratio ≥ 3 mg/mmol (category ≥ A2)); reduced glomerular filtration rate (eGFR < 60 mL/min/1.73 m2 (category ≥ G3)) and high to very high CKD risk based on the combination of these two.

Results All three SES indicators were not associated with CKD in both rural and urban Ghana after age and sex adjustment except for rural Ghana where high wealth index was significantly associated with higher odds of reduced eGFR (AOR, 2.38; 95% C.I. 1.03-5.47). The higher rate of CKD observed in urban Ghana was not explained by the higher SES of that population.

Conclusion SES indicators were not associated with prevalence of CKD except for wealth index and reduced eGFR in rural Ghana. Consequently, the higher SES did not account for the increased rate of CKD among urban dwellers suggesting the need to identify other factors that may be driving this.

Index Words: Chronic kidney disease; socioeconomic status; health inequalities; risk factor; ethnic minority groups; migrants; RODAM study, Ghana

For peer review only

Strengths and limitation of the study

- The use of well-standardized study protocols across rural and urban Ghana eliminated intra protocol variability.

- Our study is also the first in Africa to use all three categories of CKD definition (albuminuria, reduced eGFR and CKD risk) by KDIGO 2012 in assessing association of SES with CKD in rural and urban setting, this provides a more detailed information on CKD outcomes.

- The limitation of intra laboratory variability in earlier studies was eliminated using the same standard operating procedures in the same laboratory for running all samples for both rural and urban Ghana.

- The use of three constructs of SES (educational level, occupational level and wealth index) in this study also provides a much better holistic approach to assessing SES association with CKD. Also, the distribution of SES in our study reflect on the national data allowing for generalization of our findings.

- Our study was limited by the use of cross sectional design which prevented us from determining causality between predictors and CKD progression.

For peer review only

Introduction

In general, individuals in lower socio-economic status (SES) groups have been shown to suffer more frequently from Chronic Kidney Disease (CKD), often progressing to End Stage Renal Disease (ESRD), and associated with inadequate dialysis treatment, reduced access to kidney transplantation and poor health outcomes [1]. Recent studies have consistently found low SES to be associated with higher risk of CKD among people of African origin [2-5].

However, in some settings the well-known inverse association between SES and CKD seems to be absent, or even reversed. For example, Bryne et al. did not find an association between SES and End Stage Renal Disease [6]. Invariably, others studies have consistently found a positive association between SES and CKD [7 8]. Specifically, as SES improved, unhealthful lifestyle (unhealthy diet, physical inactivity, smoking and alcohol consumption) increased in China while that of the United States decreased with improved SES [9]. People with higher incomes, in these contexts, can afford a western lifestyle, which is more readily available in the urban areas than in the rural areas. There is therefore an interaction between individual SES and environmental factors, such as food and sedentary life style in such populations [10-12]. Consequently, in those settings, people with a higher SES might have higher CKD risk.

In urban areas, the population in general has a higher SES than in rural areas [13]. For example, individuals with higher educational level migrate from rural areas to find higher occupations matching their higher education to improve on their wealth. If indeed a positive association between SES and CKD is observed in LMICs, this might underlie the well-known health differences between urban and rural areas, with urban areas having an increased risk of CKD [14]. So far, it is unknown whether the reversed SES gradient (higher risk in high SES group) might explain the higher burden of CKD in urban areas as compared to rural areas in Africa.

In view of this, we assessed the association of SES with CKD in rural and urban Ghana and studied what extent the higher SES of people in urban areas could account for differences in CKD between rural and urban populations.

For peer review only

Methods

Study population and study design

In the present analyses, data used were from the RODAM (**R**esearch on **O**besity & **D**iabetes among **A**frican **M**igrants) study, a multi-centre cross-sectional study, were used. The rationale, conceptual framework, design and methodology of the RODAM study have been described in detail elsewhere [15 16]. As the Healthy Life in an Urban Setting (HELIUS) study conducted among Ghanaian migrants living in Amsterdam did not find any associations between SES and CKD [17] the current study focused on rural and urban Ghana (Ashanti region of Ghana). The RODAM study was conducted from 2012 to 2015 and it comprised of individuals aged 25-70 years living in rural and urban Ghana and Ghanaian migrants in Europe. All participants below 25 and above 70 years were excluded in the present analyses. The present analysis was restricted to the rural and urban sites (n=2492) RODAM participants. Specifically, 1043 participants from rural Ghana and 1449 from urban Ghana were used in this study.

Data collection for the study was standardized across the sites. Written informed consent was obtained from each participant prior to enrolment in the study. The respective ethics committees in Ghana and the three European countries approved the study protocols before data collection began. The response rate was 76% in rural Ghana and 74% in urban Ghana. In Ghana, participants were randomly drawn from a list of 30 enumeration areas in the Ashanti region based on the 2010 population census using the multistage random sampling. These enumeration areas came from two purposively selected urban cities (Kumasi and Obuasi) and 15 randomly selected rural communities in the Ashanti region. Selected health and community authorities were first identified, notified of the study and letters were sent giving detailed explanation of the study. We sent team members to stay among the communities to familiarize with them and organize mini clinics in the field. This lasted between 1-2 weeks depending on the sampled population and responsiveness of respondents.

In Ghana, questionnaires administration and physical examination were done at the same day/time. The participants were instructed to fast from 10.00 pm the night before the physical examination. For the current study, 2566 participants with data available on both questionnaire data and physical measurements were used. We excluded (n=74) individuals outside the RODAM age range of 25-70 years resulting in a data set of 2492 for analysis. These comprised 1,449 Urban Ghana and 1043 Rural Ghana. For the final analysis, individuals with no data on CKD status (n=42) were excluded.

For peer review only - http://bmjopen.bmj.com/site/about/guidelines.xhtml

Measurements

Demographic and lifestyle factors

Information on demographics, educational level, occupational level, wealth index and lifestyle factors (smoking and physical activity) were obtained by questionnaire. Physical examinations were performed with validated devices per standardized operational procedures across all study sites. Weight was measured in light clothing and without shoes with SECA 877 scales to the nearest 0.1 kg. Height was measured without shoes with a portable stadiometer (SECA 217) to the nearest 0.1 cm. Body mass index (BMI) was calculated as weight (kg) divided by height squared (m^2). Overweight was defined as BMI of ≥25 to <30 kg/m^2 and obesity as BMI ≥30 kg/m^2 [18]. Waist circumference was measured in cm at the midpoint between the lower rib and the upper margin of the iliac crest. Per participant all anthropometrics were measured twice by the same assessor and the average of the two measurements were used for analyses.

Covariates

Socioeconomic indicators used in this study were educational level, occupational status and level of wealth index. Educational level was determined based on self-reported highest educational qualification accomplished based on the Ghanaian educational system. Occupational level was determined based on self-reported current occupation if still employed or/and last occupation before retirement or student. The reported occupations were further coded according to the International Standard Classification of Occupations scheme (ISCO-08). Wealth index was determined using the World Health Organization (WHO) standard of wealth index classification. Wealth index was based on data collected in the Household Questionnaire. The questionnaire comprised of questions on household's ownership of several consumer items such as television, car, flooring material, toilet facilities etc. Each household was assigned a standard score for each asset. Wealth index was then expressed in five quintiles. The five quintiles were further categorized into three quintiles by combining the second and third quintiles due to small numbers [19]. All three SES constructs were further classified as low, medium and high SES and their relationship to each other tested.

Outcome: CKD prevalence

Participants were asked to bring an early morning urine sample for the analyses of albuminuria and creatinine levels. Urinary albumin concentration (in mg/L) was measured by an immunochemical turbidimetric method (Roche Diagnostics). Urinary creatinine concentration (in umol/L) was measured by a kinetic spectrophotometric method (Roche Diagnostics). Estimated glomerular filtration rate (eGFR)

was calculated using the CKDEPI (CKD Epidemiology Collaboration) creatinine equation [20]. Urinary albumin-creatinine ratio (ACR; expressed in mg/g) was calculated by taking the ratio between urinary albumin and urinary creatinine. eGFR and albuminuria were categorized according to the 2012 KDIGO (Kidney Disease: Improving Global Outcomes) classification [21]. eGFR was categorized as follows: G1, ≥ 90 mL/min/1.73 m^2 (normal kidney function); G2, 60 to 89 mL/min/1.73 m^2 (mildly decreased); G3a, 45 to 59 mL/min/1.73 m^2 (mildly to moderately decreased); G3b, 30 to 44 mL/min/1.73 m^2 (moderately to severely decreased); G4, 15 to 29 mL/min/1.73 m^2 (severely decreased); and G5, < 15 mL/min/1.73 m^2 (kidney failure). Albuminuria categories were derived from ACR and were as follows: A1, < 3mg/mmol (normal to mildly increased); A2, 3 to 30 mg/mmol (moderately increased); and A3, > 30mg/mmol (severely increased). CKD status was categorized according to severity of kidney disease (green, low risk; yellow, moderately increased risk; orange, high risk; and red, very high risk) using the combination of eGFR (G1-G5) and albuminuria (A1-A3) levels defined by the 2012 KDIGO guideline [22]. Due to the small number of participants in the very high risk category of CKD, high and very high risk groups were combined. Reduced eGFR was defined as eGFR < 60 mL/min/1.73 m^2. Because of the small number of participants in the severely increased albuminuria category, we defined albuminuria as ACR ≥3 mg/mmol by combining the moderately increased (A2) and severely increased (A3) categories.

Other variables

Blood pressure (BP) was measured three times using a validated semi-automated device (The Microlife WatchBP home) with appropriate cuffs in a sitting position after at least 5 min rest. The mean of the last two BP measurements was used in the analyses. Hypertension was defined as systolic BP ≥ 140 mmHg, and/or diastolic BP ≥ 90 mmHg, and/or being on antihypertensive medication treatment, and/or self-reported hypertension. Trained research assistants in the two sites collected fasting venous blood samples. All the blood samples were processed and aliquoted immediately (within one hour to maximum three hours of the vena puncture) after collection per standard operation procedures, and then temporarily stored at the local research location at −20°C. The separated samples were then transported to the local research centres laboratories, where they were checked, registered and stored at −80°C. To avoid intra-laboratory variability, the stored blood samples from the local research centres were transported to Berlin, Germany for biochemical analyses. Fasting plasma glucose concentration was measured using an enzymatic method (hexokinase). Type 2 diabetes was defined according to the WHO diagnostic criteria (fasting glucose ≥7.0 mmol/L, and/or current use of medication prescribed to treat diabetes, and/or self-reported diabetes) [23]. Concentration of total cholesterol was assessed using colorimetric test kits. All biochemical analyses were performed using an ABX Pentra 400 chemistry analyzer (ABX Pentra; Horiba ABX, Germany). Hypercholesterolemia was defined as total cholesterol level ≥ 6.22 mmol/L. Serum

For peer review only

creatinine concentration (in umol/L) was determined by a kinetic colorimetric spectrophotometric isotope dilution mass spectrometry–calibrated method (Roche Diagnostics). Biochemical analyses were subject to extensive quality checks including blinded serial measurements.

Patient and Public Involvement

Community leaders were involved in the recruitment of patients. These comprised of religious communities (churches and mosques), endorsement from local key leaders and establishing relationships with healthcare organizations. We also provided information on the study by involving the local media (radio and television stations). We sent letters to all selected health and community authorities to notify participants of the study. Team members were sent to the various community to stay among the community and organize mini clinics for a period of 1-2 weeks. Results of the study were disseminated through seminars, durbars and via radio and television stations.

Statistical methods

Participants' characteristics were expressed as absolute numbers and percentages for categorical variables and as means and standard deviations (SD) for continuous variables. CKD prevalence with 5% error bars were presented as bar graphs for each SES construct across rural and urban Ghana. Spearman's rank correlation was used to determine correlations between the three SES constructs. Odds ratios (ORs) and their corresponding 95% confidence intervals (CIs) were estimated by means of logistic regression analyses to study the odds of albuminuria (ACR>3 mg/mmol, A2-A3, moderately to severely increased albuminuria), reduced kidney function (eGFR< 60 mL/min/1.73 m^2, G3-G5 moderately to severely decreased kidney function) and increased CKD risk (high and very high CKD risk), with adjustments for potential covariates (age and sex). These covariates were adjusted for to account for their impact in the pathway of CKD incidence, prevalence and progression [24]. Model 1 was age and sex adjusted. The analyses were performed for the total population (using low educational level, low occupational status and low level of wealth index as reference categories). Further analysis was conducted using rural Ghana as reference. Model 1 was adjusted for age and sex while model 2 was adjusted for age, sex and educational level. Model 3 was adjusted for age, sex and occupational status while model 4 was adjusted for age, sex and level of wealth index ($p<0.05$). Tolerance test and variance inflation factor (VIF) showed very small degree of collinearity among SES predictors thus we adjusted for each of SES variables separately. Complete case analysis approach was used. All data available were included in the age-adjusted models. All analyses were performed using STATA, version 14.0 (StataCorp LP).

Results

For peer review only - http://bmjopen.bmj.com/site/about/guidelines.xhtml

Table 1 shows characteristics of study participants. Participants in rural Ghana were slightly older than those in urban Ghana. Female preponderance was observed in both rural and urban Ghana, though higher proportions were observed in urban Ghana. Individuals living in rural Ghana were generally less educated compared with those living in urban Ghana. There were slightly more individuals with low occupational status in urban Ghana compared with their peers in rural Ghana. People in urban Ghana were wealthier than their rural counterparts. Rural Ghanaians were more physically active compared with their urban peers. Smoking was low among Ghanaians though rural Ghanaians were more likely to smoke compared with their urban peers. Hypercholesterolemia was more prevalent in urban Ghana than in rural Ghana. Hypertension and type 2 diabetes were more prevalent in urban Ghanaians compared with those living in rural Ghana. Urban Ghanaians were markedly more obese compared with their rural peers. Except for eGFR, albuminuria and CKD risk prevalence rates were higher in urban Ghana compared with rural Ghana.

Table 1: Baseline characteristics by location

	Rural Ghana	Urban Ghana
Number of participants, N (%)	1043 (41.9)	1449 (58.1)
Mean age, years (SD)	46.5 (12.6)	45.2 (11.4)
Females, N (%)	638 (61.2)	1034 (71.4)
Educational level n (%)		
Low	555 (56.9)	614 (43.9)
Middle	311 (31.9)	547 (39.1)
High	108 (11.8)	239 (17.0)
Occupational status, n (%)		
Low	250 (25.7)	374 (26.7)
Middle	628 (64.5)	818 (58.4)
High	96 (9.8)	209 (14.9)
Wealth index, n (%)		
Low	449 (46.5)	368 (26.6)
Middle	276 (28.6)	416 (30.0)
High	241 (24.9)	602 (43.4)
Low physical activity, n (%)	663 (47.22)	592 (60.7)
Smoking, n (%)	22 (2.3)	14 (1.0)
Hypercholesterolemia, n (%)	78 (7.6)	270 (18.7)
Hypertension, n (%)	306 (29.3)	531 (36.7)
Diabetes, n (%)	53 (5.1)	153 (10.6)

For peer review only

Albuminuria, n (%)		
A1, Normal to mildly increased (ACR <3 mg/mmol)	930 (91.6)	1285 (89.1)
A2-A3, moderately to severely increased (ACR≥ 3 mg/mmol)	85 (8.4)	158 (10.9)
eGFR, n (%)		
G1-G2 (≥ 60 mL/min/1.73m^2)	989 (96.3)	1388 (96.3)
G3-G5 (<60 mL/min/1.73m^2)	38 (3.7)	54 (3.7)
CKD risk, n (%)		
Low risk (green)	916 (90.5)	1281 (88.9)
Moderately increased to very high risk (yellow to red)	96 (9.5)	160 (11.1)

Abbreviations: N, number of respondents; SD, standard deviation; eGFR, estimated glomerular filtration rate; ACR, albumin creatinine ratio; CKD, Chronic kidney disease

Figure 1 shows prevalence of CKD by level of education in urban and rural Ghana. Prevalence of CKD decreased with increasing levels of education in rural Ghana. Higher prevalence of CKD was observed among individuals with low educational level compared with those with middle and high educational level. However, those with high educational level in urban Ghana had higher prevalence of CKD compared with those with middle level education. For occupational status, prevalence of CKD was higher among individuals with low occupational status in urban Ghana. Similar patterns were observed in rural Ghana, however, those with higher occupational status had higher prevalence of CKD compared with those with middle occupational status (Figure 2). Figure 3 shows prevalence of CKD by level of wealth index. CKD prevalence among the levels of wealth index varied between urban and rural Ghana. Those with middle level wealth index had higher prevalence of CKD compared with those with low or high CKD prevalence in both rural and urban Ghana. CKD prevalence rate for low and high level wealth index in urban Ghana was the same while that of rural Ghana was slightly different.

For peer review only

In urban Ghana, high educational level was positively associated with high wealth index but inversely associated with occupation. In rural Ghana, high education was positively associated with high wealth index, but there was no significant association between education and occupation. High wealth index was inversely associated with high occupational status in both rural and urban Ghana (Table 2).

Table 2: Relationship between SES constructs (educational, occupational level and wealth index) by urban rural Ghana

Correlation matrix	Educational level	Occupational level	Wealth index	SES
**Urban Ghana**				
Educational level	1.000			
Occupational status	-0.115*	1.000		
Wealth Index	0.294*	-0.126*	1.000	
Composite SES	0.576*	-0.024	0.937*	1.000
**Rural Ghana**				
Educational level	1.000			
Occupational status	0.017	1.000		
Wealth Index	0.219*	-0.135*	1.000	
SES	0.504*	0.029	0.934*	1.000

Significant at 1%, Spearman's correlation

For peer review only

Table 3 shows association between level of education, occupational status, level of wealth index and prevalence of CKD. After adjusting for age and sex, we observed no significant association between SES indicators (educational level, occupational status and wealth index) and CKD in urban Ghana. In rural Ghana, whereas educational level and occupational status were not associated with CKD prevalence, high wealth index was significantly associated with higher odds of reduced eGFR.

Table 3: **Association of SES indicators (educational level, occupational status and wealth index level) with albuminuria, reduced eGFR and CKD risk**

	Albuminuria (ACR ≥ 3 mg/mmol)		eGFR < 60 mL/min/1.73 m2		High to very high CKD risk (KDIGO, 2012)	
	n (%)	OR (95% CI) Model 1	n (%)	OR (95% CI) Model 1	n cases (%)	OR (95% CI) Model 1
**Education**						
**Urban Ghana**						
Low	612 (14.7)	1.00 (Reference)	612 (4.1)	1.00 (Reference)	612 (14.1)	1.00 (Reference)
Middle	546 (7.8)	0.51 (0.34-0.76)	546 (3.7)	1.12 (0.59-2.12)	545 (8.1)	0.59 (0.39-0.89)
High	238 (8.4)	0.53 (0.31-0.91)	238 (3.4)	0.91 (0.37-2.19)	238 (10.9)	0.83 (0.51-1.38)
**Rural Ghana**						
Low	540 (8.7)	1.00 (Reference)	548 (3.8)	1.00 (Reference)	538 (9.5)	1.00 (Reference)
Middle	301 (6.3)	0.89 (0.51-1.59)	303 (3.9)	1.69 (0.77-3.66)	300 (8.7)	1.33 (0.79-2.25)
High	105 (3.8)	0.66 (0.23-1.95)	107 (2.8)	1.28 (0.35-4.71)	105 (3.8)	0.69 (0.23-2.02)
**Occupational status**						
**Urban Ghana**						
Low	207 (10.1)	1.00 (Reference)	207 (6.8)	1.00 (Reference)	207 (12.1)	1.00 (Reference)
Middle	817 (11.1)	1.50 (0.88-2.83)	817 (3.6)	1.15 (0.56-2.35)	816 (11.6)	1.37 (0.84-2.56)
High	373 (11.0)	1.57 (0.89-2.53)	373 (2.7)	1.02 (0.41-2.52)	373 (9.7)	1.21 (0.68-2.14)
**Rural Ghana**						

For peer review only

Low	95 (10.5)	1.00 (Reference)	96 (10.4)	1.00 (Reference)	95 (14.7)	1.00 (Reference)
Middle	610 (6.7)	0.65 (0.31-1.37)	619 (3.1)	0.37 (0.16-0.85)	608 (7.6)	0.55 (0.28-1.08)
High	241 (8.3)	0.99 (0.43-2.28)	243 (2.9)	0.51 (0.18-1.44)	240 (9.2)	0.94 (0.44-2.01)
**Wealth index**						
**Urban Ghana**						
Low	367 (11.2)	1.00 (Reference)	367 (3.5)	1.00 (Reference)	367 (10.1)	1.00 (Reference)
Middle	414 (12.3)	1.12 (0.73-1.74)	414 (3.9)	1.30 (0.61-2.80)	413 (13.1)	1.45 (0.93-2.27)
High	601 (9.8)	0.82 (0.55-1.25)	600 (3.8)	1.13 (0.55-2.31)	600 (10.8)	1.11 (0.72-1.71)
**Rural Ghana**						
Low	441 (7.9)	1.00 (Reference)	446 (3.1)	1.00 (Reference)	441 (8.4)	1.00 (Reference)
Middle	264 (8.7)	1.13 (0.65-1.98)	269 (3.7)	1.22 (0.52-2.84)	262 (10.3)	1.31 (0.77-2.25)
High	234 (5.6)	0.78 (0.40-1.53)	235 (5.1)	**2.38 (1.03-5.47)**	233 (7.7)	1.16 (0.63-2.14)

Model 1, adjusted for age and sex; Abbreviations: CI, confidence interval; ACR, albumin creatinine ration; eGFR, estimated glomerular filtration rate; CKD, chronic kidney disease; OR, odds ratio, n= total number of individuals in rural and urban Ghana among the various levels of SES constructs; %, proportion of individuals with CKD among the various levels of SES constructs in rural and urban Ghana.

For peer review only

Table 4 shows the contribution of all three SES constructs to rural and urban CKD prevalence differences. The odds of albuminuria and CKD risk was significantly higher in urban Ghana compared with rural Ghana. The higher rate of CKD observed in urban Ghana was not explained by the higher SES of that population as compared to their rural counterparts.

Table 4: Contribution of SES indicators to rural-urban differences in albuminuria, reduced eGFR and CKD risk

		OR (95% CI) Model 1	OR (95% CI) Model 2	OR (95% CI) Model 3	OR (95% CI) Model 4
**Albuminuria (ACR ≥ 3 mg/mmol**					
Sites	n cases (%)				
Urban Ghana	1,443 (10.9)	**1.37 (1.03-1.81)**	**1.70 (1.25-2.31)**	**1.55 (1.15-2.10)**	**1.62 (1.18-2.19)**
Rural Ghana	1,015 (8.4)	1.00 (Reference)	1.00 (Reference)	1.00 (Reference)	1.00 (Reference)
**eGFR < 60 mL/min/1.73 m2**					
**Sites**	n cases (%)				
Urban Ghana	1,442 (3.7)	1.27 (0.82-1.97)	1.20 (0.76-1.89)	1.18 (0.79-1.86)	1.12 (0.70-1.78)
Rural Ghana	1,027 (3.7)	1.00 (Reference)	1.00 (Reference)	1.00 (Reference)	1.00 (Reference)
**High to very high CKD risk**					
**Sites**	n cases (%)				
Urban Ghana	1,441 (11.1)	**1.23 (1.01-1.62)**	**1.44 (1.07-1.93)**	**1.38 (1.03-1.84)**	**1.36 (1.01-1.83)**
Rural Ghana	1,012 (9.46)	1.00 (Reference)	1.00 (Reference)	1.00 (Reference)	1.00 (Reference)

Model 1: adjusted for age and sex; Model 2: adjusted for age, sex and education level; Model 3: adjusted for age, sex and occupational status; Model 4: adjusted for age, sex and wealth index; Abbreviations: CI, confidence interval; ACR, albumin creatinine ration; eGFR, estimated glomerular filtration rate; CKD, chronic kidney disease; OR, odds ratio, n=number of participants. n= total number of individuals in rural and urban Ghana; %, proportion of individuals with CKD among urban and rural Ghana.

For peer review only

Discussion

Key findings

Our study findings show no association between all three SES constructs and the prevalence of CKD in both rural and urban Ghana except for wealth index in rural Ghana, with the risk of CKD being higher in the wealthier populations. The higher rate of CKD observed in urban Ghana could not be attributed to the higher SES of that population compared to their rural counterparts.

Discussion of key findings

Association of SES with CKD in rural and urban Ghana

Our study did not find any significant associations between all three SES constructs and CKD among rural and urban Ghana except for wealth index in rural Ghana. The positive association observed between wealth index in rural Ghana may be due to a number of reasons. A comparison of the three SES constructs showed higher educational level to be associated with wealth index in both rural and urban Ghana but not occupational level. This seems to suggest that occupational level may not be adequately capturing the SES status of individuals living in these settings in relation to CKD. For example, Masthi et al, compared different SES scales in rural and urban India and concluded that Standard of Living Index (SLI) scale was more accurate for classification of SES in urban and rural setting [25]. Our finding is consistent with other studies [6 26] which reported no association between SES and CKD in high-income countries and LMICs, but in contrast with other studies [2-4 27] that found positive associations between SES and CKD. The reasons for our current finding are unclear. However, it has been suggested that these inconsistent associations may be due to the varying pathways through which the effect of SES on health status is mediated. For example, at a given educational level marked ethnic differences have been reported. Additionally, similar differences were observed for wealth status at a given income level [28-30].

Contribution of SES to observed CKD risk differences between rural and urban Ghana

We observed higher rates of CKD in urban Ghana compared with rural Ghana, as expected. The observed higher rates of CKD in our study were not explained by the higher SES of that population as compared to their rural counterparts. Our results indicate that this is due to the lack of a clear difference in the SES distribution of rural and urban Ghana observed in this study, as well as to the lack of associations between SES and CKD. Consistent with our findings, in a study conducted in Northern Tanzania SES did not explain increased risk of CKD in urban Tanzania [26]. The lack of associations between SES and CKD

For peer review only - http://bmjopen.bmj.com/site/about/guidelines.xhtml

could probably partly be explained by the process of epidemiological transition in relation to the "diffusion theory" of ischemic heart disease mortality. This theory attributes the commencement of ischemic heart disease to individuals in the high SES group due to their ability to afford behaviours (smoking, alcohol and sedentary lifestyles) which increased risk of ischemic heart disease. The lower SES groups were later affected partially because of improved living standards, imitation and urbanization. The higher SES groups were the first to embrace behavioural changes required to decrease the risk of ischemic heart disease and this resulted in reversing the gradient [31]. The rapid urbanization of some rural communities in the Ashanti region of Ghana and the imitation of urban lifestyle could account for our finding. Also, it could be that whereas the high SES group in urban Ghana has already embraced favourable behavioural changes, those in rural Ghana are yet to do so [32]. This explains the observed association of wealth index with CKD in rural Ghana but not in urban Ghana. The complexities of influence of SES on prevalence and progression of CKD and the differential prevalence of established risk factors (diabetes, obesity and hypertension) in rural and urban Ghana may also contribute to the different associations of SES with CKD prevalence observed in rural and urban Ghana. In our study, the prevalence rates of hypercholesterolemia, hypertension and type 2 diabetes were substantially higher in urban Ghana compared with that of rural Ghana. Also, the interplay of other less understood or researched factors (e.g., exposure to nephrotoxins, herbal medications, sepsis) may be influencing the pathway in which SES influences CKD prevalence and progression.

Strength and limitation

Our study presents several strengths. First, we used well-standardized study protocols across rural and urban Ghana. Our study is also the first in Africa to use all three categories of CKD definition (albuminuria, reduced eGFR and CKD risk) by KDIGO 2012 in assessing association of SES with CKD in rural and urban setting, this provides a more detailed information on CKD outcomes. The limitation of intra laboratory variability in earlier studies was eliminated using the same standard operating procedures in the same laboratory for running all samples for both rural and urban Ghana. The use of three constructs of SES in this study also provides a much better holistic approach to assessing SES. Also, the distribution of SES in our study reflect on the national data allowing for generalization of our findings. Our study was limited by the use of cross sectional design which prevented us from determining causality between predictors and CKD progression.

For peer review only - http://bmjopen.bmj.com/site/about/guidelines.xhtml

Conclusion

All three SES constructs appear not to be associated with prevalence of CKD in urban and rural Ghana except for wealth index in rural Ghana. The observed higher prevalence of CKD in urban Ghana was not explained by the higher SES in urban Ghana. Our study seems to suggest that other non-traditional factors such as nephrotoxins, herbal medications and misuse of over the counter drugs may play a role and underscores the need to further explore these factors.

Acknowledgement

The authors are very grateful to the research assistants, interviewers and other staff of the five research locations who took part in gathering the data and the Ghanaian volunteers in all the participating RODAM sites. We gratefully acknowledge the advisory board members for their valuable support in shaping the RODAM study methods and the Academic Medical Centre Biobank for their support in biobank management and high-quality storage of collected samples.

Contributors

My co-authors have all contributed substantially to this manuscript and approve of this submission. Research idea and study design: DNA, CA, KS, DA, EB, KM, JA; data acquisition and curation: DNA, CA, EB, KM, data analysis/interpretation: DNA, CA, KS, DA, EB, KM, LS, JA, EOD, KKB, FPM, ID, JS, SB, ADA; statistical analysis: DNA, CA, KS. DNA, CA, KS, DA, EB, KM, LS, JA, EOD, KKB, FPM, ID, JS, SB, ADA contributed important intellectual content during manuscript drafting or revision and accepts accountability for the overall work by ensuring that questions pertaining to the accuracy or integrity of any portion of the work are appropriately investigated and resolved. DNA and CA takes responsibility that this study has been reported honestly, accurately, and transparently; that no important aspects of the study have been omitted; and that any discrepancies from the study as planned have been explained.

Funding

This work was supported by the European Commission under the Framework Programme (Grant Number: 278901). The funders had no role in study design, data collection and analysis, decision to

For peer review only

For peer review only - http://bmjopen.bmj.com/site/about/guidelines.xhtml

publish, or preparation of the manuscript. The Wellcome Trust supported Professor Smeeth's contribution, grant number WT082178. Professor Joachim Spranger was supported by the DZHK (German Center for cardiovascular research) and the Berlin Institute of Health (BIH).

Competing interest: I have communicated with all my co-authors and obtained their full disclosures. My co-authors and I declare no conflicts of interest.

Patient Consent: None declared

Ethics approval: IRBs at each participating site.

Data sharing statement: Data are available from the RODAM research cohort, a third party. Dr. Eric Beune affiliated with the RODAM research cohort and a co-author of this paper in accordance with the RODAM requirements for collaboration. Dr. Beune is the Data Collection Coordinator of RODAM and may be contacted with further questions (e.j.beune@amc.uva.nl). Additionally, researchers interested in further collaboration with RODAM may see the following URL: http://www.rod-am.eu/

For peer review only

For peer review only - http://bmjopen.bmj.com/site/about/guidelines.xhtml

References

1. Patzer RE, McClellan WM. Influence of race, ethnicity and socioeconomic status on kidney disease. *Nature Reviews Nephrology* 2012;8(9):533-41.
2. Merkin SS, Roux AVD, Coresh J, et al. Individual and neighborhood socioeconomic status and progressive chronic kidney disease in an elderly population: The Cardiovascular Health Study. *Soc Sci Med* 2007;65(4):809-21.
3. Bello AK, Peters J, Rigby J, et al. Socioeconomic status and chronic kidney disease at presentation to a renal service in the United Kingdom. *Clin J Am Soc Nephrol* 2008;3(5):1316-23.
4. Crews DC, Charles RF, Evans MK, et al. Poverty, race, and CKD in a racially and socioeconomically diverse urban population. *Am J Kidney Dis* 2010;55(6):992-1000.
5. Shoham DA, Vupputuri S, Roux AVD, et al. Kidney disease in life-course socioeconomic context: the Atherosclerosis Risk in Communities (ARIC) Study. *Am J Kidney Dis* 2007;49(2):217-26.
6. Byrne C, Nedelman J, Luke RG. Race, socioeconomic status, and the development of end-stage renal disease. *Am J Kidney Dis* 1994;23(1):16-22.
7. Popkin BM. The shift in stages of the nutrition transition in the developing world differs from past experiences! *Public Health Nutr* 2002;5(1A):205-14.
8. Du S, Lu B, Zhai F, et al. A new stage of the nutrition transition in China. *Public Health Nutr* 2002;5(1a):169-74.
9. Kim S, Symons M, Popkin BM. Contrasting socioeconomic profiles related to healthier lifestyles in China and the United States. *Am J Epidemiol* 2004;159(2):184-91.
10. Adler NE, Ostrove JM. Socioeconomic status and health: what we know and what we don't. *Ann N Y Acad Sci* 1999;896(1):3-15.
11. Anderson NB, Armstead CA. Toward understanding the association of socioeconomic status and health: A new challenge for the biopsychosocial approach. *Psychosom Med* 1995;57(3):213-25.
12. Feinstein JS. The relationship between socioeconomic status and health: a review of the literature. *The Milbank Quarterly* 1993:279-322.
13. Samuel P, Antonisamy B, Raghupathy P, et al. Socio-economic status and cardiovascular risk factors in rural and urban areas of Vellore, Tamilnadu, South India. *Int J Epidemiol* 2012;41(5):1315-27.
14. Christie S, Fone DL. Does car ownership reflect socio-economic disadvantage in rural areas? A cross-sectional geographical study in Wales, UK. *Public Health* 2003;117(2):112-16.
15. Agyemang C, Beune E, Meeks K, et al. Rationale and cross-sectional study design of the Research on Obesity and type 2 Diabetes among African Migrants: the RODAM study. *BMJ open* 2015;4(3):e004877.
16. Addo J, Agyemang C, Aikins Ad-G, et al. Association between socioeconomic position and the prevalence of type 2 diabetes in Ghanaians in different geographic locations: the RODAM study. *J Epidemiol Community Health* 2017:jech-2016-208322.
17. Adjei DN, Stronks K, Adu D, et al. Relationship between educational and occupational levels, and Chronic Kidney Disease in a multi-ethnic sample-The HELIUS study. *PLoS One* 2017;12(11):e0186460.

For peer review only

For peer review only - http://bmjopen.bmj.com/site/about/guidelines.xhtml

18. Consultation WE. Waist circumference and waist-hip ratio. *Report of a WHO Expert Consultation Geneva: World Health Organization* 2008:8-11.
19. Rutstein SO, Johnson K, MEASURE OM. The DHS wealth index: ORC Macro, MEASURE DHS 2004.
20. Stevens LA, Schmid CH, Greene T, et al. Comparative performance of the CKD epidemiology collaboration (CKD-EPI) and the modification of diet in renal disease (MDRD) study equations for estimating GFR levels above 60 mL/min/1.73 m 2. *Am J Kidney Dis* 2010;56(3):486-95.
21. Eknoyan G, Lameire N, Eckardt K, et al. KDIGO 2012 clinical practice guideline for the evaluation and management of chronic kidney disease. *Kidney Int* 2013;3:5-14.
22. KDIGO G. Work Group. KDIGO clinical practice guideline for glomerulonephritis. *Kidney inter, Suppl* 2012;2:139-274.
23. Association AD. Standards of medical care in diabetes—2014. *Diabetes Care* 2014;37(Supplement 1):S14-S80.
24. Collins AJ, Foley RN, Herzog C, et al. US Renal Data System 2010 Annual Data Report. *American journal of kidney diseases: the official journal of the National Kidney Foundation* 2011;57(1 Suppl 1):A8, e1.
25. Masthi NR, Gangaboraiah PK. An exploratory study on socio economic status scales in a rural and urban setting. *Journal of family medicine and primary care* 2013;2(1):69.
26. Stanifer JW, Maro V, Egger J, et al. The epidemiology of chronic kidney disease in Northern Tanzania: a population-based survey. *PLoS One* 2015;10(4):e0124506.
27. Bruce MA, Beech BM, Crook ED, et al. Association of socioeconomic status and CKD among African Americans: the Jackson Heart Study. *Am J Kidney Dis* 2010;55(6):1001-08.
28. Braveman PA, Cubbin C, Egerter S, et al. Socioeconomic status in health research: one size does not fit all. *JAMA* 2005;294(22):2879-88.
29. Gilthorpe MS, Wilson RC. Rural/urban differences in the association between deprivation and healthcare utilisation. *Soc Sci Med* 2003;57(11):2055-63.
30. Choi AI, Weekley CC, Chen S-C, et al. Association of educational attainment with chronic disease and mortality: the Kidney Early Evaluation Program (KEEP). *Am J Kidney Dis* 2011;58(2):228-34.
31. Mackenbach JP, Cavelaars A, Kunst AE, et al. Socioeconomic inequalities in cardiovascular disease mortality. An international study. *Eur Heart J* 2000;21(14):1141-51.
32. McKay L, Macintyre S, Ellaway A. Migration and health: a review of the international literature. MRC Social and Public Health Sciences Unit, Occasional Paper No. 12. *Glasgow: Medical Research Council[Links]* 2003

For peer review only - http://bmjopen.bmj.com/site/about/guidelines.xhtml

Legend for figures

Figure 1: Prevalence of chronic kidney disease (CKD) across level of education among urban and rural participants Definitions according to 2012 KDIGO (Kidney Disease: Improving Global Outcomes) guideline. CKD was defined as being in moderately increased risk, high-risk, or very high-risk groups.

Figure 2: Prevalence of chronic kidney disease (CKD) across occupational status among urban and rural participants. Definitions according to 2012 KDIGO (Kidney Disease: Improving Global Outcomes) guideline. CKD was defined as being in moderately increased risk, high-risk, or very high-risk groups.

Figure 3: Prevalence of chronic kidney disease (CKD) across wealth index categories among urban and rural participants. Definitions according to 2012 KDIGO (Kidney Disease: Improving Global Outcomes) guideline. CKD was defined as being in moderately increased risk, high-risk, or very high-risk groups.

For peer review only

For peer review only - http://bmjopen.bmj.com/site/about/guidelines.xhtml

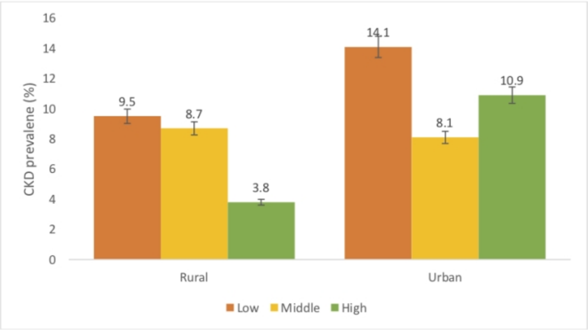

Figure 1: Prevalence of chronic kidney disease (CKD) across level of education among urban and rural participants Definitions according to 2012 KDIGO (Kidney Disease: Improving Global Outcomes) guideline. CKD was defined as being in moderately increased risk, high-risk, or very high-risk groups

Figure 1: Prevalence of chronic kidney disease (CKD) across level of education among urban and rural participants Definitions according to 2012 KDIGO (Kidney Disease: Improving Global Outcomes) guideline. CKD was defined as being in moderately increased risk, high-risk, or very high-risk groups

215x279mm (150 x 150 DPI)

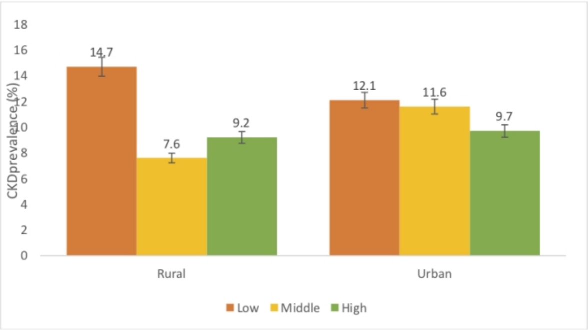

Figure 2: Prevalence of chronic kidney disease (CKD) across occupational status among urban and rural participants. Definitions according to 2012 KDIGO (Kidney Disease: Improving Global Outcomes) guideline. CKD was defined as being in moderately increased risk, high-risk, or very high-risk groups

Figure 2: Prevalence of chronic kidney disease (CKD) across occupational status among urban and rural participants. Definitions according to 2012 KDIGO (Kidney Disease: Improving Global Outcomes) guideline. CKD was defined as being in moderately increased risk, high-risk, or very high-risk groups

215x279mm (150 x 150 DPI)

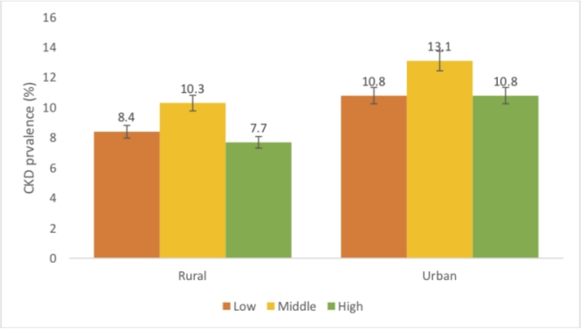

Figure 3: Prevalence of chronic kidney disease (CKD) across wealth index categories among urban and rural participants. Definitions according to 2012 KDIGO (Kidney Disease: Improving Global Outcomes) guideline. CKD was defined as being in moderately increased risk, high-risk, or very high-risk groups

Figure 3: Prevalence of chronic kidney disease (CKD) across wealth index categories among urban and rural participants. Definitions according to 2012 KDIGO (Kidney Disease: Improving Global Outcomes) guideline. CKD was defined as being in moderately increased risk, high-risk, or very high-risk groups

215x279mm (150 x 150 DPI)

A CROSS-SECTIONAL STUDY OF ASSOCIATION BETWEEN SOCIOECONOMIC INDICATORS AND CHRONIC KIDNEY DISEASE IN RURAL-URBAN GHANA-THE RODAM STUDY

Journal:	*BMJ Open*
Manuscript ID	bmjopen-2018-022610.R1
Article Type:	Research
Date Submitted by the Author:	18-Jul-2018

Complete List of Authors:	Adjei Nana, David ; University of Ghana, Department of Medical Laboratory Sciences; University of Amsterdam, Department of Public Health<br>Stronks, Karien; Academic Medical Center , Department of Public Health<br>Adu, Dwomoa; Korle-bu Teaching Hospital, Department of Medicine<br>Beune, Erik; AMC<br>Meeks, Karlijn; AMC, Public Health<br>Smeeth, Liam; London School of Hygiene and Tropical Medicine,<br>Addo, Juliet; London School of Hygiene and Tropical Medicine, Non Communicable Disease Epidemiology<br>Owusu-Dabo, Ellis; Kwame Nkrumah University of Science and Technology, Kumasi Centre for Collaborative Research in Tropical Medicine<br>Klipstein-Grobusch, Kerstin; 1 Julius Global Health, Julius Center for Health Sciences and Primary Care, University Medical Centre Utrecht, Utrecht, The Netherlands<br>Mockenhaupt, Frank; Charité – University Medicine, Berlin, Institute of Tropical Medicine and International Health<br>Danquah, Ina; German Institute of Human Nutrition, Molecular Epidemiology; Charite Universitatsmedizin Berlin, Institute for Social Medicine, Epidemiology and Health Economy<br>Spranger, Joachim; Department of Endocrinology and Metabolism, 1. Charité-University Medicine Berlin, Berlin, Germany.<br>Bahendeka, Silver; 1. MKPGMS - Uganda Martyrs University<br>De-Graft Aikins, Ama; University of Ghana, Regional Institute for Population Studies<br>Agyemang, Charles; Academic Medical centre, University of Amsterdam, Department of Public Health

**Primary Subject Heading**:	Epidemiology
Secondary Subject Heading:	Public health, Epidemiology
Keywords:	Chronic Kidney Disease, Socioeconomic status, Health inequalities, RODAM study, rural, urban

SCHOLARONE™
Manuscripts

A CROSS SECTIONAL STUDY OF ASSOCIATION BETWEEN SOCIOECONOMIC INDICATORS AND CHRONIC KIDNEY DISEASE IN RURAL-URBAN GHANA-THE RODAM STUDY

David N. Adjei, MSc[1,2]; Karien Stronks, MSc, PhD[1]; Dwomoa Adu, MD[3]; Erik Beune, MSc, PhD [1]; Karlijn Meeks, MSc [1]; Liam Smeeth, MD, PhD[4] ; Juliet, Addo, MD, PhD [4]; Ellis Owuso-Dabo, MSc, PhD [5], Kerstin Klipstein-Grobusch, MSc, PhD [6,7]; Frank P. Mockenhaupt, MD, PhD [8] ; Ina, Danquah, MSc, PhD [9,10] ; Joachim, Spranger, MD, PhD [11,12,13]; Silver Bahendeka, MD, PhD[14]; Ama de-Graft, Aikins, MSc, PhD[15]; Charles Agyemang, MPH, PHD[1]

1. Department of Public Health, Academic Medical Center, University of Amsterdam, Amsterdam Public Health Research Institute, Amsterdam, The Netherlands.
2. Department of Medical Laboratory Sciences, School of Biomedical and Allied Health Sciences, College of Health Sciences, University of Ghana, Accra, Ghana.
3. Department of Medicine, School of Medicine and Dentistry, University of Ghana and Korle-Bu Teaching Hospital, Accra, Ghana.
4. Department of Non-communicable Disease Epidemiology, London School of Hygiene and Tropical Medicine, London, United Kingdom.
5. Kumasi Centre for collaborative Research, KNUST, Kumasi, Ghana
6. Julius Global Health, Julius Center for Health Sciences and Primary Care, University Medical Centre, Utrecht University, The Netherlands
7. Division of Epidemiology & Biostatistics, School of Public Health, Faculty of Health Sciences, University of the Witwatersrand, Johannesburg, South Africa.
8. Institute of Tropical Medicine and International Health, Charité – University Medicine Berlin, Augustenburger Platz 1, 13353 Berlin, Germany.
9. Department of Molecular Epidemiology, German Institute of Human Nutrition Potsdam-Rehbrücke, Nuthetal, Germany.
10. Charité - Universitaetsmedizin Berlin, Institute for Social Medicine, Epidemiology and Health Economics, Berlin, Germany.
11. Department of Endocrinology and Metabolism, Charité-University Medicine Berlin, Berlin, Germany.
12. German Centre for Cardiovascular Research (DZHK), Berlin, Germany.
13. Center for Cardiovascular Research (CCR), Charité-University Medicine Berlin, Berlin, Germany.
14. MKPGMS - Uganda Martyrs University, Kampala, Uganda.
15. Regional Institute for Population Studies, University of Ghana, Legon, Ghana.

Address correspondence to David Nana Adjei, MSc, Department of Public Health, Academic Medical Centre, University of Amsterdam, Meibergdreef 9, 1105 AZ Amsterdam, the Netherlands, School Biomedical and Allied Health Sciences, Medical Laboratory Sciences, University of Ghana, E-mail: dna@chs.edu.gh, d.n.adjei@amc.uva.nl, Tel: +233236717850

For peer review only - http://bmjopen.bmj.com/site/about/guidelines.xhtml

Abstract

Objectives: Studies from high income countries suggest higher prevalence of Chronic Kidney Disease (CKD) among individuals in low socio economic groups. However, some studies from low and middle-income countries (LMICs) show the reverse pattern among those in high socioeconomic groups. It is unknown which pattern applies to individuals living in rural and urban Ghana. We therefore assessed the association between Socio-Economic Status (SES) indicators and CKD in rural and urban Ghana and to what extent the higher SES of people in urban areas of Ghana could account for differences in CKD between rural and urban populations.

Methods: We used baseline data from multi-centre Research on Obesity and Diabetes among African Migrants (RODAM) study. A sample of 2492 adults (Rural Ghana, 1043, Urban Ghana, 1,449) aged 25 to 70 years living in Ghana. Three CKD outcomes were considered using the 2012 KDIGO (Kidney Disease: Improving Global Outcomes) severity of CKD classification: albuminuria (albumin-creatinine ratio ≥ 3 mg/mmol (category ≥ A2)); reduced glomerular filtration rate (eGFR < 60 mL/min/1.73 m2 (category ≥ G3)) and high to very high CKD risk based on the combination of these two.

Results: All three SES indicators were not associated with CKD in both rural and urban Ghana after age and sex adjustment except for rural Ghana where high wealth index was significantly associated with higher odds of reduced eGFR (AOR, 2.38; 95% C.I. 1.03-5.47). The higher rate of CKD observed in urban Ghana was not explained by the higher SES of that population.

Conclusion: SES indicators were not associated with prevalence of CKD except for wealth index and reduced eGFR in rural Ghana. Consequently, the higher SES did not account for the increased rate of CKD among urban dwellers suggesting the need to identify other factors that may be driving this.

Index Words: Chronic kidney disease; socioeconomic status; health inequalities; risk factor; ethnic minority groups; migrants; RODAM study, Ghana

For peer review only

For peer review only - http://bmjopen.bmj.com/site/about/guidelines.xhtml

Strengths and limitation of the study

- The use of well-standardized study protocols across rural and urban Ghana eliminated intra protocol variability.

- **Our study is also the first in Africa to use all three categories of CKD definition (albuminuria, reduced eGFR and CKD risk) by KDIGO 2012 in assessing association of SES with CKD in rural and urban setting. This provides a more detailed information on CKD outcomes.**

- The limitation of intra laboratory variability in earlier studies was eliminated using the same standard operating procedures in the same laboratory for running all samples for both rural and urban Ghana.

- The use of three constructs of SES (educational level, occupational level and wealth index) in this study also provides a much better holistic approach to assessing SES association with CKD.

- Our study was limited by the use of cross sectional design which prevented us from determining causality between predictors and CKD progression.

For peer review only

For peer review only - http://bmjopen.bmj.com/site/about/guidelines.xhtml

Introduction

In general, individuals in lower socio-economic status (SES) groups have been shown to suffer more frequently from Chronic Kidney Disease (CKD), often progressing to End Stage Renal Disease (ESRD), and associated with inadequate dialysis treatment, reduced access to kidney transplantation and poor health outcomes [1]. Recent studies have consistently found low SES to be associated with higher risk of CKD among people of African origin [2-5].

However, in some settings the well-known inverse association between SES and CKD seems to be absent, or even reversed. For example, Bryne et al. did not find an association between SES and End Stage Renal Disease [6]. Other studies have found a positive association between SES and CKD [7 8]. Specifically, as SES improved, unhealthful lifestyle (unhealthy diet, physical inactivity, smoking and alcohol consumption) increased in China while that of the United States decreased with improved SES [9]. People with higher incomes, in these contexts, can afford a western lifestyle, which is more readily available in the urban areas than in the rural areas. There is therefore an interaction between individual SES and environmental factors, such as food and sedentary life style in such populations [10-12]. Consequently, in those settings, people with a higher SES might have higher CKD risk.
In urban areas, the population in general has a higher SES than in rural areas [13]. For example, individuals with higher educational level migrate from rural areas to find higher occupations matching their higher education to improve on their wealth. If indeed a positive association between SES and CKD is observed in LMICs, this might underlie the well-known health differences between urban and rural areas, with urban areas having an increased risk of CKD [14]. So far, it is unknown whether the reversed SES gradient (higher risk in high SES group) might explain the higher burden of CKD in urban areas as compared to rural areas in Africa.

In view of this, we assessed the association of SES with CKD in rural and urban Ghana and studied what extent the higher SES of people in urban areas could account for differences in CKD between rural and urban populations.

For peer review only - http://bmjopen.bmj.com/site/about/guidelines.xhtml

Methods

Study population and study design

In the present analyses, data from the RODAM (**R**esearch on **O**besity & **D**iabetes among **A**frican **M**igrants) study, a multi-centre cross-sectional study were used. The rationale, conceptual framework, design and methodology of the RODAM study have been described in detail elsewhere [15 16]. As the Healthy Life in an Urban Setting (HELIUS) study conducted among Ghanaian migrants living in Amsterdam did not find any associations between SES and CKD [17] the current study focused on rural and urban Ghana (Ashanti region of Ghana). The RODAM study was conducted from 2012 to 2015 and it comprised of individuals aged 25-70 years living in rural and urban Ghana and Ghanaian migrants in Europe. All participants below 25 and above 70 years were excluded in the present analyses. The present analysis was restricted to the rural and urban sites (n=2492) RODAM participants. Specifically, 1043 participants from rural Ghana and 1449 from urban Ghana were used in this study.

Data collection for the study was standardized across the sites. Written informed consent was obtained from each participant prior to enrolment in the study. The respective ethics committees in Ghana and the three European countries approved the study protocols before data collection began. Specifically, we obtained ethical clearance in Ghana from (School of Medical Sciences/Komfo Anokye Teaching Hospital Committee on Human Research, Publication & Ethical Review Board, in the Netherlands, (Institutional Review Board of the AMC, University of Amsterdam), in Germany, (Ethics Committee of Charite-Universitätsmedizin) and the UK (London School of Hygiene and Tropical Medicine Research Ethics Committee) before data collection began in each country.

The response rate was 76% in rural Ghana and 74% in urban Ghana. In Ghana, participants were randomly drawn from a list of 30 enumeration areas in the Ashanti region based on the 2010 population census using the multistage random sampling. These enumeration areas came from two purposively selected urban cities (Kumasi and Obuasi) and 15 randomly selected rural communities in the Ashanti region. Selected health and community authorities were first identified, notified of the study and letters were sent giving detailed explanation of the study. We sent team members to stay among the communities to familiarize with them and organize mini clinics in the field. This lasted between 1-2 weeks depending on the sampled population and responsiveness of respondents.

In Ghana, questionnaires administration and physical examination were done at the same day/time. The participants were instructed to fast from 10.00 pm the night before the physical examination. For the current study, 2566 participants with data available on both questionnaire data and physical measurements

For peer review only - http://bmjopen.bmj.com/site/about/guidelines.xhtml

were used. We excluded (n=74) individuals outside the RODAM age range of 25-70 years resulting in a data set of 2492 for analysis. These comprised 1,449 Urban Ghana and 1043 Rural Ghana. For the final analysis, individuals with no data on CKD status (n=42) were excluded.

Measurements

Covariates

Demographic and lifestyle factors

Information on demographics, educational level, occupational level, wealth index and lifestyle factors (smoking and physical activity) were obtained by questionnaire. Physical examinations were performed with validated devices per standardized operational procedures across all study sites. Weight was measured in light clothing and without shoes with SECA 877 scales to the nearest 0.1 kg. Height was measured without shoes with a portable stadiometer (SECA 217) to the nearest 0.1 cm. Body mass index (BMI) was calculated as weight (kg) divided by height squared (m^2). Overweight was defined as BMI of ≥25 to <30 kg/m^2 and obesity as BMI ≥30 kg/m^2 [18]. Per participant all anthropometrics were measured twice by the same assessor and the average of the two measurements were used for analyses.

Predictor: SES

Socioeconomic indicators used in this study were educational level, occupational status and level of wealth index. Educational level was determined based on self-reported highest educational qualification accomplished based on the Ghanaian educational system. Occupational level was determined based on self-reported current occupation if still employed and/or last occupation before retirement or student. The reported occupations were further coded according to the International Standard Classification of Occupations scheme (ISCO-08**)**. Where 'high' (professionals, managers, clerical support staff, higher grade routine non-manual employees service and sales-related occupations) and 'low' (craft and related trades workers, elementary occupations and farmers) and the rest were categorize into the 'middle'. Wealth index was determined using the World Health Organization (WHO) standard of wealth index classification. Wealth index was based on data collected in the Household Questionnaire. The questionnaire comprised of questions on household's ownership of several consumer items such as television, car, flooring material, toilet facilities etc. Each household was assigned a standard score for each asset. Wealth index was then expressed in five categories. The five categories were further categorized into three categories by combining the second and third category due to small numbers [19]. All three SES constructs were further classified as low, medium and high SES and their relationship to each

For peer review only - http://bmjopen.bmj.com/site/about/guidelines.xhtml

other tested. A composite SES variable (SES) was generated based on the three SES constructs using the EGEN group command in STATA. This was also categorized into 3 categories (low, medium and high).

Co-morbidity factors

Blood pressure (BP) was measured three times using a validated semi-automated device (The Microlife WatchBP home) with appropriate cuffs in a sitting position after at least 5 min rest. The mean of the last two BP measurements was used in the analyses. Hypertension was defined as systolic BP ≥ 140 mmHg, and/or diastolic BP ≥ 90 mmHg, and/or being on antihypertensive medication treatment, and/or self-reported hypertension. Trained research assistants in the two sites collected fasting venous blood samples. All the blood samples were processed and aliquoted immediately (within one hour to maximum three hours of the vena puncture) after collection per standard operation procedures, and then temporarily stored at the local research location at −20°C. The separated samples were then transported to the local research centres laboratories, where they were checked, registered and stored at −80°C. To avoid intra-laboratory variability, the stored blood samples from the local research centres were transported to Berlin, Germany for biochemical analyses. Fasting plasma glucose concentration was measured using an enzymatic method (hexokinase). Type 2 diabetes was defined according to the WHO diagnostic criteria (fasting glucose ≥7.0 mmol/L, and/or current use of medication prescribed to treat diabetes, and/or self-reported diabetes) [20]. Concentration of total cholesterol was assessed using colorimetric test kits. All biochemical analyses were performed using an ABX Pentra 400 chemistry analyzer (ABX Pentra; Horiba ABX, Germany). Hypercholesterolemia was defined as total cholesterol level ≥ 6.22 mmol/L. Serum creatinine concentration (in umol/L) was determined by a kinetic colorimetric spectrophotometric isotope dilution mass spectrometry–calibrated method (Roche Diagnostics). Biochemical analyses were subject to extensive quality checks including blinded serial measurements.

Outcome: CKD prevalence

Participants were asked to bring an early morning urine sample for the analyses of albuminuria and creatinine levels. Urinary albumin concentration (in mg/L) was measured by an immunochemical turbidimetric method (Roche Diagnostics). Urinary creatinine concentration (in umol/L) was measured by a kinetic spectrophotometric method (Roche Diagnostics). Estimated glomerular filtration rate (eGFR) was calculated using the CKDEPI (CKD Epidemiology Collaboration) creatinine equation [21]. Urinary albumin-creatinine ratio (ACR; expressed in mg/g) was calculated by taking the ratio between urinary albumin and urinary creatinine. eGFR and albuminuria were categorized according to the 2012 KDIGO (Kidney Disease: Improving Global Outcomes) classification [22]. eGFR was categorized as follows: G1, ≥ 90 mL/min/1.73 m^2 (normal kidney function); G2, 60 to 89 mL/min/1.73 m^2 (mildly decreased); G3a, 45

For peer review only - http://bmjopen.bmj.com/site/about/guidelines.xhtml

to 59 mL/min/1.73 m^2 (mildly to moderately decreased); G3b, 30 to 44 mL/min/1.73 m^2 (moderately to severely decreased); G4, 15 to 29 mL/min/1.73 m^2 (severely decreased); and G5, < 15 mL/min/1.73 m^2 (kidney failure). Albuminuria categories were derived from ACR and were as follows: A1, < 3mg/mmol (normal to mildly increased); A2, 3 to 30 mg/mmol (moderately increased); and A3, > 30mg/mmol (severely increased). CKD status was categorized according to severity of kidney disease (green, low risk; yellow, moderately increased risk; orange, high risk; and red, very high risk) using the combination of eGFR (G1-G5) and albuminuria (A1-A3) levels defined by the 2012 KDIGO guideline [23]. Due to the small number of participants in the very high risk category of CKD, high and very high risk groups were combined. Reduced eGFR was defined as eGFR < 60 mL/min/1.73 m^2. Because of the small number of participants in the severely increased albuminuria category, we defined albuminuria as ACR ≥3 mg/mmol by combining the moderately increased (A2) and severely increased (A3) categories.

Patient and Public Involvement

Community leaders were involved in the recruitment of patients. These comprised of religious communities (churches and mosques), endorsement from local key leaders and establishing relationships with healthcare organizations. We also provided information on the study by involving the local media (radio and television stations). We sent letters to all selected health and community authorities to notify participants of the study. Team members were sent to the various community to stay among the community and organize mini clinics for a period of 1-2 weeks. Results of the study were disseminated through seminars, durbars and via radio and television stations.

Statistical methods

Participants' characteristics were expressed as absolute numbers and percentages for categorical variables and as means and standard deviations (SD) for continuous variables. CKD prevalence with 5% error bars were presented as bar graphs for each SES construct across rural and urban Ghana. Spearman's rank correlation was used to determine correlations between the three SES constructs. Odds ratios (ORs) and their corresponding 95% confidence intervals (CIs) were estimated by means of logistic regression analyses to study the odds of albuminuria (ACR>3 mg/mmol, A2-A3, moderately to severely increased albuminuria), reduced kidney function (eGFR< 60 mL/min/1.73 m^2, G3-G5 moderately to severely decreased kidney function) and increased CKD risk (high and very high CKD risk) by SES, with adjustments for potential covariates (age and sex). These covariates were adjusted for to account for their impact in the pathway of CKD incidence, prevalence and progression [24]. The analyses were performed for the total population (using low educational level, low occupational status and low level of wealth index as reference categories). Further analysis was conducted using rural Ghana as reference. Model 1

For peer review only - http://bmjopen.bmj.com/site/about/guidelines.xhtml

was adjusted for age and sex while model 2 was adjusted for age, sex and educational level. Model 3 was adjusted for age, sex and occupational status while model 4 was adjusted for age, sex and level of wealth index. Model 5 was adjusted for age, sex, educational level, occupational level and wealth index ($p<0.05$). Tolerance test and variance inflation factor (VIF) showed very small degree of collinearity among SES predictors thus we adjusted for each of SES variables separately. Complete case analysis approach was used. All data available were included in the age-adjusted models. All analyses were performed using STATA, version 14.0 (StataCorp LP).

Results

Table 1 shows characteristics of study participants. Participants in rural Ghana were slightly older than those in urban Ghana. Female preponderance was observed in both rural and urban Ghana, though higher proportions were observed in urban Ghana. Individuals living in rural Ghana were generally less educated compared with those living in urban Ghana. There were slightly more individuals with low occupational status in urban Ghana compared with their peers in rural Ghana. People in urban Ghana were wealthier than their rural counterparts. Rural Ghanaians were more physically active compared with their urban peers. Smoking was low among Ghanaians though rural Ghanaians were more likely to smoke compared with their urban peers. Hypercholesterolemia was more prevalent in urban Ghana than in rural Ghana. Hypertension and type 2 diabetes were more prevalent in urban Ghanaians compared with those living in rural Ghana. Urban Ghanaians were markedly more obese compared with their rural peers. Except for eGFR, albuminuria and CKD risk prevalence rates were higher in urban Ghana compared with rural Ghana.

Table 1: Baseline characteristics by location

	**Rural Ghana**	**Urban Ghana**
Number of participants, N (%)	1043 (41.9)	1449 (58.1)
Mean age, years (SD)	46.5 (12.6)	45.2 (11.4)
Females, N (%)	638 (61.2)	1034 (71.4)
Educational level n (%)		
Low	555 (56.9)	614 (43.9)
Middle	311 (31.9)	547 (39.1)
High	108 (11.2)	239 (17.0)
Occupational status, n (%)		
Low	250 (25.7)	374 (26.7)
Middle	628 (64.5)	818 (58.4)

For peer review only - http://bmjopen.bmj.com/site/about/guidelines.xhtml

High	96 (9.8)	209 (14.9)
Wealth index, n (%)		
Low	449 (46.5)	368 (26.6)
Middle	276 (28.6)	416 (30.0)
High	241 (24.9)	602 (43.4)
BMI (kg/m^2		
< 25	794 (76.3)	579 (39.9)
25-29.9	189 (18.2)	495 (34.2)
≥ 30	58 (5.5)	374 (25.9)
Low physical activity, n (%)	663 (47.22)	592 (60.7)
Smoking, n (%)	22 (2.3)	14 (1.0)
Hypercholesterolemia, n (%)	78 (7.6)	270 (18.7)
Hypertension, n (%)	306 (29.3)	531 (36.7)
Diabetes, n (%)	53 (5.1)	153 (10.6)
Albuminuria, n (%)		
A1, Normal to mildly increased (ACR <3 mg/mmol)	930 (91.6)	1285 (89.1)
A2-A3, moderately to severely increased (ACR≥ 3 mg/mmol)	85 (8.4)	158 (10.9)
eGFR, n (%)		
G1-G2 (≥ 60 mL/min/1.73m^2)	989 (96.3)	1388 (96.3)
G3-G5 (<60 mL/min/1.73m^2)	38 (3.7)	54 (3.7)
CKD risk, n (%)		
Low risk (green)	916 (90.5)	1281 (88.9)
Moderately increased to very high risk (yellow to red)	96 (9.5)	160 (11.1)

Abbreviations: N, number of respondents; SD, standard deviation; eGFR, estimated glomerular filtration rate; ACR, albumin creatinine ratio; CKD, Chronic kidney disease

Figure 1 shows prevalence of CKD by level of education in urban and rural Ghana. Prevalence of CKD decreased with increasing levels of education in rural Ghana. Higher prevalence of CKD was observed among individuals with low educational level compared with those with middle and high educational level. However, those with high educational level in urban Ghana had higher prevalence of CKD compared with those with middle level education. For occupational status, prevalence of CKD was higher among individuals with low occupational status in urban Ghana. Similar patterns were observed in rural Ghana, however, those with higher occupational status had higher prevalence of CKD compared with those with middle occupational status (Figure 2). Figure 3 shows prevalence of CKD by level of wealth index. CKD prevalence among the levels of wealth index varied between urban and rural Ghana. Those with middle level wealth index had higher prevalence of CKD compared with those with low or high CKD prevalence in both rural and urban Ghana. CKD prevalence rate for low and high level wealth index in urban Ghana was the same while that of rural Ghana was slightly different.

For peer review only

Among the whole group, educational level was positively associated with wealth index (p<0.01) and composite SES (P<0.01). Occupational level was also inversely associated with educational level (p<0.01) and wealth index (p<0.01). In urban Ghana, high educational level was positively associated with high wealth index but inversely associated with occupation (p<0.01). In rural Ghana, high education was positively associated with high wealth index (p<0.01), but there was no significant association between education and occupation. High wealth index was inversely associated with high occupational status in both rural and urban Ghana (p<0.01) (Table 2).

Table 2: Relationship between SES constructs (educational, occupational level and wealth index) by urban rural Ghana

Correlation matrix	Educational level	Occupational level	Wealth index	SES
**Whole group**				
Educational level	1.000			
Occupational status	**-0.060**	1.000		
	**0.004**			
Wealth Index	0.282	**-0.121**	1.000	
	0.001	**0.001**		
SES	**1.000**	**-0.059**	**0.282**	1.000
	**0.003**	**0.006**	**0.001**	
**Urban Ghana**				
Educational level	1.000			
Occupational status	**-0.115**	1.000		
	**0.001**			
Wealth Index	**0.294**	**-0.126**	1.000	

For peer review only

	**0.001**	**0.001**		
SES	**1.000**	**-0.024**	**0.937**	1.000
	**0.002**	**0.001**	**0.001**	
**Rural Ghana**				
Educational level	1.000			
Occupational status	0.017	1.000		
	0.589			
Wealth Index	**0.219**	**-0.135**	1.000	
	**0.001**	**0.001**		
SES	**0.504**	0.017	**0.934**	1.000
	**0.001**	0.587	**0.001**	

For peer review only

Table 3 shows association between level of education, occupational status, level of wealth index and prevalence of CKD. After adjusting for age and sex for the whole group, albuminuria was associated with middle level education ($p<0.01$). After adjusting for age and sex, we observed no significant association between SES indicators (educational level, occupational status and wealth index) and CKD in urban Ghana. However, middle and higher level education was associated with reduced albuminuria in urban Ghana ($p<0.01$). Whereas educational level and occupational status were not associated with CKD prevalence, high wealth index was significantly associated with higher odds of reduced eGFR ($p<0.01$).

Table 3: **Association of SES indicators (educational level, occupational status and wealth index level) with albuminuria, reduced eGFR and CKD risk**

	Albuminuria (ACR ≥ 3 mg/mmol)		eGFR < 60 mL/min/1.73 m2		High to very high CKD risk (KDIGO, 2012)	
		OR (95% CI)		OR (95% CI)		OR (95% CI)
	n (%)	Model 1	n (%)	Model 1	n cases (%)	Model 1
**Education**						
**Whole group**						
Low	1,152 (11.89)	1.00 (Reference)	1,160 (3.97)	1.00 (Reference)	1.150 (11.91)	1.00 (Reference)
Middle	847 (7.32)	**0.66 (0.48-0.91)**	849 (3.77)	1.36 (0.83-2.22)	845 (8.28)	0.82 (0.59-1.12)
High	343 (7.00)	0.67 (0.42-1.07)	345 (3.19)	1.11 (0.55-2.29)	343 (8.75)	0.96 (0.62-1.49)
**Urban Ghana**						
Low	612 (14.7)	1.00 (Reference)	612 (4.1)	1.00 (Reference)	612 (14.1)	1.00 (Reference)
Middle	546 (7.8)	**0.51 (0.34-0.76)**	546 (3.7)	1.12 (0.59-2.12)	545 (8.1)	**0.59 (0.39-0.89)**
High	238 (8.4)	**0.53 (0.31-0.91)**	238 (3.4)	0.91 (0.37-2.19)	238 (10.9)	0.83 (0.51-1.38)
**Rural Ghana**						
Low	540 (8.7)	1.00 (Reference)	548 (3.8)	1.00 (Reference)	538 (9.5)	1.00 (Reference)
Middle	301 (6.3)	0.89 (0.51-1.59)	303 (3.9)	1.69 (0.77-3.66)	300 (8.7)	1.33 (0.79-2.25)
High	105 (3.8)	0.66 (0.23-1.95)	107 (2.8)	1.28 (0.35-4.71)	105 (3.8)	0.69 (0.23-2.02)
**Occupational status**						
**Whole group**						
Low	614 (9.93)	1.00 (Reference)	616 (2.76)	1.00 (Reference)	613 (9.46)	1.00 (Reference)

For peer review only - http://bmjopen.bmj.com/site/about/guidelines.xhtml

For peer review only

Middle	1,427 (9.25)	0.82 (0.59-1.14)	1,436 (3.34)	0.93 (0.52-1.66)	1,424 (9.90)	0.89 (0.65-1.24)
High	302 (10.26)	0.76 (0.47-1.22)	303 (7.92)	1.33 (0.67-2.62)	302 (12.91)	0.90 (0.57-1.42)
**Urban Ghana**						
Low	207 (10.1)	1.00 (Reference)	207 (6.8)	1.00 (Reference)	207 (12.1)	1.00 (Reference)
Middle	817 (11.1)	1.50 (0.88-2.83)	817 (3.6)	1.15 (0.56-2.35)	816 (11.6)	1.37 (0.84-2.56)
High	373 (11.0)	1.57 (0.89-2.53)	373 (2.7)	1.02 (0.41-2.52)	373 (9.7)	1.21 (0.68-2.14)
**Rural Ghana**						
Low	95 (10.5)	1.00 (Reference)	96 (10.4)	1.00 (Reference)	95 (14.7)	1.00 (Reference)
Middle	610 (6.7)	0.65 (0.31-1.37)	619 (3.1)	**0.37 (0.16-0.85)**	608 (7.6)	0.55 (0.28-1.08)
High	241 (8.3)	0.99 (0.43-2.28)	243 (2.9)	0.51 (0.18-1.44)	240 (9.2)	0.94 (0.44-2.01)
**Wealth index**						
**Whole group**						
Low	808 (9.65)	1.00 (Reference)	813 (3.32)	1.00 (Reference)	808 (9.16)	1.00 (Reference)
Middle	678 (10.91)	1.18 (0.84-1.66)	683 (3.81)	1.30 (0.74-2.28)	675 (12.0)	1.43 (1.02-2.01)
High	835 (8.62)	0.93 (0.66-1.31)	835 (4.19)	1.55 (0.91-2.64)	833 (9.96)	1.21 (0.86-1.69)
**Urban Ghana**						
Low	367 (11.2)	1.00 (Reference)	367 (3.5)	1.00 (Reference)	367 (10.1)	1.00 (Reference)
Middle	414 (12.3)	1.12 (0.73-1.74)	414 (3.9)	1.30 (0.61-2.80)	413 (13.1)	1.45 (0.93-2.27)
High	601 (9.8)	0.82 (0.55-1.25)	600 (3.8)	1.13 (0.55-2.31)	600 (10.8)	1.11 (0.72-1.71)
**Rural Ghana**						
Low	441 (7.9)	1.00 (Reference)	446 (3.1)	1.00 (Reference)	441 (8.4)	1.00 (Reference)
Middle	264 (8.7)	1.13 (0.65-1.98)	269 (3.7)	1.22 (0.52-2.84)	262 (10.3)	1.31 (0.77-2.25)
High	234 (5.6)	0.78 (0.40-1.53)	235 (5.1)	**2.38 (1.03-5.47)**	233 (7.7)	1.16 (0.63-2.14)

Model 1, adjusted for age and sex; Abbreviations: CI, confidence interval; ACR, albumin creatinine ration; eGFR, estimated glomerular filtration rate; CKD, chronic kidney disease; OR, odds ratio, n= total number of individuals in the whole group, rural and urban Ghana among the various levels of SES constructs; %, proportion of individuals with CKD among the various levels of SES constructs in rural and urban Ghana.

For peer review only

Table 4 shows the contribution of all three SES constructs to rural and urban CKD prevalence differences. The odds of albuminuria and CKD risk was significantly higher in urban Ghana compared with rural Ghana. The higher rate of CKD observed in urban Ghana was not explained by the higher SES of that population as compared to their rural counterparts.

Table 4: Contribution of SES indicators to rural-urban differences in albuminuria, reduced eGFR and CKD risk

		OR (95% CI) Model 1	OR (95% CI) Model 2	OR (95% CI) Model 3	OR (95% CI) Model 4	OR (95% CI) Model 5
**Albuminuria (ACR ≥ 3 mg/mmol**						
Sites	n cases (%)					
Urban Ghana	1,443 (10.9)	**1.37 (1.03-1.81)**	**1.70 (1.25-2.31)**	**1.55 (1.15-2.10)**	**1.62 (1.18-2.19)**	**1.74 (1.27-2.38)**
Rural Ghana	1,015 (8.4)	1.00 (Reference)	1.00 (Reference)	1.00 (Reference)	1.00 (Reference)	1.00 (Reference)
**eGFR < 60 mL/min/1.73 m2**						
**Sites**	n cases (%)					
Urban Ghana	1,442 (3.7)	1.27 (0.82-1.97)	1.20 (0.76-1.89)	1.18 (0.79-1.86)	1.12 (0.70-1.78)	1.07 (0.67-1.72)
Rural Ghana	1,027 (3.7)	1.00 (Reference)	1.00 (Reference)	1.00 (Reference)	1.00 (Reference)	1.00 (Reference)
**High to very high CKD risk**						
**Sites**	n cases (%)					
Urban Ghana	1,441 (11.1)	**1.23 (1.01-1.62)**	**1.44 (1.07-1.93)**	**1.38 (1.03-1.84)**	**1.36 (1.01-1.83)**	**1.40 (1.04-1.91)**
Rural Ghana	1,012 (9.46)	1.00 (Reference)	1.00 (Reference)	1.00 (Reference)	1.00 (Reference)	1.00 (Reference

Model 1: adjusted for age and sex; Model 2: adjusted for age, sex and education level; Model 3: adjusted for age, sex and occupational status; Model 4: adjusted for age, sex and wealth index; Model 5: adjusted for age, sex, educational level, occupational status and wealth index; Abbreviations: CI, confidence interval; ACR, albumin creatinine ration; eGFR, estimated glomerular filtration rate; CKD, chronic kidney disease; OR, odds ratio, n=number of participants. n= total number of individuals in rural and urban Ghana; %, proportion of individuals with CKD among urban and rural Ghana.

For peer review only - http://bmjopen.bmj.com/site/about/guidelines.xhtml

Discussion

Key findings

Our study findings show no association between all three SES constructs and the prevalence of CKD in both rural and urban Ghana except for wealth index in rural Ghana, with the risk of CKD being higher in the wealthier populations. The higher rate of CKD observed in urban Ghana could not be attributed to the higher SES of that population compared to their rural counterparts.

Discussion of key findings

Association of SES with CKD in rural and urban Ghana

Our study did not find any significant associations between all three SES constructs and CKD among rural and urban Ghana except for wealth index in rural Ghana. The positive association observed between wealth index in rural Ghana may be due to a number of reasons. A comparison of the three SES constructs showed higher educational level to be associated with wealth index in both rural and urban Ghana but not occupational level. This seems to suggest that occupational level may not be adequately capturing the SES status of individuals living in these settings in relation to CKD. For example, Masthi et al, compared different SES scales in rural and urban India and concluded that Standard of Living Index (SLI) scale was more accurate for classification of SES in urban and rural setting [25]. Our finding is consistent with other studies, [6 26] which reported no association between SES and CKD in high-income countries and LMICs, but in contrast with other studies [2-4 27] that found positive associations between SES and CKD. The reasons for our current finding are unclear. However, it has been suggested that these inconsistent associations may be due to the varying pathways through which the effect of SES on health status is mediated. For example, at a given educational level marked ethnic differences have been reported. Additionally, similar differences were observed for wealth status at a given income level [28-30].

Contribution of SES to observed CKD risk differences between rural and urban Ghana

We observed higher rates of CKD in urban Ghana compared with rural Ghana, as expected. The observed higher rates of CKD in our study were not explained by the higher SES of that population as compared to their rural counterparts. Our results indicate that this is due to the lack of a clear difference in the SES distribution of rural and urban Ghana observed in this study, as well as to the lack of associations between SES and CKD. Consistent with our findings, in a study conducted in Northern Tanzania SES did not explain increased risk of CKD in urban Tanzania [26]. The lack of associations between SES and CKD could probably partly be explained by the process of epidemiological transition in relation to the

For peer review only - http://bmjopen.bmj.com/site/about/guidelines.xhtml

"diffusion theory" of ischemic heart disease mortality. This theory attributes the commencement of ischemic heart disease to individuals in the high SES group due to their ability to afford behaviours (smoking, alcohol and sedentary lifestyles) which increased risk of ischemic heart disease. The lower SES groups were later affected partially because of improved living standards, imitation and urbanization. The higher SES groups were the first to embrace behavioural changes required to decrease the risk of ischemic heart disease and this resulted in reversing the gradient [31]. The rapid urbanization of some rural communities in the Ashanti region of Ghana and the imitation of urban lifestyle could account for our finding. Also, it could be that whereas the high SES group in urban Ghana has already embraced favourable behavioural changes, those in rural Ghana are yet to do so [32]. This explains the observed association of wealth index with CKD in rural Ghana but not in urban Ghana. The complexities of influence of SES on prevalence and progression of CKD and the differential prevalence of established risk factors (diabetes, obesity and hypertension) in rural and urban Ghana may also contribute to the different associations of SES with CKD prevalence observed in rural and urban Ghana. In our study, the prevalence rates of hypercholesterolemia, hypertension and type 2 diabetes were substantially higher in urban Ghana compared with that of rural Ghana. Also, the interplay of other less understood or researched factors (e.g., exposure to nephrotoxins, herbal medications, sepsis) may be influencing the pathway in which SES influences CKD prevalence and progression.

Strength and limitation

Our study presents several strengths. First, we used well-standardized study protocols across rural and urban Ghana. Our study is also the first in Africa to use all three categories of CKD definition (albuminuria, reduced eGFR and CKD risk) by KDIGO 2012 in assessing association of SES with CKD in rural and urban setting, this provides a more detailed information on CKD outcomes. The limitation of intra laboratory variability in earlier studies was eliminated using the same standard operating procedures in the same laboratory for running all samples for both rural and urban Ghana. The use of three constructs of SES in this study also provides a much better holistic approach to assessing SES. Also, the distribution of SES in our study reflect on the national data allowing for generalization of our findings. Our study was limited by the use of cross sectional design, which prevented us from determining causality between predictors and CKD progression. Furthermore, there were more women than men in our study due to the higher response rate in women compared with men. However, this applied to both rural and urban Ghana. We therefore do not expect this to influence our results in a significant way.

Conclusion

For peer review only - http://bmjopen.bmj.com/site/about/guidelines.xhtml
For peer review only

All three SES constructs appear not to be associated with prevalence of CKD in urban and rural Ghana except for wealth index in rural Ghana. The observed higher prevalence of CKD in urban Ghana was not explained by the higher SES in urban Ghana. Our study seems to suggest that other non-traditional factors such as nephrotoxins, herbal medications and misuse of over the counter drugs may play a role and underscores the need to further explore these factors.

Acknowledgement

The authors are very grateful to the research assistants, interviewers and other staff of the five research locations who took part in gathering the data and the Ghanaian volunteers in all the participating RODAM sites. We gratefully acknowledge the advisory board members for their valuable support in shaping the RODAM study methods and the Academic Medical Centre Biobank for their support in biobank management and high-quality storage of collected samples.

Contributors

My co-authors have all contributed substantially to this manuscript and approve of this submission. Research idea and study design: DNA, CA, KS, DA, EB, KM, JA; data acquisition and curation: DNA, CA, EB, KM, data analysis/interpretation: DNA, CA, KS, DA, EB, KM, LS, JA, EOD, KKB, FPM, ID, JS, SB, ADA; statistical analysis: DNA, CA, KS. DNA, CA, KS, DA, EB, KM, LS, JA, EOD, KKB, FPM, ID, JS, SB, ADA contributed important intellectual content during manuscript drafting or revision and accepts accountability for the overall work by ensuring that questions pertaining to the accuracy or integrity of any portion of the work are appropriately investigated and resolved. DNA and CA takes responsibility that this study has been reported honestly, accurately, and transparently; that no important aspects of the study have been omitted; and that any discrepancies from the study as planned have been explained.

Funding

This work was supported by the European Commission under the Framework Programme (Grant Number: 278901). The funders had no role in study design, data collection and analysis, decision to publish, or preparation of the manuscript. The Wellcome Trust supported Professor Smeeth's contribution, grant number WT082178. Professor Joachim Spranger was supported by the DZHK (German Center for cardiovascular research) and the Berlin Institute of Health (BIH).

For peer review only - http://bmjopen.bmj.com/site/about/guidelines.xhtml

Competing interest: I have communicated with all my co-authors and obtained their full disclosures. My co-authors and I declare no conflicts of interest.

Patient Consent: None declared

Ethics approval: IRBs at each participating site.

Data sharing statement: Data are available from the RODAM research cohort, a third party. Dr. Eric Beune affiliated with the RODAM research cohort and a co-author of this paper in accordance with the RODAM requirements for collaboration. Dr. Beune is the Data Collection Coordinator of RODAM and may be contacted with further questions (e.j.beune@amc.uva.nl). Additionally, researchers interested in further collaboration with RODAM may see the following URL: http://www.rod-am.eu/

References

For peer review only - http://bmjopen.bmj.com/site/about/guidelines.xhtml

1. Patzer RE, McClellan WM. Influence of race, ethnicity and socioeconomic status on kidney disease. *Nature Reviews Nephrology* 2012;8(9):533-41.
2. Merkin SS, Roux AVD, Coresh J, et al. Individual and neighborhood socioeconomic status and progressive chronic kidney disease in an elderly population: The Cardiovascular Health Study. *Soc Sci Med* 2007;65(4):809-21.
3. Bello AK, Peters J, Rigby J, et al. Socioeconomic status and chronic kidney disease at presentation to a renal service in the United Kingdom. *Clin J Am Soc Nephrol* 2008;3(5):1316-23.
4. Crews DC, Charles RF, Evans MK, et al. Poverty, race, and CKD in a racially and socioeconomically diverse urban population. *Am J Kidney Dis* 2010;55(6):992-1000.
5. Shoham DA, Vupputuri S, Roux AVD, et al. Kidney disease in life-course socioeconomic context: the Atherosclerosis Risk in Communities (ARIC) Study. *Am J Kidney Dis* 2007;49(2):217-26.
6. Byrne C, Nedelman J, Luke RG. Race, socioeconomic status, and the development of end-stage renal disease. *Am J Kidney Dis* 1994;23(1):16-22.
7. Popkin BM. The shift in stages of the nutrition transition in the developing world differs from past experiences! *Public Health Nutr* 2002;5(1A):205-14.
8. Du S, Lu B, Zhai F, et al. A new stage of the nutrition transition in China. *Public Health Nutr* 2002;5(1a):169-74.
9. Kim S, Symons M, Popkin BM. Contrasting socioeconomic profiles related to healthier lifestyles in China and the United States. *Am J Epidemiol* 2004;159(2):184-91.
10. Adler NE, Ostrove JM. Socioeconomic status and health: what we know and what we don't. *Ann N Y Acad Sci* 1999;896(1):3-15.
11. Anderson NB, Armstead CA. Toward understanding the association of socioeconomic status and health: A new challenge for the biopsychosocial approach. *Psychosom Med* 1995;57(3):213-25.
12. Feinstein JS. The relationship between socioeconomic status and health: a review of the literature. *The Milbank Quarterly* 1993:279-322.
13. Samuel P, Antonisamy B, Raghupathy P, et al. Socio-economic status and cardiovascular risk factors in rural and urban areas of Vellore, Tamilnadu, South India. *Int J Epidemiol* 2012;41(5):1315-27.
14. Christie S, Fone DL. Does car ownership reflect socio-economic disadvantage in rural areas? A cross-sectional geographical study in Wales, UK. *Public Health* 2003;117(2):112-16.
15. Agyemang C, Beune E, Meeks K, et al. Rationale and cross-sectional study design of the Research on Obesity and type 2 Diabetes among African Migrants: the RODAM study. *BMJ open* 2015;4(3):e004877.
16. Addo J, Agyemang C, Aikins Ad-G, et al. Association between socioeconomic position and the prevalence of type 2 diabetes in Ghanaians in different geographic locations: the RODAM study. *J Epidemiol Community Health* 2017:jech-2016-208322.
17. Adjei DN, Stronks K, Adu D, et al. Relationship between educational and occupational levels, and Chronic Kidney Disease in a multi-ethnic sample-The HELIUS study. *PLoS One* 2017;12(11):e0186460.
18. Consultation WE. Waist circumference and waist-hip ratio. *Report of a WHO Expert Consultation Geneva: World Health Organization* 2008:8-11.
19. Rutstein SO, Johnson K, MEASURE OM. The DHS wealth index: ORC Macro, MEASURE DHS 2004.

For peer review only - http://bmjopen.bmj.com/site/about/guidelines.xhtml

20. Association AD. Standards of medical care in diabetes—2014. *Diabetes Care* 2014;37(Supplement 1):S14-S80.

21. Stevens LA, Schmid CH, Greene T, et al. Comparative performance of the CKD epidemiology collaboration (CKD-EPI) and the modification of diet in renal disease (MDRD) study equations for estimating GFR levels above 60 mL/min/1.73 m 2. *Am J Kidney Dis* 2010;56(3):486-95.

22. Eknoyan G, Lameire N, Eckardt K, et al. KDIGO 2012 clinical practice guideline for the evaluation and management of chronic kidney disease. *Kidney Int* 2013;3:5-14.

23. KDIGO G. Work Group. KDIGO clinical practice guideline for glomerulonephritis. *Kidney inter, Suppl* 2012;2:139-274.

24. Collins AJ, Foley RN, Herzog C, et al. US Renal Data System 2010 Annual Data Report. *American journal of kidney diseases: the official journal of the National Kidney Foundation* 2011;57(1 Suppl 1):A8, e1.

25. Masthi NR, Gangaboraiah PK. An exploratory study on socio economic status scales in a rural and urban setting. *Journal of family medicine and primary care* 2013;2(1):69.

26. Stanifer JW, Maro V, Egger J, et al. The epidemiology of chronic kidney disease in Northern Tanzania: a population-based survey. *PLoS One* 2015;10(4):e0124506.

27. Bruce MA, Beech BM, Crook ED, et al. Association of socioeconomic status and CKD among African Americans: the Jackson Heart Study. *Am J Kidney Dis* 2010;55(6):1001-08.

28. Braveman PA, Cubbin C, Egerter S, et al. Socioeconomic status in health research: one size does not fit all. *JAMA* 2005;294(22):2879-88.

29. Gilthorpe MS, Wilson RC. Rural/urban differences in the association between deprivation and healthcare utilisation. *Soc Sci Med* 2003;57(11):2055-63.

30. Choi AI, Weekley CC, Chen S-C, et al. Association of educational attainment with chronic disease and mortality: the Kidney Early Evaluation Program (KEEP). *Am J Kidney Dis* 2011;58(2):228-34.

31. Mackenbach JP, Cavelaars A, Kunst AE, et al. Socioeconomic inequalities in cardiovascular disease mortality. An international study. *Eur Heart J* 2000;21(14):1141-51.

32. McKay L, Macintyre S, Ellaway A. Migration and health: a review of the international literature. MRC Social and Public Health Sciences Unit, Occasional Paper No. 12. *Glasgow: Medical Research Council[Links]* 2003

For peer review only

Legend for figures

Figure 1: Prevalence of chronic kidney disease (CKD) across level of education among urban and rural participants Definitions according to 2012 KDIGO (Kidney Disease: Improving Global

For peer review only - http://bmjopen.bmj.com/site/about/guidelines.xhtml

Outcomes) guideline. CKD was defined as being in moderately increased risk, high-risk, or very high-risk groups.

Figure 2: Prevalence of chronic kidney disease (CKD) across occupational status among urban and rural participants. Definitions according to 2012 KDIGO (Kidney Disease: Improving Global Outcomes) guideline. CKD was defined as being in moderately increased risk, high-risk, or very high-risk groups.

Figure 3: Prevalence of chronic kidney disease (CKD) across wealth index categories among urban and rural participants. Definitions according to 2012 KDIGO (Kidney Disease: Improving Global Outcomes) guideline. CKD was defined as being in moderately increased risk, high-risk, or very high-risk groups.

For peer review only

For peer review only - http://bmjopen.bmj.com/site/about/guidelines.xhtml

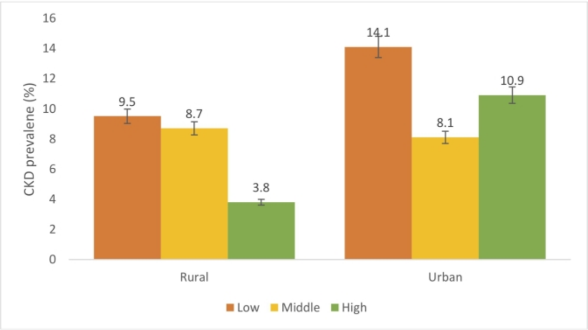

Figure 1: Prevalence of chronic kidney disease (CKD) across level of education among urban and rural participants Definitions according to 2012 KDIGO (Kidney Disease: Improving Global Outcomes) guideline. CKD was defined as being in moderately increased risk, high-risk, or very high-risk groups

Figure 1: Prevalence of chronic kidney disease (CKD) across level of education among urban and rural participants Definitions according to 2012 KDIGO (Kidney Disease: Improving Global Outcomes) guideline. CKD was defined as being in moderately increased risk, high-risk, or very high-risk groups.

279x361mm (300 x 300 DPI)

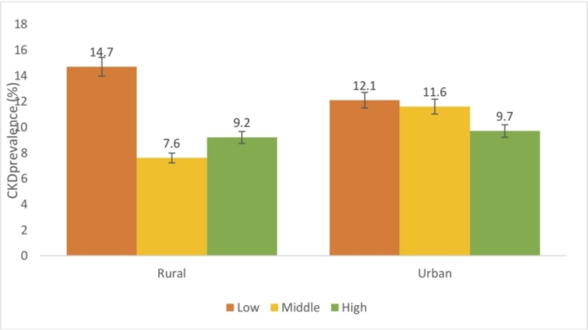

Figure 2: Prevalence of chronic kidney disease (CKD) across occupational status among urban and rural participants. Definitions according to 2012 KDIGO (Kidney Disease: Improving Global Outcomes) guideline. CKD was defined as being in moderately increased risk, high-risk, or very high-risk groups

Figure 2: Prevalence of chronic kidney disease (CKD) across occupational status among urban and rural participants. Definitions according to 2012 KDIGO (Kidney Disease: Improving Global Outcomes) guideline. CKD was defined as being in moderately increased risk, high-risk, or very high-risk groups.

279x361mm (300 x 300 DPI)

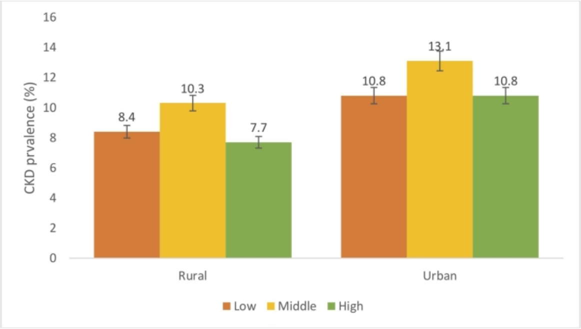

Figure 3: Prevalence of chronic kidney disease (CKD) across wealth index categories among urban and rural participants. Definitions according to 2012 KDIGO (Kidney Disease: Improving Global Outcomes) guideline. CKD was defined as being in moderately increased risk, high-risk, or very high-risk groups

Figure 3: Prevalence of chronic kidney disease (CKD) across wealth index categories among urban and rural participants. Definitions according to 2012 KDIGO (Kidney Disease: Improving Global Outcomes) guideline. CKD was defined as being in moderately increased risk, high-risk, or very high-risk groups.

279x361mm (300 x 300 DPI)

STROBE Statement—checklist of items that should be included in reports of observational studies

	Item No.	Recommendation	Page No.	Relevant text from manuscript
**Title and abstract**	1	(*a*) Indicate the study's design with a commonly used term in the title or the abstract	1,2	We have included a commonly used term in the title and abstract
		(*b*) Provide in the abstract an informative and balanced summary of what was done and what was found	2	Our study did not find any associations between SES indicators and CKD in both rural and urban Ghana after age and sex adjustment except in rural Ghana where wealth index was associated with prevalence of CKD. Consequently, the higher SES did not account for the increased rate of CKD among urban dwellers suggesting the need to identify other factors that may be driving this.
**Introduction**				
Background/rationale	2	Explain the scientific background and rationale for the investigation being reported	4	The theoretical and scientific background as well as the rationale for conducting the study have been provided in the introduction section.
Objectives	3	State specific objectives, including any prespecified hypotheses	4	We assessed the association of SES with CKD in rural and urban Ghana and studied what extent the higher SES of people in urban areas could account for differences in CKD between rural and urban populations
**Methods**				
Study design	4	Present key elements of study design early in the paper	5-6	Details given in the methods
Setting	5	Describe the setting, locations, and relevant dates, including periods of recruitment, exposure, follow-up, and data collection	5-6	Rural or urban Ghana.

For peer review only - http://bmjopen.bmj.com/site/about/guidelines.xhtml

Participants	6	(*a*) *Cohort study*—Give the eligibility criteria, and the sources and methods of selection of participants. Describe methods of follow-up<br>*Case-control study*—Give the eligibility criteria, and the sources and methods of case ascertainment and control selection. Give the rationale for the choice of cases and controls<br>*Cross-sectional study*—Give the eligibility criteria, and the sources and methods of selection of participants	6-8	A multi-centre cross-sectional study was conducted among Ghanaian adults (n=2492) aged 25-70 years residing in rural and urban Ghana.
		(*b*) *Cohort study*—For matched studies, give matching criteria and number of exposed and unexposed<br>*Case-control study*—For matched studies, give matching criteria and the number of controls per case		
Variables	7	Clearly define all outcomes, exposures, predictors, potential confounders, and effect modifiers. Give diagnostic criteria, if applicable	6-8	The main outcomes have been clearly defined.
Data sources/ measurement	8*	For each variable of interest, give sources of data and details of methods of assessment (measurement). Describe comparability of assessment methods if there is more than one group	*6-8*	We defined each variable of interest in the methods accordingly
Bias	9	Describe any efforts to address potential sources of bias	18	Potential sources of bias have discussed in the discussion section
Study size	10	Explain how the study size was arrived at	5	Given in the methods section and we have also referred to the RODAM study methods paper

Continued on next page

For peer review only - http://bmjopen.bmj.com/site/about/guidelines.xhtml

Quantitative variables	11	Explain how quantitative variables were handled in the analyses. If applicable, describe which groupings were chosen and why	8-9	Please see methods
Statistical methods	12	(*a*) Describe all statistical methods, including those used to control for confounding	8-9	Please see methods
		(*b*) Describe any methods used to examine subgroups and interactions	8-9	Please see methods
		(*c*) Explain how missing data were addressed	8-9	
		(*d*) *Cohort study*—If applicable, explain how loss to follow-up was addressed<br>*Case-control study*—If applicable, explain how matching of cases and controls was addressed<br>*Cross-sectional study*—If applicable, describe analytical methods taking account of sampling strategy	NA	We have reported non-response across sites
		(*e*) Describe any sensitivity analyses	NA	
**Results**				
Participants	13*	(a) Report numbers of individuals at each stage of study—eg numbers potentially eligible, examined for eligibility, confirmed eligible, included in the study, completing follow-up, and analysed	5	Non-response analysis was done to shed light on the differential response rates across sites
		(b) Give reasons for non-participation at each stage	5	
		(c) Consider use of a flow diagram	5	We have also referred to RODAM methods paper
Descriptive data	14*	(a) Give characteristics of study participants (eg demographic, clinical, social) and information on exposures and potential confounders	5	We have also referred to RODAM methods paper
		(b) Indicate number of participants with missing data for each variable of interest	5	We have also referred to RODAM methods paper
		(c) *Cohort study*—Summarise follow-up time (eg, average and total amount)	NA	
Outcome data	15*	*Cohort study*—Report numbers of outcome events or summary measures over time	*NA*	
		Case-control study—Report numbers in each exposure category, or summary measures of exposure	*NA*	
		Cross-sectional study—Report numbers of outcome events or summary measures	*9-10*	Summary measures are given in the results section and in tables and figures
Main results	16	(*a*) Give unadjusted estimates and, if applicable, confounder-adjusted estimates and their precision (eg, 95% confidence interval). Make clear which confounders were adjusted for and why they were included	*12-16*	Unadjusted and adjusted estimates are given in the results section and in figures
		(*b*) Report category boundaries when continuous variables were categorized	12-16	We have provided mean and corresponding standard deviations for the continuous variables.

For peer review only - http://bmjopen.bmj.com/site/about/guidelines.xhtml

		(*c*) If relevant, consider translating estimates of relative risk into absolute risk for a meaningful time	NA	

Continued on next page

Other analyses	17	Report other analyses done—eg analyses of subgroups and interactions, and sensitivity analyses	NA	
**Discussion**				
Key results	18	Summarise key results with reference to study objectives	8	
Limitations	19	Discuss limitations of the study, taking into account sources of potential bias or imprecision. Discuss both direction and magnitude of any potential bias	18	Key limitations regarding study methods including differential response rates and sampling methods in the various study sites have been provided
Interpretation	20	Give a cautious overall interpretation of results considering objectives, limitations, multiplicity of analyses, results from similar studies, and other relevant evidence	17-18	Cautious overall interpretation of the key findings have been provided.
Generalisability	21	Discuss the generalisability (external validity) of the study results	17-18	
**Other information**				
Funding	22	Give the source of funding and the role of the funders for the present study and, if applicable, for the original study on which the present article is based	19	The funders had no role in study design, data collection and analysis, decision to publish, or preparation of the manuscript

*Give information separately for cases and controls in case-control studies and, if applicable, for exposed and unexposed groups in cohort and cross-sectional studies.

Note: An Explanation and Elaboration article discusses each checklist item and gives methodological background and published examples of transparent reporting. The STROBE checklist is best used in conjunction with this article (freely available on the Web sites of PLoS Medicine at http://www.plosmedicine.org/, Annals of Internal Medicine at http://www.annals.org/, and Epidemiology at http://www.epidem.com/). Information on the STROBE Initiative is available at www.strobe-statement.org.

For peer review only - http://bmjopen.bmj.com/site/about/guidelines.xhtml

BMJ Open

A CROSS-SECTIONAL STUDY OF ASSOCIATION BETWEEN SOCIOECONOMIC INDICATORS AND CHRONIC KIDNEY DISEASE IN RURAL-URBAN GHANA-THE RODAM STUDY

Journal:	*BMJ Open*
Manuscript ID	bmjopen-2018-022610.R2
Article Type:	Research
Date Submitted by the Author:	05-Nov-2018
Complete List of Authors:	Adjei Nana, David ; University of Ghana, Department of Medical Laboratory Sciences; University of Amsterdam, Department of Public Health<br>Stronks, Karien; Academic Medical Center , Department of Public Health<br>Adu, Dwomoa; Korle-bu Teaching Hospital, Department of Medicine<br>Beune, Erik; AMC<br>Meeks, Karlijn; AMC, Public Health<br>Smeeth, Liam; London School of Hygiene and Tropical Medicine,<br>Addo, Juliet; London School of Hygiene and Tropical Medicine, Non Communicable Disease Epidemiology<br>Owusu-Dabo, Ellis; Kwame Nkrumah University of Science and Technology, Kumasi Centre for Collaborative Research in Tropical Medicine<br>Klipstein-Grobusch, Kerstin; 1 Julius Global Health, Julius Center for Health Sciences and Primary Care, University Medical Centre Utrecht, Utrecht, The Netherlands<br>Mockenhaupt, Frank; Charité – University Medicine, Berlin, Institute of Tropical Medicine and International Health<br>Danquah, Ina; German Institute of Human Nutrition, Molecular Epidemiology; Charite Universitatsmedizin Berlin, Institute for Social Medicine, Epidemiology and Health Economy<br>Spranger, Joachim; Department of Endocrinology and Metabolism, 1. Charité-University Medicine Berlin, Berlin, Germany.<br>Bahendeka, Silver; 1. MKPGMS - Uganda Martyrs University<br>De-Graft Aikins, Ama; University of Ghana, Regional Institute for Population Studies<br>Agyemang, Charles; Academic Medical centre, University of Amsterdam, Department of Public Health
**Primary Subject Heading**:	Epidemiology
Secondary Subject Heading:	Public health, Epidemiology
Keywords:	Chronic Kidney Disease, Socioeconomic status, Health inequalities, RODAM study, rural, urban

SCHOLARONE™
Manuscripts

A CROSS SECTIONAL STUDY OF ASSOCIATION BETWEEN SOCIOECONOMIC INDICATORS AND CHRONIC KIDNEY DISEASE IN RURAL-URBAN GHANA-THE RODAM STUDY

David N. Adjei, MSc, PhD[1,2]; Karien Stronks, MSc, PhD[1]; Dwomoa Adu, MD[3]; Erik Beune, MSc, PhD [1]; Karlijn Meeks, MSc, PhD [1]; Liam Smeeth, MD, PhD[4]; Juliet, Addo, MD, PhD [4]; Ellis Owuso-Dabo, MSc, PhD [5], Kerstin Klipstein-Grobusch, MSc, PhD [6,7]; Frank P. Mockenhaupt, MD, PhD [8]; Ina, Danquah, MSc, PhD [9,10]; Joachim, Spranger, MD, PhD [11,12,13]; Silver Bahendeka, MD, PhD[14]; Ama de-Graft, Aikins, MSc, PhD[15]; Charles Agyemang, MPH, PHD[1]

1. Department of Public Health, Academic Medical Center, University of Amsterdam, Amsterdam Public Health Research Institute, Amsterdam, The Netherlands.
2. Department of Medical Laboratory Sciences, School of Biomedical and Allied Health Sciences, College of Health Sciences, University of Ghana, Accra, Ghana.
3. Department of Medicine, School of Medicine and Dentistry, University of Ghana and Korle-Bu Teaching Hospital, Accra, Ghana.
4. Department of Non-Communicable Disease Epidemiology, London School of Hygiene and Tropical Medicine, London, United Kingdom.
5. Kumasi Centre for Collaborative Research, KNUST, Kumasi, Ghana
6. Julius Global Health, Julius Center for Health Sciences and Primary Care, University Medical Centre, Utrecht University, The Netherlands.
7. Division of Epidemiology & Biostatistics, School of Public Health, Faculty of Health Sciences, University of the Witwatersrand, Johannesburg, South Africa.
8. Institute of Tropical Medicine and International Health, Charité – University Medicine Berlin, Augustenburger Platz 1, 13353 Berlin, Germany.
9. Department of Molecular Epidemiology, German Institute of Human Nutrition Potsdam-Rehbrücke, Nuthetal, Germany.
10. Charité - Universitaetsmedizin Berlin, Institute for Social Medicine, Epidemiology and Health Economics, Berlin, Germany.
11. Department of Endocrinology and Metabolism, Charité-University Medicine Berlin, Berlin, Germany.
12. German Centre for Cardiovascular Research (DZHK), Berlin, Germany.
13. Center for Cardiovascular Research (CCR), Charité-University Medicine Berlin, Berlin, Germany.
14. MKPGMS - Uganda Martyrs University, Kampala, Uganda.
15. Regional Institute for Population Studies, University of Ghana, Legon, Ghana.

Address correspondence to David Nana Adjei, MSc, PhD Department of Public Health, Academic Medical Centre, University of Amsterdam, Meibergdreef 9, 1105 AZ Amsterdam, the

For peer review only

Netherlands, School of Biomedical and Allied Health Sciences, Medical Laboratory Sciences, University of Ghana, E-mail: dna@chs.edu.gh, d.n.adjei@amc.uva.nl, Tel: +233236717850

Abstract

Objectives: Studies from high income countries suggest higher prevalence of Chronic Kidney Disease (CKD) among individuals in low socio-economic groups. However, some studies from low and middle-income countries (LMICs) show the reverse pattern among those in high socioeconomic groups. It is unknown which pattern applies to individuals living in rural and urban Ghana. We therefore assessed the association between Socio-Economic Status (SES) indicators and CKD in rural and urban Ghana and to what extent the higher SES of people in urban areas of Ghana could account for differences in CKD between rural and urban populations.

Methods: We used baseline data from multi-centre Research on Obesity and Diabetes among African Migrants (RODAM) study. The sample consisted of 2492 adults (Rural Ghana, 1043, Urban Ghana, 1,449) aged 25 to 70 years living in Ghana. Three CKD outcomes were considered using the 2012 KDIGO (Kidney Disease: Improving Global Outcomes) severity of CKD classification: albuminuria (albumin-creatinine ratio ≥ 3 mg/mmol (category ≥ A2)); reduced glomerular filtration rate (eGFR < 60 mL/min/1.73 m2 (category ≥ G3)) and high to very high CKD risk based on the combination of these two.

Results: All three SES indicators were not associated with CKD in both rural and urban Ghana after age and sex adjustment except for rural Ghana where high wealth index was significantly associated with higher odds of reduced eGFR (AOR, 2.38; 95% C.I. 1.03-5.47). The higher rate of CKD observed in urban Ghana was not explained by the higher SES of that population.

Conclusion: SES indicators were not associated with prevalence of CKD except for wealth index and reduced eGFR in rural Ghana. Consequently, the higher SES of did not account for the increased rate of CKD among urban dwellers suggesting the need to identify other factors that may be driving this.

Index Words: Chronic kidney disease; socioeconomic status; health inequalities; risk factor; ethnic minority groups; migrants; RODAM study, Ghana

For peer review only - http://bmjopen.bmj.com/site/about/guidelines.xhtml

Strengths and limitation of the study

- The use of well-standardized study protocols across rural and urban Ghana eliminated intra protocol variability.

- Our study is also the first in Africa to use all three categories of CKD definitions (albuminuria, reduced eGFR and CKD risk) by KDIGO 2012 in assessing association of SES with CKD in rural and urban setting. This provides more detailed information on CKD outcomes.

- The limitation of intra laboratory variability in earlier studies was eliminated using the same standard operating procedures in the same laboratory for running all samples for both rural and urban Ghana.

- The use of three constructs of SES (educational level, occupational level and wealth index) in this study also provides a much better holistic approach to assessing SES associations with CKD.

- Our study was limited because of the use of cross sectional design which prevented us from determining causality between predictors and CKD progression.

For peer review only

Introduction

In general, individuals in lower socio-economic status (SES) groups have been shown to suffer more frequently from Chronic Kidney Disease (CKD), often progressing to End Stage Renal Disease (ESRD), and associated with inadequate dialysis treatment, reduced access to kidney transplantation and poor health outcomes [1]. Recent studies have consistently found low SES to be associated with higher risk of CKD among people of African origin [2-5].

However, in some settings the well-known inverse association between SES and CKD seems to be absent, or even reversed. For example, Bryne et al. did not find an association between SES and End Stage Renal Disease [6]. Other studies have found a positive association between SES and CKD [7 8]. Specifically, as SES improved, unhealthful lifestyle (unhealthy diet, physical inactivity, smoking and alcohol consumption) increased in China while that of the United States decreased with improved SES [9]. People with higher incomes, in these contexts, can afford a western

For peer review only - http://bmjopen.bmj.com/site/about/guidelines.xhtml

lifestyle, which is more readily available in the urban areas than in the rural areas. There is therefore an interaction between individual SES and environmental factors, such as food and sedentary life style in such populations [10-12]. Consequently, in those settings, people with a higher SES might have higher CKD risk.

In urban areas, the population in general has a higher SES than in rural areas [13]. For example, individuals with higher educational level migrate from rural areas to find higher occupations matching their higher education to improve on their wealth. If indeed a positive association between SES and CKD is observed in LMICs, this might underlie the well-known health differences between urban and rural areas, with urban areas having an increased risk of CKD [14]. So far, it is unknown whether the reversed SES gradient (higher risk in high SES group) might explain the higher burden of CKD in urban areas as compared to rural areas in Africa.

In view of this, we assessed the association of SES with CKD in rural and urban Ghana and studied what extent the higher SES of people in urban areas could account for differences in CKD between rural and urban populations.

For peer review only

Methods

Study population and study design

In the present analyses, data from the RODAM (**R**esearch on **O**besity & **D**iabetes among **A**frican **M**igrants) study, a multi-centre cross-sectional study were used. The rationale, conceptual framework, design and methodology of the RODAM study have been described in detail elsewhere [15 16]. As the Healthy Life in an Urban Setting

For peer review only - http://bmjopen.bmj.com/site/about/guidelines.xhtml

(HELIUS) study conducted among Ghanaian migrants living in Amsterdam did not find any associations between SES and CKD [17] the current study focused on rural and urban Ghana (Ashanti region of Ghana). The RODAM study was conducted from 2012 to 2015 and it comprised of individuals aged 25-70 years living in rural and urban Ghana and Ghanaian migrants in Europe. All participants below 25 and above 70 years were excluded in the present analyses. The present analysis was restricted to the rural and urban sites (n=2492) RODAM participants. Specifically, 1043 participants from rural Ghana and 1449 from urban Ghana were used in this study.

Data collection for the study was standardized across all sites. Written informed consent was obtained from each participant prior to enrolment in the study. The respective ethics committees in Ghana and the three European countries approved the study protocols before data collection began. Specifically, we obtained ethical clearance in Ghana from School of Medical Sciences/Komfo Anokye Teaching Hospital Committee on Human Research, Publication & Ethical Review Board. In the Netherlands the Institutional Review Board of the AMC, University of Amsterdam gave approval for the study. In Germany, approval for the study was obtained from the Ethics Committee of Charite-Universitäts medizin. The London School of Hygiene and Tropical Medicine Research Ethics Committee gave approval for the study in the UK.

The response rate was 76% in rural Ghana and 74% in urban Ghana. In Ghana, participants were randomly drawn from a list of 30 enumeration areas in the Ashanti region based on the 2010 population census using the multistage random sampling. These enumeration areas came from two purposively selected urban cities (Kumasi and Obuasi) and 15 randomly selected rural communities in the Ashanti region.

For peer review only - http://bmjopen.bmj.com/site/about/guidelines.xhtml

Selected health and community authorities were first identified, notified of the study and letters were sent giving detailed explanation of the study. We sent team members to stay among the communities to familiarize with them and organize mini clinics in the field. This lasted between 1-2 weeks depending on the sampled population and responsiveness of respondents.

In Ghana, questionnaires administration and physical examination were done at the same day/time. The participants were instructed to fast from 10.00 pm the night before the physical examination. For the current study, 2566 participants with data available on both questionnaire data and physical measurements were used. We excluded (n=74) individuals outside the RODAM age range of 25-70 years resulting in a data set of 2492 for analysis. These comprised 1,449 Urban Ghana and 1043 Rural Ghana. For the final analysis, individuals with no data on CKD status (n=42) were excluded.

Measurements

Covariates

Demographic and lifestyle factors

Information on demographics, educational level, occupational level, wealth index and lifestyle factors (smoking and physical activity) were obtained by questionnaire. Physical examinations were performed with validated devices per standardized operational procedures across all study sites. Weight was measured in light clothing and without shoes with SECA 877 scales to the nearest 0.1 kg. Height was measured without shoes with a portable stadiometer (SECA 217) to the nearest 0.1 cm. Body mass index (BMI) was calculated as weight (kg) divided by height squared (m^2). Overweight was defined as BMI of ≥25 to <30 kg/m^2 and obesity as BMI ≥30 kg/m^2 [18]. Per participant, all anthropometrics were measured twice by the same assessor and the average of the two measurements were used for analyses.

Predictor: SES

For peer review only - http://bmjopen.bmj.com/site/about/guidelines.xhtml

Socioeconomic indicators used in this study were educational level, occupational status and level of wealth index. Educational level was determined based on self-reported highest educational qualification accomplished based on the Ghanaian educational system. Occupational level was determined based on self-reported current occupation if still employed and/or last occupation before retirement or student. The reported occupations were further coded according to the International Standard Classification of Occupations scheme (ISCO-08**).** Where 'high' (professionals, managers, clerical support staff, higher grade routine non-manual employees service and sales-related occupations) and 'low' (craft and related trades workers, elementary occupations and farmers) and the rest were categorized into the 'middle'. Wealth index was determined using the World Health Organization (WHO) standard of wealth index classification. Wealth index was based on data collected in the Household Questionnaire. The questionnaire comprised of questions on household's ownership of several consumer items such as television, car, flooring material, toilet facilities etc. Each household was assigned a standard score for each asset. Wealth index was then expressed in five categories. The five categories were further categorized into three categories by combining the second and third category due to small numbers [19]. All three SES constructs were further classified as low, medium and high SES and their relationship to each other tested. A composite SES variable (SES) was generated based on the three SES constructs (education, occupation and wealth index) using the EGEN group command in STATA. The codes were combined into numerical variables and their averages computed. The resultant values were recoded into three categories (low, medium and high).

Co-morbidity factors

Blood pressure (BP) was measured three times using a validated semi-automated device (The Microlife WatchBP home) with appropriate cuffs in a sitting position after at least 5 min rest. The mean of the last two BP measurements was used in the analyses. Hypertension was defined as systolic BP ≥ 140 mmHg, and/or diastolic BP ≥ 90 mmHg, and/or being on antihypertensive medication treatment, and/or self-reported hypertension. Trained research assistants in the two sites collected fasting venous blood samples. All the blood samples were processed and aliquoted immediately (within one hour to maximum three hours of the vena puncture) after collection per standard operation procedures, and then temporarily stored at the local research location at −20°C. The separated samples were then transported to the local research centres laboratories, where they were checked, registered and stored at −80°C. To avoid intra-laboratory variability, the stored blood samples from the local research centres were transported to Berlin, Germany for biochemical analyses. Fasting plasma glucose concentration was measured using an enzymatic method (hexokinase). Type 2 diabetes was defined according to the WHO diagnostic criteria (fasting glucose ≥7.0 mmol/L, and/or current use of medication prescribed to treat diabetes, and/or self-

For peer review only - http://bmjopen.bmj.com/site/about/guidelines.xhtml

reported diabetes) [20]. Concentration of total cholesterol was assessed using colorimetric test kits. All biochemical analyses were performed using an ABX Pentra 400 chemistry analyzer (ABX Pentra; Horiba ABX, Germany). Hypercholesterolemia was defined as total cholesterol level ≥ 6.22 mmol/L. Serum creatinine concentration (in umol/L) was determined by a kinetic colorimetric spectrophotometric isotope dilution mass spectrometry–calibrated method (Roche Diagnostics). Biochemical analyses were subject to extensive quality checks including blinded serial measurements.

Outcome: CKD prevalence

Participants were asked to bring an early morning urine sample for the analyses of albuminuria and creatinine levels. Urinary albumin concentration (in mg/L) was measured by an immunochemical turbidimetric method (Roche Diagnostics). Urinary creatinine concentration (in umol/L) was measured by a kinetic spectrophotometric method (Roche Diagnostics). Estimated glomerular filtration rate (eGFR) was calculated using the CKDEPI (CKD Epidemiology Collaboration) creatinine equation [21]. Urinary albumin-creatinine ratio (ACR; expressed in mg/g) was calculated by taking the ratio between urinary albumin and urinary creatinine. eGFR and albuminuria were categorized according to the 2012 KDIGO (Kidney Disease: Improving Global Outcomes) classification [22]. eGFR was categorized as follows: G1, ≥ 90 mL/min/1.73 m^2 (normal kidney function); G2, 60 to 89 mL/min/1.73 m^2 (mildly decreased); G3a, 45 to 59 mL/min/1.73 m^2 (mildly to moderately decreased); G3b, 30 to 44 mL/min/1.73 m^2 (moderately to severely decreased); G4, 15 to 29 mL/min/1.73 m^2 (severely decreased); and G5, < 15 mL/min/1.73 m^2 (kidney failure). Albuminuria categories were derived from ACR and were as follows: A1, < 3mg/mmol (normal to mildly increased); A2, 3 to 30 mg/mmol (moderately increased); and A3, > 30mg/mmol (severely increased). CKD status was categorized according to severity of kidney disease (green, low risk; yellow, moderately increased risk; orange, high risk; and red, very high risk) using the combination of eGFR (G1-G5) and albuminuria (A1-A3) levels defined by the 2012 KDIGO guideline [23]. Due to the small number of participants in the very high risk category of CKD, high and very high risk groups were combined. Reduced eGFR was defined as eGFR < 60 mL/min/1.73 m^2. Because of the small number of participants in the severely increased albuminuria category, we defined albuminuria as ACR ≥3 mg/mmol by combining the moderately increased (A2) and severely increased (A3) categories.

Patient and Public Involvement

Community leaders were involved in the recruitment of patients. These comprised of religious communities (churches and mosques), endorsement from local key leaders and establishing relationships with healthcare organizations. We also provided information on the study by involving the local media (radio and television stations). We sent letters to all selected health and community authorities to notify

For peer review only

For peer review only - http://bmjopen.bmj.com/site/about/guidelines.xhtml

participants of the study. Team members were sent to the various communities to stay among the community and organize mini clinics for a period of 1-2 weeks. Results of the study were disseminated through seminars, durbars and via radio and television stations.

Statistical methods

Participants' characteristics were expressed as absolute numbers and percentages for categorical variables and as means and standard deviations (SD) for continuous variables. CKD prevalence with 5% error bars were presented as bar graphs for each SES construct across rural and urban Ghana. Spearman's rank correlation was used to determine correlations between the three SES constructs. Odds ratios (ORs) and their corresponding 95% confidence intervals (CIs) were estimated by means of logistic regression analyses to study the odds of albuminuria (ACR>3 mg/mmol, A2-A3, moderately to severely increased albuminuria), reduced kidney function (eGFR< 60 mL/min/1.73 m^2, G3-G5 moderately to severely decreased kidney function) and increased CKD risk (high and very high CKD risk) by SES, with adjustments for potential confounders (age and sex).[24] In addition, the analyses were performed for the total population (using low educational level, low occupational status and low level of wealth index as reference categories). Further analysis was conducted to assess the contribution of SES indicators to rural-urban differences in albuminuria, reduced eGFR and CKD risk using rural Ghana as reference. Tolerance test and variance inflation factor (VIF) showed very small degree of collinearity among SES predictors thus we therefore adjusted for each of SES variables separately. Complete case analysis approach was used. All data available were included in the age-adjusted models. All analyses were performed using STATA, version 14.0 (StataCorp LP).

Results

Table 1 shows characteristics of study participants. Participants in rural Ghana were slightly older than those in urban Ghana. Female preponderance was observed in both rural and urban Ghana, though higher proportions were observed in urban Ghana. Individuals living in rural Ghana were generally less educated compared with those

For peer review only - http://bmjopen.bmj.com/site/about/guidelines.xhtml

living in urban Ghana. There were slightly more individuals with low occupational status in urban Ghana compared with their peers in rural Ghana. People in urban Ghana were wealthier than their rural counterparts. Rural Ghanaians were more physically active compared with their urban peers. Smoking was low among Ghanaians though rural Ghanaians were more likely to smoke compared with their urban peers. Hypercholesterolemia was more prevalent in urban Ghana than in rural Ghana. Hypertension and type 2 diabetes were more prevalent in urban Ghanaians compared with those living in rural Ghana. Urban Ghanaians were markedly more obese compared with their rural peers. Except for eGFR, albuminuria and CKD risk prevalence rates were higher in urban Ghana compared with rural Ghana.

Table 1: Baseline characteristics by location

	Rural Ghana	Urban Ghana
Number of participants, N (%)	1043 (41.9)	1449 (58.1)
Mean age, years (SD)	46.5 (12.6)	45.2 (11.4)
Females, N (%)	638 (61.2)	1034 (71.4)
Educational level n (%)		
Low	555 (56.9)	614 (43.9)
Middle	311 (31.9)	547 (39.1)
High	108 (11.2)	239 (17.0)
Occupational status, n (%)		
Low	250 (25.7)	374 (26.7)
Middle	628 (64.5)	818 (58.4)
High	96 (9.8)	209 (14.9)
Wealth index, n (%)		
Low	449 (46.5)	368 (26.6)
Middle	276 (28.6)	416 (30.0)

For peer review only

For peer review only - http://bmjopen.bmj.com/site/about/guidelines.xhtml

High	241 (24.9)	602 (43.4)
BMI (kg/m^2		
< 25	794 (76.3)	579 (39.9)
25-29.9	189 (18.2)	495 (34.2)
≥ 30	58 (5.5)	374 (25.9)
Low physical activity, n (%)	663 (47.22)	592 (60.7)
Smoking, n (%)	22 (2.3)	14 (1.0)
Hypercholesterolemia, n (%)	78 (7.6)	270 (18.7)
Hypertension, n (%)	306 (29.3)	531 (36.7)
	53	
Diabetes, n (%)	(5.1)	153 (10.6)
Albuminuria, n (%)		
	930 (91.6)	
A1, Normal to mildly increased (ACR <3 mg/mmol)	85	1285 (89.1)
A2-A3, moderately to severely increased (ACR≥ 3 mg/	(8.4)	158 (10.9)
eGFR, n (%)		
G1-G2 (≥ 60 mL/min/1.73m^2)	989 (96.3)	1388 (96.3)
G3-G5 (<60 mL/min/1.73m^2)	38 (3.7)	54 (3.7)
CKD risk, n (%)		
Low risk (green)	916 (90.5)	1281 (88.9)
Moderately increased to very high risk (yellow to red)	96 (9.5)	160 (11.1)

Abbreviations: N, number of respondents; SD, standard deviation; eGFR, estimated glomerular filtration rate; ACR, albumin creatinine ratio; CKD, Chronic kidney disease

Figure 1 shows prevalence of CKD by level of education in urban and rural Ghana. Prevalence of CKD decreased with increasing levels of education in rural Ghana. Higher prevalence of CKD was observed among individuals with low educational level compared with those with middle and high educational level. However, those with high educational level in urban Ghana had higher prevalence of CKD compared with those

For peer review only

For peer review only - http://bmjopen.bmj.com/site/about/guidelines.xhtml

329 with middle level education. For occupational status, prevalence of CKD was higher
330 among individuals with low occupational status in urban Ghana. Similar patterns were
331 observed in rural Ghana; however, those with higher occupational status had higher
332 prevalence of CKD compared with those with middle occupational status (Figure 2).
333 Figure 3 shows prevalence of CKD by level of wealth index. CKD prevalence among
334 the levels of wealth index varied between urban and rural Ghana. Those with middle
335 level wealth index had higher prevalence of CKD compared with those with low or
336 high CKD prevalence in both rural and urban Ghana. CKD prevalence rate for low and
337 high level wealth index in urban Ghana was the same while that of rural Ghana was
338 slightly different.

For peer review only

Among the whole group, educational level was positively associated with wealth index ($p<0.01$) and composite SES ($P<0.01$). Occupational level was also inversely associated with educational level ($p<0.01$) and wealth index ($p<0.01$). In urban Ghana, high educational level was positively associated with high wealth index but inversely associated with occupation ($p<0.01$). In rural Ghana, high education was positively associated with high wealth index ($p<0.01$), but there was no significant association between education and occupation. High wealth index was inversely associated with high occupational status in both rural and urban Ghana ($p<0.01$) (Table 2).

Table 2: Relationship between SES constructs (educational, occupational level and wealth index) by urban rural Ghana

Correlation matrix	Educational level	Occupational level	Wealth index	SES
**Whole group**				
Educational level	1.000			
Occupational status	**-0.060**	1.000		
	**0.004**			
Wealth Index	0.282	**-0.121**	1.000	
	0.001	**0.001**		
SES	**1.000**	**-0.059**	**0.282**	1.000
	**0.003**	**0.006**	**0.001**	

For peer review only

**Urban Ghana**				
Educational level	1.000			
Occupational status	**-0.115**	1.000		
	**0.001**			
Wealth Index	**0.294**	**-0.126**	1.000	
	**0.001**	**0.001**		
SES	**1.000**	**-0.024**	**0.937**	1.000
	**0.002**	**0.001**	**0.001**	
**Rural Ghana**				
Educational level	1.000			
Occupational status	0.017	1.000		
	0.589			
Wealth Index	**0.219**	**-0.135**	1.000	
	**0.001**	**0.001**		
SES	**0.504**	0.017	**0.934**	1.000
	**0.001**	0.587	**0.001**	

For peer review only

Table 3 shows association between level of education, occupational status, level of wealth index and prevalence of CKD. After adjusting for age and sex for the whole group, albuminuria was associated with middle level education ($p<0.01$). After adjusting for age and sex, we observed no significant association between SES indicators (educational level, occupational status and wealth index) and CKD in urban Ghana. However, middle and higher level education was associated with reduced albuminuria in urban Ghana ($p<0.01$). Whereas educational level and occupational status were not associated with CKD prevalence, high wealth index was significantly associated with higher odds of reduced eGFR ($p<0.01$).

Table 3: Association of SES indicators (educational level, occupational status and wealth index level) with albuminuria, reduced eGFR and CKD risk

	Albuminuria (ACR ≥ 3 mg/mmol)		eGFR < 60 mL/min/1.73 m2		High to very high CKD risk (KDIGO, 2012)	
		OR (95% CI)		OR (95% CI)		OR (95% CI)
	n (%)	Model 1	n (%)	Model 1	n (%)	Model 1

For peer review only - http://bmjopen.bmj.com/site/about/guidelines.xhtml

**Education**						
**Whole group**						
Low	1,152 (11.89)	1.00 (Reference)	1,160 (3.97)	1.00 (Reference)	1.150 (11.91)	1.00 (Reference)
Middle	847 (7.32)	**0.66 (0.48-0.91)**	849 (3.77)	1.36 (0.83-2.22)	845 (8.28)	0.82 (0.59-1.12)
High	343 (7.00)	0.67 (0.42-1.07)	345 (3.19)	1.11 (0.55-2.29)	343 (8.75)	0.96 (0.62-1.49)
**Urban Ghana**						
Low	612 (14.7)	1.00 (Reference)	612 (4.1)	1.00 (Reference)	612 (14.1)	1.00 (Reference)
Middle	546 (7.8)	**0.51 (0.34-0.76)**	546 (3.7)	1.12 (0.59-2.12)	545 (8.1)	**0.59 (0.39-0.89)**
High	238 (8.4)	**0.53 (0.31-0.91)**	238 (3.4)	0.91 (0.37-2.19)	238 (10.9)	0.83 (0.51-1.38)
**Rural Ghana**						
Low	540 (8.7)	1.00 (Reference)	548 (3.8)	1.00 (Reference)	538 (9.5)	1.00 (Reference)
Middle	301 (6.3)	0.89 (0.51-1.59)	303 (3.9)	1.69 (0.77-3.66)	300 (8.7)	1.33 (0.79-2.25)
High	105 (3.8)	0.66 (0.23-1.95)	107 (2.8)	1.28 (0.35-4.71)	105 (3.8)	0.69 (0.23-2.02)
**Occupational status**						
**Whole group**						
Low	614 (9.93)	1.00 (Reference)	616 (2.76)	1.00 (Reference)	613 (9.46)	1.00 (Reference)
Middle	1,427 (9.25)	0.82 (0.59-1.14)	1,436 (3.34)	0.93 (0.52-1.66)	1,424 (9.90)	0.89 (0.65-1.24)
High	302 (10.26)	0.76 (0.47-1.22)	303 (7.92)	1.33 (0.67-2.62)	302 (12.91)	0.90 (0.57-1.42)
**Urban Ghana**						
Low	207 (10.1)	1.00 (Reference)	207 (6.8)	1.00 (Reference)	207 (12.1)	1.00 (Reference)
Middle	817 (11.1)	1.50 (0.88-2.83)	817 (3.6)	1.15 (0.56-2.35)	816 (11.6)	1.37 (0.84-2.56)
High	373 (11.0)	1.57 (0.89-2.53)	373 (2.7)	1.02 (0.41-2.52)	373 (9.7)	1.21 (0.68-2.14)
**Rural Ghana**						
Low	95 (10.5)	1.00 (Reference)	96 (10.4)	1.00 (Reference)	95 (14.7)	1.00 (Reference)
Middle	610 (6.7)	0.65 (0.31-1.37)	619 (3.1)	**0.37 (0.16-0.85)**	608 (7.6)	0.55 (0.28-1.08)
High	241 (8.3)	0.99 (0.43-2.28)	243 (2.9)	0.51 (0.18-1.44)	240 (9.2)	0.94 (0.44-2.01)
**Wealth index**						
**Whole group**						

For peer review only

Low	808 (9.65)	1.00 (Reference)	813 (3.32)	1.00 (Reference)	808 (9.16)	1.00 (Reference)
Middle	678 (10.91)	1.18 (0.84-1.66)	683 (3.81)	1.30 (0.74-2.28)	675 (12.0)	1.43 (1.02-2.01)
High	835 (8.62)	0.93 (0.66-1.31)	835 (4.19)	1.55 (0.91-2.64)	833 (9.96)	1.21 (0.86-1.69)
**Urban Ghana**						
Low	367 (11.2)	1.00 (Reference)	367 (3.5)	1.00 (Reference)	367 (10.1)	1.00 (Reference)
Middle	414 (12.3)	1.12 (0.73-1.74)	414 (3.9)	1.30 (0.61-2.80)	413 (13.1)	1.45 (0.93-2.27)
High	601 (9.8)	0.82 (0.55-1.25)	600 (3.8)	1.13 (0.55-2.31)	600 (10.8)	1.11 (0.72-1.71)
**Rural Ghana**						
Low	441 (7.9)	1.00 (Reference)	446 (3.1)	1.00 (Reference)	441 (8.4)	1.00 (Reference)
Middle	264 (8.7)	1.13 (0.65-1.98)	269 (3.7)	1.22 (0.52-2.84)	262 (10.3)	1.31 (0.77-2.25)
High	234 (5.6)	0.78 (0.40-1.53)	235 (5.1)	**2.38 (1.03-5.47)**	233 (7.7)	1.16 (0.63-2.14)

Model 1, adjusted for age and sex; Abbreviations: CI, confidence interval; ACR, albumin creatinine ration; eGFR, estimated glomerular filtration rate; CKD, chronic kidney disease; OR, odds ratio, n= total number of individuals in the whole group, rural and urban Ghana among the various levels of SES constructs; %, proportion of individuals with CKD among the various levels of SES constructs in rural and urban Ghana.

Table 4 shows the contribution of all three SES constructs to rural and urban CKD prevalence differences. The odds of albuminuria and CKD risk was significantly higher in urban Ghana compared with rural Ghana. The higher rate of CKD observed in urban Ghana was not explained by the higher SES of that population as compared to their rural counterparts.

Table 4: Contribution of SES indicators to rural-urban differences in albuminuria, reduced eGFR and CKD risk

	**OR (95% CI)**	**OR (95% CI)**	**OR (95% CI)**	**OR (95% CI)**	**OR (95% CI)**

For peer review only - http://bmjopen.bmj.com/site/about/guidelines.xhtml

		Model 1	Model 2	Model 3	Model 4	Model 5
**Albuminuria (ACR ≥ 3 mg/mmol**						
Sites	n cases (%)					
Urban Ghana	1,443 (10.9)	**1.37 (1.03-1.81)**	**1.70 (1.25-2.31)**	**1.55 (1.15-2.10**)	**1.62 (1.18-2.19)**	**1.74 (1.27-2.38)**
Rural Ghana	1,015 (8.4)	1.00 (Reference)	1.00 (Reference)	1.00 (Reference**)**	1.00 (Reference**)**	1.00 (Reference**)**
**eGFR < 60 mL/min/1.73 m2**						
**Sites**	n cases (%)					
Urban Ghana	1,442 (3.7)	1.27 (0.82-1.97)	1.20 (0.76-1.89)	1.18 (0.79-1.86)	1.12 (0.70-1.78)	1.07 (0.67-1.72)
Rural Ghana	1,027 (3.7)	1.00 (Reference)	1.00 (Reference)	1.00 (Reference)	1.00 (Reference)	1.00 (Reference**)**
**High to very high CKD risk**						
**Sites**	n cases (%)					
Urban Ghana	1,441 (11.1)	**1.23 (1.01-1.62)**	**1.44 (1.07-1.93)**	**1.38 (1.03-1.84)**	**1.36 (1.01-1.83)**	**1.40 (1.04-1.91)**
Rural Ghana	1,012 (9.46)	1.00 (Reference)	1.00 (Reference)	1.00 (Reference)	1.00 (Reference**)**	1.00 (Reference

For peer review only

Model 1#: adjusted for age and sex; Model 2: adjusted for age, sex and education level; Model 3: adjusted for age, sex and occupational status; Model 4: adjusted for age, sex and wealth index; Model 5: adjusted for age, sex, educational level, occupational status and wealth index; Abbreviations: CI, confidence interval; ACR, albumin creatinine ratio; eGFR, estimated glomerular filtration rate; CKD, chronic kidney disease; OR, odds ratio, n= total number of individuals in rural and urban Ghana; %, proportion of individuals with CKD among urban and rural Ghana.

For peer review only

Discussion

Key findings

Our study findings show no association between all three SES constructs and the prevalence of CKD in both rural and urban Ghana except for wealth index in rural Ghana, with the risk of CKD being higher in the wealthier populations. The higher rate of CKD observed in urban Ghana could not be attributed to the higher SES of that population compared to their rural counterparts.

Discussion of key findings

Association of SES with CKD in rural and urban Ghana

Our study did not find any significant associations between all three SES constructs and CKD among rural and urban Ghana except for wealth index in rural Ghana. The positive association observed between wealth index in rural Ghana may be due to several reasons. A comparison of the three SES constructs showed higher educational level to be associated with wealth index in both rural and urban Ghana but not occupational level. This seems to suggest that occupational level may not be adequately capturing the SES status of individuals living in these settings in relation to CKD. For example, Masthi et al, compared different SES scales in rural and urban India and concluded that Standard of Living Index (SLI) scale was more accurate for classification of SES in urban and rural settings [25]. Our finding is consistent with other studies, [6 26] which reported no association between SES and CKD in high-income countries and LMICs, but in contrast with other studies [2-4 27] that found positive associations between SES

For peer review only - http://bmjopen.bmj.com/site/about/guidelines.xhtml

and CKD. The reasons for our current finding are unclear. However, it has been suggested that these inconsistent associations may be due to the varying pathways through which the effect of SES on health status is mediated. For example, at a given educational level marked ethnic differences have been reported. Additionally, similar differences were observed for wealth status at a given income level [28-30].

Contribution of SES to observed CKD risk differences between rural and urban Ghana

We observed higher rates of CKD in urban Ghana compared with rural Ghana, as expected. The observed higher rates of CKD in our study were not explained by the higher SES of that population as compared to their rural counterparts. Our results indicate that this is due to the lack of a clear difference in the SES distribution of rural and urban Ghana observed in this study, as well as to the lack of associations between SES and CKD. Consistent with our findings, in a study conducted in Northern Tanzania SES did not explain increased risk of CKD in urban Tanzania [26]. The lack of associations between SES and CKD could probably partly be explained by the process of epidemiological transition in relation to the "diffusion theory" of ischemic heart disease mortality. This theory attributes the commencement of ischemic heart disease to individuals in the high SES group due to their ability to afford behaviours (smoking, alcohol and sedentary lifestyles) which increased risk of ischemic heart disease. The lower SES groups were later affected partially because of improved living standards, imitation and urbanization. The higher SES groups were the first to embrace behavioural changes required to decrease the risk of ischemic heart disease and this resulted in reversing the gradient [31]. The rapid urbanization of some rural communities in the Ashanti region of Ghana and the imitation of urban lifestyle could account for our finding. Also, it could be that whereas the high SES group in urban Ghana has already embraced favourable behavioural changes, those in rural Ghana are yet to do so [32]. This explains the observed association of wealth index with CKD in rural Ghana but not in urban Ghana. Also, the interplay of other less understood or researched factors

For peer review only - http://bmjopen.bmj.com/site/about/guidelines.xhtml

(e.g., exposure to nephrotoxins, herbal medications, sepsis, psychosocial factors) may be influencing the pathway in which SES influences CKD prevalence and progression.

Strength and limitation

Our study presents several strengths. First, we used well-standardized study protocols across rural and urban Ghana. Our study is also the first in Africa to use all three categories of CKD definition (albuminuria, reduced eGFR and CKD risk) by KDIGO 2012 in assessing association of SES with CKD in rural and urban setting, this provided more detailed information on CKD outcomes. The limitation of intra laboratory variability in earlier studies was eliminated using the same standard operating procedures in the same laboratory for running all samples for both rural and urban Ghana. The use of three constructs of SES in this study also provides a much better holistic approach to assessing SES. Also, the distribution of SES in our study reflects on the national data allowing for generalization of our findings. Our study was limited by the use of cross sectional design, which prevented us from determining causality between predictors and CKD progression. Furthermore, there were more women than men in our study due to the higher response rate in women compared with men. However, this applied to both rural and urban Ghana. We therefore do not expect this to influence our results in a significant way.

Conclusion

All three SES constructs appear not to be associated with prevalence of CKD in urban and rural Ghana except for wealth index in rural Ghana. The observed higher prevalence of CKD in urban Ghana was not explained by the higher SES in urban Ghana. Our study seems to suggest that other non-traditional factors such as nephrotoxins, herbal medications, psychosocial stressors and misuse of over the counter drugs may play a role and underscores the need to further explore these factors.

For peer review only - http://bmjopen.bmj.com/site/about/guidelines.xhtml

Acknowledgement

The authors are very grateful to the research assistants, interviewers and other staff of the five research locations who took part in gathering the data and the Ghanaian volunteers in all the participating RODAM sites. We gratefully acknowledge the advisory board members for their valuable support in shaping the RODAM study methods and the Academic Medical Centre Biobank for their support in biobank management and high-quality storage of collected samples.

Contributors

My co-authors have all contributed substantially to this manuscript and approve of this submission. Research idea and study design: DNA, CA, KS, DA, EB, KM, JA; data acquisition and curation: DNA, CA, EB, KM, data analysis/interpretation: DNA, CA, KS, DA, EB, KM, LS, JA, EOD, KKG, FPM, ID, JS, SB, ADA; statistical analysis: DNA, CA, KS. DNA, CA, KS, DA, EB, KM, LS, JA, EOD, KKG, FPM, ID, JS, SB, ADA contributed important intellectual content during manuscript drafting or revision and accepts accountability for the overall work by ensuring that questions pertaining to the accuracy or integrity of any portion of the work are appropriately investigated and resolved. DNA and CA take responsibility that this study has been reported honestly, accurately, and transparently; that no important aspects of the study have been omitted; and that any discrepancies from the study as planned have been explained.

Funding

This work was supported by the European Commission under the Framework Programme (Grant Number: 278901). The funders had no role in study design, data collection and analysis, decision to publish, or preparation of the manuscript. The Wellcome Trust supported Professor Smeeth's contribution, grant number WT082178. Professor Joachim Spranger was supported by the DZHK (German Center for cardiovascular research) and the Berlin Institute of Health (BIH).

Competing interest: I have communicated with all my co-authors and obtained their full disclosures. My co-authors and I declare no conflicts of interest.

For peer review only - http://bmjopen.bmj.com/site/about/guidelines.xhtml

Patient Consent: None declared

Ethics approval: IRBs at each participating site.

Data sharing statement: Data are available from the RODAM research cohort, a third party. Dr. Eric Beune affiliated with the RODAM research cohort and a co-author of this paper in accordance with the RODAM requirements for collaboration. Dr. Beune is the Data Collection Coordinator of RODAM and may be contacted with further questions (e.j.beune@amc.uva.nl). Additionally, researchers interested in further collaboration with RODAM may see the following URL: http://www.rod-am.eu/

For peer review only

References

1. Patzer RE, McClellan WM. Influence of race, ethnicity and socioeconomic status on kidney disease. *Nature Reviews Nephrology* 2012;8(9):533-41.
2. Merkin SS, Roux AVD, Coresh J, et al. Individual and neighborhood socioeconomic status and progressive chronic kidney disease in an elderly population: The Cardiovascular Health Study. *Soc Sci Med* 2007;65(4):809-21.
3. Bello AK, Peters J, Rigby J, et al. Socioeconomic status and chronic kidney disease at presentation to a renal service in the United Kingdom. *Clin J Am Soc Nephrol* 2008;3(5):1316-23.
4. Crews DC, Charles RF, Evans MK, et al. Poverty, race, and CKD in a racially and socioeconomically diverse urban population. *Am J Kidney Dis* 2010;55(6):992-1000.
5. Shoham DA, Vupputuri S, Roux AVD, et al. Kidney disease in life-course socioeconomic context: the Atherosclerosis Risk in Communities (ARIC) Study. *Am J Kidney Dis* 2007;49(2):217-26.
6. Byrne C, Nedelman J, Luke RG. Race, socioeconomic status, and the development of end-stage renal disease. *Am J Kidney Dis* 1994;23(1):16-22.
7. Popkin BM. The shift in stages of the nutrition transition in the developing world differs from past experiences! *Public Health Nutr* 2002;5(1A):205-14.
8. Du S, Lu B, Zhai F, et al. A new stage of the nutrition transition in China. *Public Health Nutr* 2002;5(1a):169-74.
9. Kim S, Symons M, Popkin BM. Contrasting socioeconomic profiles related to healthier lifestyles in China and the United States. *Am J Epidemiol* 2004;159(2):184-91.
10. Adler NE, Ostrove JM. Socioeconomic status and health: what we know and what we don't. *Ann N Y Acad Sci* 1999;896(1):3-15.
11. Anderson NB, Armstead CA. Toward understanding the association of socioeconomic status and health: A new challenge for the biopsychosocial approach. *Psychosom Med* 1995;57(3):213-25.
12. Feinstein JS. The relationship between socioeconomic status and health: a review of the literature. *The Milbank Quarterly* 1993:279-322.

For peer review only - http://bmjopen.bmj.com/site/about/guidelines.xhtml

13. Samuel P, Antonisamy B, Raghupathy P, et al. Socio-economic status and cardiovascular risk factors in rural and urban areas of Vellore, Tamilnadu, South India. *Int J Epidemiol* 2012;41(5):1315-27.
14. Christie S, Fone DL. Does car ownership reflect socio-economic disadvantage in rural areas? A cross-sectional geographical study in Wales, UK. *Public Health* 2003;117(2):112-16.
15. Agyemang C, Beune E, Meeks K, et al. Rationale and cross-sectional study design of the Research on Obesity and type 2 Diabetes among African Migrants: the RODAM study. *BMJ open* 2015;4(3):e004877.
16. Addo J, Agyemang C, Aikins Ad-G, et al. Association between socioeconomic position and the prevalence of type 2 diabetes in Ghanaians in different geographic locations: the RODAM study. *J Epidemiol Community Health* 2017:jech-2016-208322.
17. Adjei DN, Stronks K, Adu D, et al. Relationship between educational and occupational levels, and Chronic Kidney Disease in a multi-ethnic sample-The HELIUS study. *PLoS One* 2017;12(11):e0186460.
18. Consultation WE. Waist circumference and waist-hip ratio. *Report of a WHO Expert Consultation Geneva: World Health Organization* 2008:8-11.
19. Rutstein SO, Johnson K, MEASURE OM. The DHS wealth index: ORC Macro, MEASURE DHS 2004.
20. Association AD. Standards of medical care in diabetes—2014. *Diabetes Care* 2014;37(Supplement 1):S14-S80.
21. Stevens LA, Schmid CH, Greene T, et al. Comparative performance of the CKD epidemiology collaboration (CKD-EPI) and the modification of diet in renal disease (MDRD) study equations for estimating GFR levels above 60 mL/min/1.73 m 2. *Am J Kidney Dis* 2010;56(3):486-95.
22. Eknoyan G, Lameire N, Eckardt K, et al. KDIGO 2012 clinical practice guideline for the evaluation and management of chronic kidney disease. *Kidney Int* 2013;3:5-14.
23. KDIGO G. Work Group. KDIGO clinical practice guideline for glomerulonephritis. *Kidney inter, Suppl* 2012;2:139-274.
24. Collins AJ, Foley RN, Herzog C, et al. US Renal Data System 2010 Annual Data Report. *American journal of kidney diseases: the official journal of the National Kidney Foundation* 2011;57(1 Suppl 1):A8, e1.
25. Masthi NR, Gangaboraiah PK. An exploratory study on socio economic status scales in a rural and urban setting. *Journal of family medicine and primary care* 2013;2(1):69.
26. Stanifer JW, Maro V, Egger J, et al. The epidemiology of chronic kidney disease in Northern Tanzania: a population-based survey. *PLoS One* 2015;10(4):e0124506.
27. Bruce MA, Beech BM, Crook ED, et al. Association of socioeconomic status and CKD among African Americans: the Jackson Heart Study. *Am J Kidney Dis* 2010;55(6):1001-08.
28. Braveman PA, Cubbin C, Egerter S, et al. Socioeconomic status in health research: one size does not fit all. *JAMA* 2005;294(22):2879-88.
29. Gilthorpe MS, Wilson RC. Rural/urban differences in the association between deprivation and healthcare utilisation. *Soc Sci Med* 2003;57(11):2055-63.
30. Choi AI, Weekley CC, Chen S-C, et al. Association of educational attainment with chronic disease and mortality: the Kidney Early Evaluation Program (KEEP). *Am J Kidney Dis* 2011;58(2):228-34.

For peer review only - http://bmjopen.bmj.com/site/about/guidelines.xhtml

31. Mackenbach JP, Cavelaars A, Kunst AE, et al. Socioeconomic inequalities in cardiovascular disease mortality. An international study. *Eur Heart J* 2000;21(14):1141-51.
32. McKay L, Macintyre S, Ellaway A. Migration and health: a review of the international literature. MRC Social and Public Health Sciences Unit, Occasional Paper No. 12. *Glasgow: Medical Research Council[Links]* 2003

Legend for figures

Figure 1: Prevalence of chronic kidney disease (CKD) across level of education among urban and rural participants Definitions according to 2012 KDIGO (Kidney Disease: Improving Global Outcomes) guideline. CKD was defined as being in moderately increased risk, high-risk, or very high-risk groups.

Figure 2: Prevalence of chronic kidney disease (CKD) across occupational status among urban and rural participants. Definitions according to 2012 KDIGO (Kidney Disease: Improving Global Outcomes) guideline. CKD was defined as being in moderately increased risk, high-risk, or very high-risk groups.

Figure 3: Prevalence of chronic kidney disease (CKD) across wealth index categories among urban and rural participants. Definitions according to 2012 KDIGO (Kidney Disease: Improving Global Outcomes) guideline. CKD was defined as being in moderately increased risk, high-risk, or very high-risk groups.

For peer review only

For peer review only - http://bmjopen.bmj.com/site/about/guidelines.xhtml

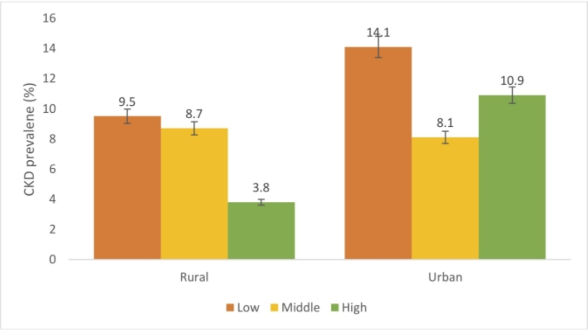

Figure 1: Prevalence of chronic kidney disease (CKD) across level of education among urban and rural participants Definitions according to 2012 KDIGO (Kidney Disease: Improving Global Outcomes) guideline. CKD was defined as being in moderately increased risk, high-risk, or very high-risk groups

Figure 1: Prevalence of chronic kidney disease (CKD) across level of education among urban and rural participants Definitions according to 2012 KDIGO (Kidney Disease: Improving Global Outcomes) guideline. CKD was defined as being in moderately increased risk, high-risk, or very high-risk groups.

279x361mm (300 x 300 DPI)

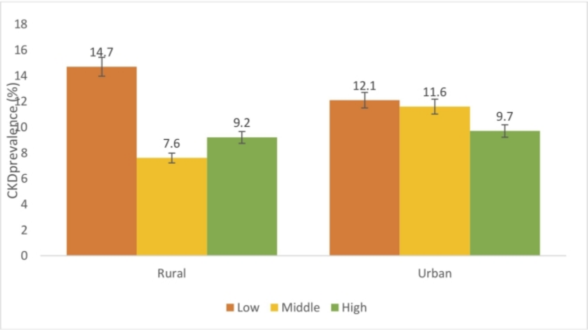

Figure 2: Prevalence of chronic kidney disease (CKD) across occupational status among urban and rural participants. Definitions according to 2012 KDIGO (Kidney Disease: Improving Global Outcomes) guideline. CKD was defined as being in moderately increased risk, high-risk, or very high-risk groups

Figure 2: Prevalence of chronic kidney disease (CKD) across occupational status among urban and rural participants. Definitions according to 2012 KDIGO (Kidney Disease: Improving Global Outcomes) guideline. CKD was defined as being in moderately increased risk, high-risk, or very high-risk groups.

279x361mm (300 x 300 DPI)

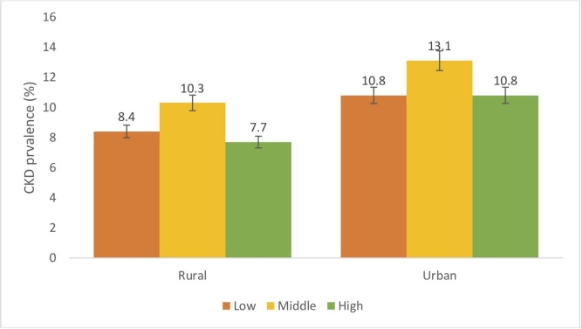

Figure 3: Prevalence of chronic kidney disease (CKD) across wealth index categories among urban and rural participants. Definitions according to 2012 KDIGO (Kidney Disease: Improving Global Outcomes) guideline. CKD was defined as being in moderately increased risk, high-risk, or very high-risk groups

Figure 3: Prevalence of chronic kidney disease (CKD) across wealth index categories among urban and rural participants. Definitions according to 2012 KDIGO (Kidney Disease: Improving Global Outcomes) guideline. CKD was defined as being in moderately increased risk, high-risk, or very high-risk groups.

279x361mm (300 x 300 DPI)

STROBE Statement—checklist of items that should be included in reports of observational studies

	Item No.	Recommendation	Page No.	Relevant text from manuscript
**Title and abstract**	1	(*a*) Indicate the study's design with a commonly used term in the title or the abstract	1,2	We have included a commonly used term in the title and abstract
		(*b*) Provide in the abstract an informative and balanced summary of what was done and what was found	2	Our study did not find any associations between SES indicators and CKD in both rural and urban Ghana after age and sex adjustment except in rural Ghana where wealth index was associated with prevalence of CKD. Consequently, the higher SES did not account for the increased rate of CKD among urban dwellers suggesting the need to identify other factors that may be driving this.
**Introduction**				
Background/rationale	2	Explain the scientific background and rationale for the investigation being reported	4	The theoretical and scientific background as well as the rationale for conducting the study have been provided in the introduction section.
Objectives	3	State specific objectives, including any prespecified hypotheses	4	We assessed the association of SES with CKD in rural and urban Ghana and studied what extent the higher SES of people in urban areas could account for differences in CKD between rural and urban populations
**Methods**				
Study design	4	Present key elements of study design early in the paper	5-6	Details given in the methods
Setting	5	Describe the setting, locations, and relevant dates, including periods of recruitment, exposure, follow-up, and data collection	5-6	Rural or urban Ghana.

For peer review only - http://bmjopen.bmj.com/site/about/guidelines.xhtml

For peer review only

Participants	6	(*a*) *Cohort study*—Give the eligibility criteria, and the sources and methods of selection of participants. Describe methods of follow-up<br>*Case-control study*—Give the eligibility criteria, and the sources and methods of case ascertainment and control selection. Give the rationale for the choice of cases and controls<br>*Cross-sectional study*—Give the eligibility criteria, and the sources and methods of selection of participants	6-8	A multi-centre cross-sectional study was conducted among Ghanaian adults (n=2492) aged 25-70 years residing in rural and urban Ghana.
		(*b*) *Cohort study*—For matched studies, give matching criteria and number of exposed and unexposed<br>*Case-control study*—For matched studies, give matching criteria and the number of controls per case		
Variables	7	Clearly define all outcomes, exposures, predictors, potential confounders, and effect modifiers. Give diagnostic criteria, if applicable	6-8	The main outcomes have been clearly defined.
Data sources/ measurement	8*	For each variable of interest, give sources of data and details of methods of assessment (measurement). Describe comparability of assessment methods if there is more than one group	*6-8*	We defined each variable of interest in the methods accordingly
Bias	9	Describe any efforts to address potential sources of bias	18	Potential sources of bias have discussed in the discussion section
Study size	10	Explain how the study size was arrived at	5	Given in the methods section and we have also referred to the RODAM study methods paper

Continued on next page

For peer review only - http://bmjopen.bmj.com/site/about/guidelines.xhtml

Quantitative variables	11	Explain how quantitative variables were handled in the analyses. If applicable, describe which groupings were chosen and why	8-9	Please see methods
Statistical methods	12	(*a*) Describe all statistical methods, including those used to control for confounding	8-9	Please see methods
		(*b*) Describe any methods used to examine subgroups and interactions	8-9	Please see methods
		(*c*) Explain how missing data were addressed	8-9	
		(*d*) *Cohort study*—If applicable, explain how loss to follow-up was addressed<br>*Case-control study*—If applicable, explain how matching of cases and controls was addressed<br>*Cross-sectional study*—If applicable, describe analytical methods taking account of sampling strategy	NA	We have reported non-response across sites
		(*e*) Describe any sensitivity analyses	NA	
**Results**				
Participants	13*	(a) Report numbers of individuals at each stage of study—eg numbers potentially eligible, examined for eligibility, confirmed eligible, included in the study, completing follow-up, and analysed	5	Non-response analysis was done to shed light on the differential response rates across sites
		(b) Give reasons for non-participation at each stage	5	
		(c) Consider use of a flow diagram	5	We have also referred to RODAM methods paper
Descriptive data	14*	(a) Give characteristics of study participants (eg demographic, clinical, social) and information on exposures and potential confounders	5	We have also referred to RODAM methods paper
		(b) Indicate number of participants with missing data for each variable of interest	5	We have also referred to RODAM methods paper
		(c) *Cohort study*—Summarise follow-up time (eg, average and total amount)	NA	
Outcome data	15*	*Cohort study*—Report numbers of outcome events or summary measures over time	*NA*	
		Case-control study—Report numbers in each exposure category, or summary measures of exposure	*NA*	
		Cross-sectional study—Report numbers of outcome events or summary measures	*9-10*	Summary measures are given in the results section and in tables and figures
Main results	16	(*a*) Give unadjusted estimates and, if applicable, confounder-adjusted estimates and their precision (eg, 95% confidence interval). Make clear which confounders were adjusted for and why they were included	*12-16*	Unadjusted and adjusted estimates are given in the results section and in figures
		(*b*) Report category boundaries when continuous variables were categorized	12-16	We have provided mean and corresponding standard deviations for the continuous variables.

For peer review only - http://bmjopen.bmj.com/site/about/guidelines.xhtml

For peer review only

		(*c*) If relevant, consider translating estimates of relative risk into absolute risk for a meaningful time	NA	

Continued on next page

Other analyses	17	Report other analyses done—eg analyses of subgroups and interactions, and sensitivity analyses	NA	
**Discussion**				
Key results	18	Summarise key results with reference to study objectives	8	
Limitations	19	Discuss limitations of the study, taking into account sources of potential bias or imprecision. Discuss both direction and magnitude of any potential bias	18	Key limitations regarding study methods including differential response rates and sampling methods in the various study sites have been provided
Interpretation	20	Give a cautious overall interpretation of results considering objectives, limitations, multiplicity of analyses, results from similar studies, and other relevant evidence	17-18	Cautious overall interpretation of the key findings have been provided.
Generalisability	21	Discuss the generalisability (external validity) of the study results	17-18	
**Other information**				
Funding	22	Give the source of funding and the role of the funders for the present study and, if applicable, for the original study on which the present article is based	19	The funders had no role in study design, data collection and analysis, decision to publish, or preparation of the manuscript

*Give information separately for cases and controls in case-control studies and, if applicable, for exposed and unexposed groups in cohort and cross-sectional studies.

Note: An Explanation and Elaboration article discusses each checklist item and gives methodological background and published examples of transparent reporting. The STROBE checklist is best used in conjunction with this article (freely available on the Web sites of PLoS Medicine at http://www.plosmedicine.org/, Annals of Internal Medicine at http://www.annals.org/, and Epidemiology at http://www.epidem.com/). Information on the STROBE Initiative is available at www.strobe-statement.org.

For peer review only - http://bmjopen.bmj.com/site/about/guidelines.xhtml

BMJ Open

A CROSS-SECTIONAL STUDY OF ASSOCIATION BETWEEN SOCIOECONOMIC INDICATORS AND CHRONIC KIDNEY DISEASE IN RURAL-URBAN GHANA: THE RODAM STUDY

Journal:	*BMJ Open*
Manuscript ID	bmjopen-2018-022610.R3
Article Type:	Research
Date Submitted by the Author:	15-Feb-2019

Complete List of Authors:	Adjei Nana, David ; University of Ghana, Department of Medical Laboratory Sciences; University of Amsterdam, Department of Public Health<br>Stronks, Karien; Academic Medical Center , Department of Public Health<br>Adu, Dwomoa; Korle-bu Teaching Hospital, Department of Medicine<br>Beune, Erik; AMC<br>Meeks, Karlijn; AMC, Public Health<br>Smeeth, Liam; London School of Hygiene and Tropical Medicine,<br>Addo, Juliet; London School of Hygiene and Tropical Medicine, Non Communicable Disease Epidemiology<br>Owusu-Dabo, Ellis; Kwame Nkrumah University of Science and Technology, Kumasi Centre for Collaborative Research in Tropical Medicine<br>Klipstein-Grobusch, Kerstin; 1 Julius Global Health, Julius Center for Health Sciences and Primary Care, University Medical Centre Utrecht, Utrecht, The Netherlands<br>Mockenhaupt, Frank; Charité – University Medicine, Berlin, Institute of Tropical Medicine and International Health<br>Danquah, Ina; German Institute of Human Nutrition, Molecular Epidemiology; Charite Universitatsmedizin Berlin, Institute for Social Medicine, Epidemiology and Health Economy<br>Spranger, Joachim; Department of Endocrinology and Metabolism, 1. Charité-University Medicine Berlin, Berlin, Germany.<br>Bahendeka, Silver; 1. MKPGMS - Uganda Martyrs University<br>De-Graft Aikins, Ama; University of Ghana, Regional Institute for Population Studies<br>Agyemang, Charles; Academic Medical centre, University of Amsterdam, Department of Public Health

**Primary Subject Heading**:	Epidemiology
Secondary Subject Heading:	Public health, Epidemiology
Keywords:	Chronic Kidney Disease, Socioeconomic status, Health inequalities, RODAM study, rural, urban

For peer review only - http://bmjopen.bmj.com/site/about/guidelines.xhtml

A CROSS-SECTIONAL STUDY OF ASSOCIATION BETWEEN SOCIOECONOMIC INDICATORS AND CHRONIC KIDNEY DISEASE IN RURAL-URBAN GHANA: THE RODAM STUDY

David N. Adjei, MSc, PhD[1,2]; Karien Stronks, MSc, PhD[1]; Dwomoa Adu, MD[3]; Erik Beune, MSc, PhD[1]; Karlijn Meeks, MSc, PhD [1]; Liam Smeeth, MD, PhD[4]; Juliet, Addo, MD, PhD[4]; Ellis Owusu-Dabo, MSc, PhD[5], Kerstin Klipstein-Grobusch, MSc, PhD[6,7]; Frank P. Mockenhaupt, MD, PhD[8]; Ina, Danquah, MSc, PhD [9,10]; Joachim, Spranger, MD, PhD[11,12,13]; Silver Bahendeka, MD, PhD[14]; Ama de-Graft, Aikins, MSc, PhD[15]; Charles Agyemang, MPH, PHD[1]

1. Department of Public Health, Academic Medical Center, University of Amsterdam, Amsterdam Public Health Research Institute, Amsterdam, The Netherlands.
2. Department of Medical Laboratory Sciences, School of Biomedical and Allied Health Sciences, College of Health Sciences, University of Ghana, Accra, Ghana.
3. Department of Medicine, School of Medicine and Dentistry, University of Ghana and Korle-Bu Teaching Hospital, Accra, Ghana.
4. Department of Non-Communicable Disease Epidemiology, London School of Hygiene and Tropical Medicine, London, United Kingdom.
5. Kumasi Centre for Collaborative Research, KNUST, Kumasi, Ghana
6. Julius Global Health, Julius Center for Health Sciences and Primary Care, University Medical Centre, Utrecht University, The Netherlands.
7. Division of Epidemiology & Biostatistics, School of Public Health, Faculty of Health Sciences, University of the Witwatersrand, Johannesburg, South Africa.
8. Institute of Tropical Medicine and International Health, Charité–University Medicine Berlin, Augustenburger Platz 1, 13353 Berlin, Germany.
9. Department of Molecular Epidemiology, German Institute of Human Nutrition Potsdam-Rehbrücke, Nuthetal, Germany.
10. Charité - Universitaetsmedizin Berlin, Institute for Social Medicine, Epidemiology and Health Economics, Berlin, Germany.
11. Department of Endocrinology and Metabolism, Charité-University Medicine Berlin, Berlin, Germany.
12. German Centre for Cardiovascular Research (DZHK), Berlin, Germany.
13. Center for Cardiovascular Research (CCR), Charité-University Medicine Berlin, Berlin, Germany.
14. MKPGMS - Uganda Martyrs University, Kampala, Uganda.
15. Regional Institute for Population Studies, University of Ghana, Legon, Ghana.

For peer review only - http://bmjopen.bmj.com/site/about/guidelines.xhtml

Address correspondence to David Nana Adjei, MSc, PhD. Department of Public Health, Academic Medical Centre, University of Amsterdam, Meibergdreef 9, 1105 AZ Amsterdam, the Netherlands. Department of Medical Laboratory Sciences, School of Biomedical and Allied Health Sciences, University of Ghana, E-mail: dna@chs.edu.gh, d.n.adjei@amc.uva.nl, Tel: +233236717850

For peer review only

Abstract

Objectives: Studies from high income countries suggest higher prevalence of Chronic Kidney Disease (CKD) among individuals in low socio-economic groups. However, some studies from low and middle-income countries (LMICs) show the reverse pattern among those in high socioeconomic groups. It is unknown which pattern applies to individuals living in rural and urban Ghana. We assessed the association between Socio-Economic Status (SES) indicators and CKD in rural and urban Ghana and to what extent the higher SES of people in urban areas of Ghana could account for differences in CKD between rural and urban populations.

Setting: The study was conducted in Ghana (Ashanti region). We used baseline data from a multi-centre Research on Obesity and Diabetes among African Migrants (RODAM) study.

Participants: The sample consisted of 2492 adults (Rural Ghana, 1043, Urban Ghana, 1,449) aged 25 to 70 years living in Ghana.

Exposure: Educational level, occupational level and wealth index.

Outcome: Three CKD outcomes were considered using the 2012 KDIGO (Kidney Disease: Improving Global Outcomes) severity of CKD classification: albuminuria, reduced glomerular filtration rate and high to very high CKD risk based on the combination of these two.

Results: All three SES indicators were not associated with CKD in both rural and urban Ghana after age and sex adjustment except for rural Ghana where high wealth index was significantly associated with higher odds of reduced eGFR (AOR, 2.38; 95% C.I. 1.03-5.47). The higher rate of CKD observed in urban Ghana was not explained by the higher SES of that population.

Conclusion: SES indicators were not associated with prevalence of CKD except for wealth index and reduced eGFR in rural Ghana. Consequently, the higher SES of urban Ghana did not account for the increased rate of CKD among urban dwellers suggesting the need to identify other factors that may be driving this.

Index Words: Chronic kidney disease; socioeconomic status; health inequalities; risk factor; ethnic minority groups; migrants; RODAM study, Ghana

For peer review only - http://bmjopen.bmj.com/site/about/guidelines.xhtml

Strengths and limitation of the study

- The use of well-standardized study protocols across rural and urban Ghana eliminated intra protocol variability.

- Our study is also the first in Africa to use all three categories of CKD definitions (albuminuria, reduced eGFR and CKD risk) by KDIGO 2012 in assessing association of SES with CKD in rural and urban setting. This provides more detailed information on CKD outcomes.

- The limitation of intra laboratory variability in earlier studies was eliminated using the same standard operating procedures in the same laboratory for running all samples for both rural and urban Ghana.

- The use of three constructs of SES (educational level, occupational level and wealth index) in this study also provides a much better holistic approach to assessing SES associations with CKD.

- Our study was limited because of the use of cross sectional design which prevented us from determining causality between predictors and CKD progression.

For peer review only - http://bmjopen.bmj.com/site/about/guidelines.xhtml

Introduction

In general, individuals in lower socio-economic status (SES) groups have been shown to suffer more frequently from Chronic Kidney Disease (CKD), often progressing to End Stage Renal Disease (ESRD), and associated with inadequate dialysis treatment, reduced access to kidney transplantation and poor health outcomes [1]. Recent studies have consistently found low SES to be associated with higher risk of CKD among people of African origin [2-5].

However, in some settings the well-known inverse association between SES and CKD seems to be absent, or even reversed. For example, Bryne et al. did not find any association between SES and End Stage Renal Disease [6]. Other studies have found a positive association between SES and CKD [7 8]. Specifically, as SES improved, unhealthful lifestyle (unhealthy diet, physical inactivity, smoking and alcohol consumption) increased in China while that of the United States decreased with

For peer review only - http://bmjopen.bmj.com/site/about/guidelines.xhtml

improved SES [9]. People with higher incomes, in these contexts, can afford a western lifestyle, which is more readily available in the urban areas than in the rural areas. There is therefore an interaction between individual SES and environmental factors, such as food, alcohol, smoking and sedentary life style in such populations [10-12]. Consequently, in those settings, people with a higher SES might have higher CKD risk.

In urban areas, the population in general has higher SES than in rural areas [13]. For example, individuals with higher educational level migrate from rural areas to find higher occupations matching their higher education to improve on their wealth. If indeed a positive association between SES and CKD is observed in LMICs, this might underlie the well-known health differences between urban and rural areas, with urban areas having an increased risk of CKD [14]. So far, it is unknown whether the reversed SES gradient (higher risk in high SES group) might explain the higher burden of CKD in urban areas as compared to rural areas in Africa.

In view of this, we assessed the association of SES with CKD in rural and urban Ghana and studied the extent to which the higher SES of people in urban areas could account for differences in CKD between rural and urban populations.

Methods

Study population and study design

In the present analyses, data from the RODAM (**R**esearch on **O**besity & **D**iabetes among **A**frican **M**igrants) study, a multi-centre cross-sectional study were used. The

For peer review only - http://bmjopen.bmj.com/site/about/guidelines.xhtml

rationale, conceptual framework, design and methodology of the RODAM study have been described in detail elsewhere [15] [16]. As the Healthy Life in an Urban Setting (HELIUS) study conducted among Ghanaian migrants living in Amsterdam did not find any associations between SES and CKD [17] the current study focused on rural and urban Ghana (Ashanti region of Ghana). The RODAM study was conducted from 2012 to 2015 and it comprised of individuals aged 25-70 years living in rural and urban Ghana and Ghanaian migrants in Europe. All participants below 25 and above 70 years were excluded in the present analyses. The present analysis was restricted to the rural and urban sites (n=2492) RODAM participants. Specifically, 1043 participants from rural Ghana and 1449 from urban Ghana were used in this study.

Data collection for the study was standardized across all sites. Written informed consent was obtained from each participant prior to enrolment in the study. The respective ethics committees in Ghana and the three European countries approved the study protocols before data collection began. Specifically, we obtained ethical clearance in Ghana from School of Medical Sciences/Komfo Anokye Teaching Hospital Committee on Human Research, Publication & Ethical Review Board. In the Netherlands, the Institutional Review Board of the AMC, University of Amsterdam gave approval for the study. In Germany, approval for the study was obtained from the Ethics Committee of Charite-Universitäts medizin. The London School of Hygiene and Tropical Medicine Research Ethics Committee gave approval for the study in the UK.

The response rate was 76% in rural Ghana and 74% in urban Ghana. In Ghana, participants were randomly drawn from a list of 30 enumeration areas in the Ashanti

For peer review only - http://bmjopen.bmj.com/site/about/guidelines.xhtml

region based on the 2010 population census using the multistage random sampling. These enumeration areas came from two purposively selected urban cities (Kumasi and Obuasi) and 15 randomly selected rural communities in the Ashanti region. Selected health and community authorities were first identified, notified of the study and letters were sent giving detailed explanation of the study. We sent team members to stay among the communities to familiarize with them and organize mini clinics in the field. This lasted between 1-2 weeks depending on the sampled population and responsiveness of respondents.

In Ghana, questionnaires administration and physical examination were done at the same day/time. The participants were instructed to fast from 10.00pm the night before the physical examination. For the current study, 2566 participants with data available on both questionnaire data and physical measurements were used. We excluded (n=74) individuals outside the RODAM age range of 25-70 years resulting in a data set of 2492 for analysis. These comprised 1,449 Urban Ghana and 1043 Rural Ghana. For the final analysis, individuals with no data on CKD status (n=42) were excluded.

Measurements

Covariates

Demographic and lifestyle factors

Information on demographics, educational level, occupational level, wealth index and lifestyle factors (smoking and physical activity) were obtained by questionnaire. Physical examinations were performed with validated devices per standardized operational procedures across all study sites. Weight was measured in light clothing and without shoes with SECA 877 scales to the nearest 0.1 kg. Height was measured without shoes with a portable stadiometer (SECA 217) to the nearest 0.1cm. Body mass index (BMI) was calculated as weight (kg) divided by height squared (m^2). Overweight was defined as BMI of

For peer review only - http://bmjopen.bmj.com/site/about/guidelines.xhtml

≥25 to <30 kg/m^2 and obesity as BMI ≥30 kg/m^2 [18]. Per participant, all anthropometrics were measured twice by the same assessor and the average of the two measurements were used for analyses.

Predictor: SES

Socioeconomic indicators used in this study were educational level, occupational status and level of wealth index. Educational level was determined based on self-reported highest educational qualification accomplished based on the Ghanaian educational system. Occupational level was determined based on self-reported current occupation if employed and/or last occupation before retirement or student. The reported occupations were further coded according to the International Standard Classification of Occupations scheme (ISCO-08**).** Where 'high' (professionals, managers, clerical support staff, higher grade routine non-manual employees service and sales-related occupations) and 'low' (craft and related trades workers, elementary occupations and farmers) and the rest were categorized into the 'middle'. Wealth index was determined using the World Health Organization (WHO) standard of wealth index classification. Wealth index was based on data collected in the Household Questionnaire. The questionnaire comprised of questions on household's ownership of several consumer items such as television, car, flooring material, toilet facilities etc. Each household was assigned a standard score for each asset. Wealth index was then expressed in five categories. The five categories were further categorized into three categories by combining the second and third as well as fourth and fifth categories due to small numbers [19]. All three SES constructs were further classified as low, medium and high SES and their relationship to each other tested. A composite SES variable (SES) was generated based on the three SES constructs (education, occupation and wealth index) using the EGEN group command in STATA. The codes were combined into numerical variables and their averages computed. The resultant values were recoded into three categories (low, medium and high).

Co-morbidity factors

Blood pressure (BP) was measured three times using a validated semi-automated device (The Microlife WatchBP home) with appropriate cuffs in a sitting position after at least 5 min rest. The mean of the last two BP measurements was used in the analyses. Hypertension was defined as systolic BP ≥ 140 mmHg, and/or diastolic BP ≥ 90 mmHg, and/or being on antihypertensive medication treatment, and/or self-reported hypertension. Trained research assistants in the two sites collected fasting venous blood samples. All the blood samples were processed and aliquoted immediately (within one hour to maximum three hours of the vena puncture) after collection per standard operation procedures, and then temporarily stored at the local research location at −20°C. The separated samples were then transported to the local research centres laboratories, where they were checked, registered and stored at −80°C. To avoid intra-

For peer review only - http://bmjopen.bmj.com/site/about/guidelines.xhtml

laboratory variability, the stored blood samples from the local research centres were transported to Berlin, Germany for biochemical analyses. Fasting plasma glucose concentration was measured using an enzymatic method (hexokinase). Type 2 diabetes was defined according to the WHO diagnostic criteria (fasting glucose ≥7.0 mmol/L, and/or current use of medication prescribed to treat diabetes, and/or self-reported diabetes) [20]. Concentration of total cholesterol was assessed using colorimetric test kits. All biochemical analyses were performed using an ABX Pentra 400 chemistry analyzer (ABX Pentra; Horiba ABX, Germany). Hypercholesterolemia was defined as total cholesterol level ≥ 6.22 mmol/L. Serum creatinine concentration (in umol/L) was determined by a kinetic colorimetric spectrophotometric isotope dilution mass spectrometry–calibrated method (Roche Diagnostics). Biochemical analyses were subject to extensive quality checks including blinded serial measurements.

Outcome: CKD prevalence

Participants were asked to bring an early morning urine sample for the analyses of albuminuria and creatinine levels. Urinary albumin concentration (in mg/L) was measured by an immunochemical turbidimetric method (Roche Diagnostics). Urinary creatinine concentration (in umol/L) was measured by a kinetic spectrophotometric method (Roche Diagnostics). Estimated glomerular filtration rate (eGFR) was calculated using the CKDEPI (CKD Epidemiology Collaboration) creatinine equation [21]. Urinary albumin-creatinine ratio (ACR; expressed in mg/g) was calculated by taking the ratio between urinary albumin and urinary creatinine. eGFR and albuminuria were categorized according to the 2012 KDIGO (Kidney Disease: Improving Global Outcomes) classification [22]. eGFR was categorized as follows: G1, ≥ 90 mL/min/1.73 m^2 (normal kidney function); G2, 60 to 89 mL/min/1.73 m^2 (mildly decreased); G3a, 45 to 59 mL/min/1.73 m^2 (mildly to moderately decreased); G3b, 30 to 44 mL/min/1.73 m^2 (moderately to severely decreased); G4, 15 to 29 mL/min/1.73 m^2 (severely decreased); and G5, < 15 mL/min/1.73 m^2 (kidney failure). Albuminuria categories were derived from ACR and were as follows: A1, < 3mg/mmol (normal to mildly increased); A2, 3 to 30 mg/mmol (moderately increased); and A3, > 30mg/mmol (severely increased). CKD status was categorized according to severity of kidney disease (green, low risk; yellow, moderately increased risk; orange, high risk; and red, very high risk) using the combination of eGFR (G1-G5) and albuminuria (A1-A3) levels defined by the 2012 KDIGO guideline [23]. Due to the small number of participants in the very high risk category of CKD, high and very high risk groups were combined. Reduced eGFR was defined as eGFR < 60 mL/min/1.73 m^2. Because of the small number of participants in the severely increased albuminuria category, we defined albuminuria as ACR ≥3 mg/mmol by combining the moderately increased (A2) and severely increased (A3) categories.

Patient and Public Involvement

For peer review only - http://bmjopen.bmj.com/site/about/guidelines.xhtml

Community leaders were involved in the recruitment of patients. These comprised of religious communities (churches and mosques), endorsement from local key leaders and establishing relationships with healthcare organizations. We also provided information on the study by involving the local media (radio and television stations). We sent letters to all selected health and community authorities to notify participants of the study. Team members were sent to the various communities to stay among the community and organize mini clinics for a period of 1-2 weeks. Results of the study were disseminated through seminars, durbars and via radio and television stations.

Statistical methods

Participants' characteristics were expressed as absolute numbers and percentages for categorical variables and as means and standard deviations (SD) for continuous variables. CKD prevalence with 5% error bars were presented as bar graphs for each SES construct across rural and urban Ghana. Spearman's rank correlation was used to determine correlations between the three SES constructs. Odds ratios (ORs) and their corresponding 95% confidence intervals (CIs) were estimated by means of logistic regression analyses to study the odds of albuminuria (ACR>3 mg/mmol, A2-A3, moderately to severely increased albuminuria), reduced kidney function (eGFR< 60 mL/min/1.73 m^2, G3-G5 moderately to severely decreased kidney function) and increased CKD risk (high and very high CKD risk) by SES, with adjustments for potential confounders (age and sex).[24] In addition, the analyses were performed for the total population (using low educational level, low occupational status and low level of wealth index as reference categories). Further analysis was conducted to assess the contribution of SES indicators to rural-urban differences in albuminuria, reduced eGFR and CKD risk using rural Ghana as reference. Tolerance test and variance inflation factor (VIF) showed very small degree of collinearity among SES predictors thus we therefore adjusted for each of SES variables separately. Complete case analysis approach was used. All data available were included in the age-adjusted models. All analyses were performed using STATA, version 14.0 (StataCorp LP).

Results

For peer review only - http://bmjopen.bmj.com/site/about/guidelines.xhtml

Table 1 shows characteristics of study participants. Participants in rural Ghana were slightly older than those in urban Ghana. Female preponderance was observed in both rural (61.2%) and urban (71.4%) Ghana, though higher proportions were observed in urban Ghana. Individuals living in rural Ghana were generally less educated (56.9%) compared with those living in urban (43.9%) Ghana. There were slightly more individuals with low occupational status in urban Ghana compared with their peers in rural Ghana. People in urban Ghana (43.4%) were wealthier than their rural (24.9%) counterparts. Rural Ghanaians (47.2%) were more physically active compared with their urban peers. Smoking was low among Ghanaians though rural Ghanaians were more likely to smoke compared with their urban peers. Hypercholesterolemia was more prevalent in urban Ghana than in rural Ghana. Hypertension (36.7%) and type 2 diabetes (10.6%) were more prevalent in urban Ghanaians compared with those living in rural Ghana. Urban Ghanaians were markedly more obese compared with their rural peers. Except for eGFR, albuminuria and CKD risk prevalence rates were higher in urban Ghana compared with rural Ghana.

For peer review only

For peer review only - http://bmjopen.bmj.com/site/about/guidelines.xhtml

Table 1: Baseline characteristics by location

	Rural Ghana	Urban Ghana
Number of participants, N (%)	1043 (41.9)	1449 (58.1)
Mean age, years (SD)	46.5 (12.6)	45.2 (11.4)
Females, N (%)	638 (61.2)	1034 (71.4)
Educational level n (%)		
Low	555 (56.9)	614 (43.9)
Middle	311 (31.9)	547 (39.1)
High	108 (11.2)	239 (17.0)
Occupational status, n (%)		
Low	250 (25.7)	374 (26.7)
Middle	628 (64.5)	818 (58.4)
High	96 (9.8)	209 (14.9)
Wealth index, n (%)		
Low	449 (46.5)	368 (26.6)
Middle	276 (28.6)	416 (30.0)
High	241 (24.9)	602 (43.4)
BMI (kg/m^2		
< 25	794 (76.3)	579 (39.9)
25-29.9	189 (18.2)	495 (34.2)
≥ 30	58 (5.5)	374 (25.9)
Low physical activity, n (%)	663 (47.2)	592 (60.7)

For peer review only - http://bmjopen.bmj.com/site/about/guidelines.xhtml
For peer review only

Smoking, n (%)	22 (2.3)	14 (1.0)
Hypercholesterolemia, n (%)	78 (7.6)	270 (18.7)
Hypertension, n (%)	306 (29.3)	531 (36.7)
	53	
Diabetes, n (%)	(5.1)	153 (10.6)
Albuminuria, n (%)		
	930 (91.6)	
A1, Normal to mildly increased (ACR <3 mg/mmol)	85	1285 (89.1)
A2-A3, moderately to severely increased (ACR≥ 3 mg/	(8.4)	158 (10.9)
eGFR, n (%)		
G1-G2 (≥ 60 mL/min/1.73m^2)	989 (96.3)	1388 (96.3)
G3-G5 (<60 mL/min/1.73m^2)	38 (3.7)	54 (3.7)
CKD risk, n (%)		
Low risk (green)	916 (90.5)	1281 (88.9)
Moderately increased to very high risk (yellow to red)	96 (9.5)	160 (11.1)

Abbreviations: N, number of respondents; SD, standard deviation; eGFR, estimated glomerular filtration rate; ACR, albumin creatinine ratio; CKD, Chronic kidney disease

Figure 1 shows prevalence of CKD by level of education in urban and rural Ghana. Prevalence of CKD decreased with increasing levels of education in rural Ghana. Higher prevalence of CKD was observed among individuals with low educational level compared with those with middle and high educational level. However, those with high educational level in urban Ghana had higher prevalence of CKD compared with those with middle level education. For occupational status, prevalence of CKD was higher among individuals with low occupational status in urban Ghana. Similar patterns were observed in rural Ghana; however, those with higher occupational status had higher prevalence of CKD compared with those with middle occupational status (Figure 2). Figure 3 shows prevalence of CKD by level of wealth index. CKD prevalence among

For peer review only

For peer review only - http://bmjopen.bmj.com/site/about/guidelines.xhtml

the levels of wealth index varied between urban and rural Ghana. Those with middle level wealth index had higher prevalence of CKD compared with those with low or high CKD prevalence in both rural and urban Ghana. CKD prevalence rate for low and high level wealth index in urban Ghana was the same while that of rural Ghana was slightly different.

For peer review only

For peer review only - http://bmjopen.bmj.com/site/about/guidelines.xhtml

Among the whole group, educational level was positively associated with wealth index (p<0.01) and composite SES (P<0.01). Occupational level was also inversely associated with educational level (p<0.01) and wealth index (p<0.01). In urban Ghana, high educational level was positively associated with high wealth index but inversely associated with occupation (p<0.01). In rural Ghana, high education was positively associated with high wealth index (p<0.01), but there was no significant association between education and occupation. High wealth index was inversely associated with high occupational status in both rural and urban Ghana (p<0.01) (Table 2).

Table 2: Relationship between SES constructs (educational, occupational level and wealth index) by urban rural Ghana

Correlation matrix	Educational level	Occupational level	Wealth index	SES
**Whole group**				
Educational level	1.000			
Occupational status	**-0.060**	1.000		
	**0.004**			
Wealth Index	0.282	**-0.121**	1.000	
	0.001	**0.001**		
SES	**1.000**	**-0.059**	**0.282**	1.000
	**0.003**	**0.006**	**0.001**	

For peer review only

For peer review only - http://bmjopen.bmj.com/site/about/guidelines.xhtml

**Urban Ghana**				
Educational level	1.000			
Occupational status	**-0.115**	1.000		
	**0.001**			
Wealth Index	**0.294**	**-0.126**	1.000	
	**0.001**	**0.001**		
SES	**1.000**	**-0.024**	**0.937**	1.000
	**0.002**	**0.001**	**0.001**	
**Rural Ghana**				
Educational level	1.000			
Occupational status	0.017	1.000		
	0.589			
Wealth Index	**0.219**	**-0.135**	1.000	
	**0.001**	**0.001**		
SES	**0.504**	0.017	**0.934**	1.000
	**0.001**	0.587	**0.001**	

For peer review only

For peer review only - http://bmjopen.bmj.com/site/about/guidelines.xhtml

Table 3 shows association between level of education, occupational status, level of wealth index and prevalence of CKD. After adjusting for age and sex for the whole group, albuminuria was associated with middle level education (AOR=0.66, 0.48-0.91, p<0.01). After adjusting for age and sex, we observed no significant association between SES indicators (educational level, occupational status and wealth index) and CKD in urban Ghana. However, middle (AOR=0.51, 0.34-0.76, <0.01) and higher (AOR=0.53, 0.31-0.91, p<0.01) level education was associated with reduced albuminuria in urban Ghana. Whereas educational level and occupational status were not associated with CKD prevalence, high wealth index was significantly associated with higher odds of reduced eGFR in rural Ghana (AOR=2.38, 1.03-5.47, P<0.01).

Table 3: Association of SES indicators (educational level, occupational status and wealth index level) with albuminuria, reduced eGFR and CKD risk

	Albuminuria (ACR ≥ 3 mg/mmol)	eGFR < 60 mL/min/1.73 m2	High to very high CKD risk (KDIGO, 2012)
	OR (95% CI)	OR (95% CI)	OR (95% CI)

For peer review only - http://bmjopen.bmj.com/site/about/guidelines.xhtml

For peer review only

	n (%)	Model 1	n (%)	Model 1	n (%)	Model 1
**Education**						
**Whole group**						
Low	1,152 (11.89)	1.00 (Reference)	1,160 (3.97)	1.00 (Reference)	1.150 (11.91)	1.00 (Reference)
Middle	847 (7.32)	**0.66 (0.48-0.91)**	849 (3.77)	1.36 (0.83-2.22)	845 (8.28)	0.82 (0.59-1.12)
High	343 (7.00)	0.67 (0.42-1.07)	345 (3.19)	1.11 (0.55-2.29)	343 (8.75)	0.96 (0.62-1.49)
**Urban Ghana**						
Low	612 (14.7)	1.00 (Reference)	612 (4.1)	1.00 (Reference)	612 (14.1)	1.00 (Reference)
Middle	546 (7.8)	**0.51 (0.34-0.76)**	546 (3.7)	1.12 (0.59-2.12)	545 (8.1)	**0.59 (0.39-0.89)**
High	238 (8.4)	**0.53 (0.31-0.91)**	238 (3.4)	0.91 (0.37-2.19)	238 (10.9)	0.83 (0.51-1.38)
**Rural Ghana**						
Low	540 (8.7)	1.00 (Reference)	548 (3.8)	1.00 (Reference)	538 (9.5)	1.00 (Reference)
Middle	301 (6.3)	0.89 (0.51-1.59)	303 (3.9)	1.69 (0.77-3.66)	300 (8.7)	1.33 (0.79-2.25)
High	105 (3.8)	0.66 (0.23-1.95)	107 (2.8)	1.28 (0.35-4.71)	105 (3.8)	0.69 (0.23-2.02)
**Occupational status**						
**Whole group**						
Low	614 (9.93)	1.00 (Reference)	616 (2.76)	1.00 (Reference)	613 (9.46)	1.00 (Reference)
Middle	1,427 (9.25)	0.82 (0.59-1.14)	1,436 (3.34)	0.93 (0.52-1.66)	1,424 (9.90)	0.89 (0.65-1.24)
High	302 (10.26)	0.76 (0.47-1.22)	303 (7.92)	1.33 (0.67-2.62)	302 (12.91)	0.90 (0.57-1.42)
**Urban Ghana**						
Low	207 (10.1)	1.00 (Reference)	207 (6.8)	1.00 (Reference)	207 (12.1)	1.00 (Reference)
Middle	817 (11.1)	1.50 (0.88-2.83)	817 (3.6)	1.15 (0.56-2.35)	816 (11.6)	1.37 (0.84-2.56)
High	373 (11.0)	1.57 (0.89-2.53)	373 (2.7)	1.02 (0.41-2.52)	373 (9.7)	1.21 (0.68-2.14)
**Rural Ghana**						
Low	95 (10.5)	1.00 (Reference)	96 (10.4)	1.00 (Reference)	95 (14.7)	1.00 (Reference)
Middle	610 (6.7)	0.65 (0.31-1.37)	619 (3.1)	**0.37 (0.16-0.85)**	608 (7.6)	0.55 (0.28-1.08)
High	241 (8.3)	0.99 (0.43-2.28)	243 (2.9)	0.51 (0.18-1.44)	240 (9.2)	0.94 (0.44-2.01)
**Wealth index**						

For peer review only - http://bmjopen.bmj.com/site/about/guidelines.xhtml

**Whole group**						
Low	808 (9.65)	1.00 (Reference)	813 (3.32)	1.00 (Reference)	808 (9.16)	1.00 (Reference)
Middle	678 (10.91)	1.18 (0.84-1.66)	683 (3.81)	1.30 (0.74-2.28)	675 (12.0)	1.43 (1.02-2.01)
High	835 (8.62)	0.93 (0.66-1.31)	835 (4.19)	1.55 (0.91-2.64)	833 (9.96)	1.21 (0.86-1.69)
**Urban Ghana**						
Low	367 (11.2)	1.00 (Reference)	367 (3.5)	1.00 (Reference)	367 (10.1)	1.00 (Reference)
Middle	414 (12.3)	1.12 (0.73-1.74)	414 (3.9)	1.30 (0.61-2.80)	413 (13.1)	1.45 (0.93-2.27)
High	601 (9.8)	0.82 (0.55-1.25)	600 (3.8)	1.13 (0.55-2.31)	600 (10.8)	1.11 (0.72-1.71)
**Rural Ghana**						
Low	441 (7.9)	1.00 (Reference)	446 (3.1)	1.00 (Reference)	441 (8.4)	1.00 (Reference)
Middle	264 (8.7)	1.13 (0.65-1.98)	269 (3.7)	1.22 (0.52-2.84)	262 (10.3)	1.31 (0.77-2.25)
High	234 (5.6)	0.78 (0.40-1.53)	235 (5.1)	**2.38 (1.03-5.47)**	233 (7.7)	1.16 (0.63-2.14)

Model 1, adjusted for age and sex; Abbreviations: CI, confidence interval; ACR, albumin creatinine ration; eGFR, estimated glomerular filtration rate; CKD, chronic kidney disease; OR, odds ratio, n= total number of individuals in the whole group, rural and urban Ghana among the various levels of SES constructs; %, proportion of individuals with CKD among the various levels of SES constructs in rural and urban Ghana.

Table 4 shows the contribution of all three SES constructs to rural and urban CKD prevalence differences. The odds of albuminuria and CKD risk was significantly higher in urban Ghana compared with rural Ghana ($p<0.01$). The higher rate of CKD observed in urban Ghana was not explained by the higher SES of that population as compared to their rural counterparts.

Table 4: Contribution of SES indicators to rural-urban differences in albuminuria, reduced eGFR and CKD risk

	OR (95% CI)	OR (95% CI)	OR (95% CI)	OR (95% CI)	OR (95% CI)

For peer review only

For peer review only - http://bmjopen.bmj.com/site/about/guidelines.xhtml

	n cases (%)	Model 1	Model 2	Model 3	Model 4	Model 5
**Albuminuria (ACR ≥ 3 mg/mmol**						
Sites	n cases (%)					
Urban Ghana	1,443 (10.9)	**1.37 (1.03-1.81)**	**1.70 (1.25-2.31)**	**1.55 (1.15-2.10)**	**1.62 (1.18-2.19)**	**1.74 (1.27-2.38)**
Rural Ghana	1,015 (8.4)	1.00 (Reference)	1.00 (Reference)	1.00 (Reference)	1.00 (Reference)	1.00 (Reference)
**eGFR < 60 mL/min/1.73 m2**						
**Sites**	n cases (%)					
Urban Ghana	1,442 (3.7)	1.27 (0.82-1.97)	1.20 (0.76-1.89)	1.18 (0.79-1.86)	1.12 (0.70-1.78)	1.07 (0.67-1.72)
Rural Ghana	1,027 (3.7)	1.00 (Reference)	1.00 (Reference)	1.00 (Reference)	1.00 (Reference)	1.00 (Reference)
**High to very high CKD risk**						
**Sites**	n cases (%)					
Urban Ghana	1,441 (11.1)	**1.23 (1.01-1.62)**	**1.44 (1.07-1.93)**	**1.38 (1.03-1.84)**	**1.36 (1.01-1.83)**	**1.40 (1.04-1.91)**
Rural Ghana	1,012 (9.46)	1.00 (Reference)	1.00 (Reference)	1.00 (Reference)	1.00 (Reference)	1.00 (Reference)

454

For peer review only - http://bmjopen.bmj.com/site/about/guidelines.xhtml

Model 1#: adjusted for age and sex; Model 2: adjusted for age, sex and education level; Model 3: adjusted for age, sex and occupational status; Model 4: adjusted for age, sex and wealth index; Model 5: adjusted for age, sex, educational level, occupational status and wealth index; Abbreviations: CI, confidence interval; ACR, albumin creatinine ratio; eGFR, estimated glomerular filtration rate; CKD, chronic kidney disease; OR, odds ratio, n= total number of individuals in rural and urban Ghana; %, proportion of individuals with CKD among urban and rural Ghana.

For peer review only

For peer review only - http://bmjopen.bmj.com/site/about/guidelines.xhtml

Discussion

Key findings

Our study findings show no association between all three SES constructs and the prevalence of CKD in both rural and urban Ghana except for wealth index in rural Ghana, with the risk of CKD being higher in the wealthier populations. The higher rate of CKD observed in urban Ghana could not be attributed to the higher SES of that population compared to their rural counterparts.

Discussion of key findings

Association of SES with CKD in rural and urban Ghana

Our study did not find any significant associations between all three SES constructs and CKD among rural and urban Ghana except for wealth index in rural Ghana. The positive association observed between wealth index in rural Ghana may be due to several reasons. A comparison of the three SES constructs showed higher educational level to be associated with wealth index in both rural and urban Ghana but not occupational level. This seems to suggest that occupational level may not be adequately capturing the SES status of individuals living in these settings in relation to CKD. For example, Masthi et al, compared different SES scales in rural and urban India and concluded that Standard of Living Index (SLI) scale was more accurate for classification of SES in urban and rural settings [25]. Our finding is consistent with other studies, [6 26] which reported no association between SES and CKD in high-income countries and LMICs,

For peer review only - http://bmjopen.bmj.com/site/about/guidelines.xhtml

but in contrast with other studies [2-4] [27] that found positive associations between SES and CKD. The reasons for our current finding are unclear. However, it has been suggested that these inconsistent associations may be due to the varying pathways through which the effect of SES on health status is mediated. For example, at a given educational level marked ethnic differences have been reported. Additionally, similar differences were observed for wealth status at a given income level [28-30].

Contribution of SES to observed CKD risk differences between rural and urban Ghana

We observed higher rates of CKD in urban Ghana compared with rural Ghana, as expected. The observed higher rates of CKD in our study were not explained by the higher SES of that population as compared to their rural counterparts. Our results indicate that this is due to the lack of a clear difference in the SES distribution of rural and urban Ghana observed in this study, as well as to the lack of associations between SES and CKD. Consistent with our findings, in a study conducted in Northern Tanzania SES did not explain increased risk of CKD in urban Tanzania [26]. The lack of associations between SES and CKD could probably and partly be explained by the process of epidemiological transition in relation to the "diffusion theory" of ischemic heart disease mortality. This theory attributes the commencement of ischemic heart disease to individuals in the high SES group due to their ability to afford behaviours (smoking, alcohol and sedentary lifestyles) which increased risk of ischemic heart disease. The lower SES groups were later affected partially because of improved living standards, unhealthy life style imitation and urbanization. The higher SES groups were the first to embrace behavioural changes required to decrease the risk of ischemic heart disease and this resulted in reversing the gradient [31]. The rapid urbanization of some rural communities in the Ashanti region of Ghana and the imitation of urban lifestyle could account for our finding. Also, it could be that whereas the high SES group in urban Ghana has already embraced favourable behavioural changes, those in rural Ghana are yet to do so [32]. This explains the observed association of wealth index with CKD in rural Ghana but not in urban Ghana. Also, the interplay of other less

For peer review only

understood or researched factors (e.g., exposure to nephrotoxins, herbal medications, sepsis, psychosocial factors) may be influencing the pathway in which SES influences CKD prevalence and progression.

Strength and limitation

Our study presents several strengths. First, we used well-standardized study protocols across rural and urban Ghana. Our study is also the first in Africa to use all three categories of CKD definition (albuminuria, reduced eGFR and CKD risk) by KDIGO 2012 in assessing association of SES with CKD in rural and urban setting, this provided more detailed information on CKD outcomes. The limitation of intra laboratory variability in earlier studies was eliminated using the same standard operating procedures in the same laboratory for running all samples for both rural and urban Ghana. The use of three constructs of SES in this study also provides a much better holistic approach to assessing SES. Also, the distribution of SES in our study reflects on the national data allowing for generalization of our findings. Our study was limited by the use of cross sectional design, which prevented us from determining causality between predictors and CKD progression. Furthermore, there were more women than men in our study due to the higher response rate in women compared with men. However, this applied to both rural and urban Ghana. We therefore do not expect this to influence our results in a significant way.

Conclusion

All three SES constructs appear not to be associated with prevalence of CKD in urban and rural Ghana except for wealth index in rural Ghana. The observed higher prevalence of CKD in urban Ghana was not explained by the higher SES in urban Ghana. Our study seems to suggest that other non-traditional factors such as nephrotoxins, herbal medications, psychosocial stressors and misuse of over the counter drugs may play a role and underscores the need to further explore these factors.

For peer review only - http://bmjopen.bmj.com/site/about/guidelines.xhtml

Acknowledgement

The authors are very grateful to the research assistants, interviewers and other staff of the five research locations who took part in gathering the data and the Ghanaian volunteers in all the participating RODAM sites. We gratefully acknowledge the advisory board members for their valuable support in shaping the RODAM study methods and the Academic Medical Centre Biobank for their support in biobank management and high-quality storage of collected samples.

Contributors

My co-authors have all contributed substantially to this manuscript and approve of this submission. Research idea and study design: DNA, CA, KS, DA, EB, KM, JA; data acquisition and curation: DNA, CA, EB, KM, data analysis/interpretation: DNA, CA, KS, DA, EB, KM, LS, JA, EOD, KKG, FPM, ID, JS, SB, ADA; statistical analysis: DNA, CA, KS. DNA, CA, KS, DA, EB, KM, LS, JA, EOD, KKG, FPM, ID, JS, SB, ADA contributed important intellectual content during manuscript drafting or revision and accepts accountability for the overall work by ensuring that questions pertaining to the accuracy or integrity of any portion of the work are appropriately investigated and resolved. DNA and CA take responsibility that this study has been reported honestly, accurately, and transparently; that no important aspects of the study have been omitted; and that any discrepancies from the study as planned have been explained.

Funding

This work was supported by the European Commission under the Framework Programme (Grant Number: 278901). The funders had no role in study design, data collection and analysis, decision to publish, or preparation of the manuscript. The Wellcome Trust supported Professor Smeeth's contribution, grant number WT082178. Professor Joachim Spranger was supported by the DZHK (German Center for cardiovascular research) and the Berlin Institute of Health (BIH).

Competing interest: I have communicated with all my co-authors and obtained their full disclosures. My co-authors and I declare no conflicts of interest.

For peer review only

Patient Consent: None declared

Ethics approval: IRBs at each participating site.

Data sharing statement: Data are available from the RODAM research cohort, a third party. Dr. Eric Beune affiliated with the RODAM research cohort and a co-author of this paper in accordance with the RODAM requirements for collaboration. Dr. Beune is the Data Collection Coordinator of RODAM and may be contacted with further questions (e.j.beune@amc.uva.nl). Additionally, researchers interested in further collaboration with RODAM may see the following URL: http://www.rod-am.eu/

For peer review only

References

1. Patzer RE, McClellan WM. Influence of race, ethnicity and socioeconomic status on kidney disease. *Nature Reviews Nephrology* 2012;8(9):533-41.
2. Merkin SS, Roux AVD, Coresh J, et al. Individual and neighborhood socioeconomic status and progressive chronic kidney disease in an elderly population: The Cardiovascular Health Study. *Soc Sci Med* 2007;65(4):809-21.
3. Bello AK, Peters J, Rigby J, et al. Socioeconomic status and chronic kidney disease at presentation to a renal service in the United Kingdom. *Clin J Am Soc Nephrol* 2008;3(5):1316-23.
4. Crews DC, Charles RF, Evans MK, et al. Poverty, race, and CKD in a racially and socioeconomically diverse urban population. *Am J Kidney Dis* 2010;55(6):992-1000.
5. Shoham DA, Vupputuri S, Roux AVD, et al. Kidney disease in life-course socioeconomic context: the Atherosclerosis Risk in Communities (ARIC) Study. *Am J Kidney Dis* 2007;49(2):217-26.
6. Byrne C, Nedelman J, Luke RG. Race, socioeconomic status, and the development of end-stage renal disease. *Am J Kidney Dis* 1994;23(1):16-22.
7. Popkin BM. The shift in stages of the nutrition transition in the developing world differs from past experiences! *Public Health Nutr* 2002;5(1A):205-14.
8. Du S, Lu B, Zhai F, et al. A new stage of the nutrition transition in China. *Public Health Nutr* 2002;5(1a):169-74.
9. Kim S, Symons M, Popkin BM. Contrasting socioeconomic profiles related to healthier lifestyles in China and the United States. *Am J Epidemiol* 2004;159(2):184-91.
10. Adler NE, Ostrove JM. Socioeconomic status and health: what we know and what we don't. *Ann N Y Acad Sci* 1999;896(1):3-15.
11. Anderson NB, Armstead CA. Toward understanding the association of socioeconomic status and health: A new challenge for the biopsychosocial approach. *Psychosom Med* 1995;57(3):213-25.

For peer review only

For peer review only - http://bmjopen.bmj.com/site/about/guidelines.xhtml

12. Feinstein JS. The relationship between socioeconomic status and health: a review of the literature. *The Milbank Quarterly* 1993:279-322.
13. Samuel P, Antonisamy B, Raghupathy P, et al. Socio-economic status and cardiovascular risk factors in rural and urban areas of Vellore, Tamilnadu, South India. *Int J Epidemiol* 2012;41(5):1315-27.
14. Christie S, Fone DL. Does car ownership reflect socio-economic disadvantage in rural areas? A cross-sectional geographical study in Wales, UK. *Public Health* 2003;117(2):112-16.
15. Agyemang C, Beune E, Meeks K, et al. Rationale and cross-sectional study design of the Research on Obesity and type 2 Diabetes among African Migrants: the RODAM study. *BMJ open* 2015;4(3):e004877.
16. Addo J, Agyemang C, Aikins Ad-G, et al. Association between socioeconomic position and the prevalence of type 2 diabetes in Ghanaians in different geographic locations: the RODAM study. *J Epidemiol Community Health* 2017:jech-2016-208322.
17. Adjei DN, Stronks K, Adu D, et al. Relationship between educational and occupational levels, and Chronic Kidney Disease in a multi-ethnic sample-The HELIUS study. *PLoS One* 2017;12(11):e0186460.
18. Consultation WE. Waist circumference and waist-hip ratio. *Report of a WHO Expert Consultation Geneva: World Health Organization* 2008:8-11.
19. Rutstein SO, Johnson K, MEASURE OM. The DHS wealth index: ORC Macro, MEASURE DHS 2004.
20. Association AD. Standards of medical care in diabetes—2014. *Diabetes Care* 2014;37(Supplement 1):S14-S80.
21. Stevens LA, Schmid CH, Greene T, et al. Comparative performance of the CKD epidemiology collaboration (CKD-EPI) and the modification of diet in renal disease (MDRD) study equations for estimating GFR levels above 60 mL/min/1.73 m 2. *Am J Kidney Dis* 2010;56(3):486-95.
22. Eknoyan G, Lameire N, Eckardt K, et al. KDIGO 2012 clinical practice guideline for the evaluation and management of chronic kidney disease. *Kidney Int* 2013;3:5-14.
23. KDIGO G. Work Group. KDIGO clinical practice guideline for glomerulonephritis. *Kidney inter, Suppl* 2012;2:139-274.
24. Collins AJ, Foley RN, Herzog C, et al. US Renal Data System 2010 Annual Data Report. *American journal of kidney diseases: the official journal of the National Kidney Foundation* 2011;57(1 Suppl 1):A8, e1.
25. Masthi NR, Gangaboraiah PK. An exploratory study on socio economic status scales in a rural and urban setting. *Journal of family medicine and primary care* 2013;2(1):69.
26. Stanifer JW, Maro V, Egger J, et al. The epidemiology of chronic kidney disease in Northern Tanzania: a population-based survey. *PLoS One* 2015;10(4):e0124506.
27. Bruce MA, Beech BM, Crook ED, et al. Association of socioeconomic status and CKD among African Americans: the Jackson Heart Study. *Am J Kidney Dis* 2010;55(6):1001-08.
28. Braveman PA, Cubbin C, Egerter S, et al. Socioeconomic status in health research: one size does not fit all. *JAMA* 2005;294(22):2879-88.
29. Gilthorpe MS, Wilson RC. Rural/urban differences in the association between deprivation and healthcare utilisation. *Soc Sci Med* 2003;57(11):2055-63.

For peer review only

30. Choi AI, Weekley CC, Chen S-C, et al. Association of educational attainment with chronic disease and mortality: the Kidney Early Evaluation Program (KEEP). *Am J Kidney Dis* 2011;58(2):228-34.
31. Mackenbach JP, Cavelaars A, Kunst AE, et al. Socioeconomic inequalities in cardiovascular disease mortality. An international study. *Eur Heart J* 2000;21(14):1141-51.
32. McKay L, Macintyre S, Ellaway A. Migration and health: a review of the international literature. MRC Social and Public Health Sciences Unit, Occasional Paper No. 12. *Glasgow: Medical Research Council[Links]* 2003

Legend for figures

Figure 1: Prevalence of chronic kidney disease (CKD) across level of education among urban and rural participants. Definitions according to 2012 KDIGO (Kidney Disease: Improving Global Outcomes) guideline. CKD was defined as being in moderately increased risk, high-risk, or very high-risk groups.

Figure 2: Prevalence of chronic kidney disease (CKD) across occupational status among urban and rural participants. Definitions according to 2012 KDIGO (Kidney Disease: Improving Global Outcomes) guideline. CKD was defined as being in moderately increased risk, high-risk, or very high-risk groups.

Figure 3: Prevalence of chronic kidney disease (CKD) across wealth index categories among urban and rural participants. Definitions according to 2012 KDIGO (Kidney Disease: Improving Global Outcomes) guideline. CKD was defined as being in moderately increased risk, high-risk, or very high-risk groups.

For peer review only

For peer review only - http://bmjopen.bmj.com/site/about/guidelines.xhtml

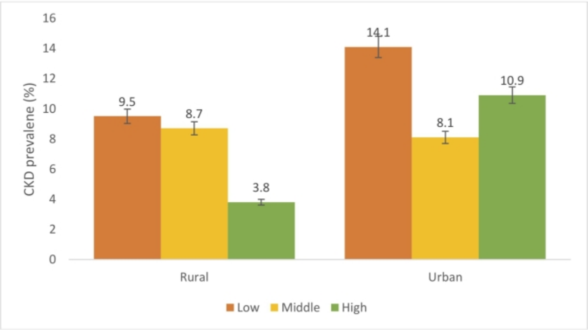

Figure 1: Prevalence of chronic kidney disease (CKD) across level of education among urban and rural participants Definitions according to 2012 KDIGO (Kidney Disease: Improving Global Outcomes) guideline. CKD was defined as being in moderately increased risk, high-risk, or very high-risk groups

Figure 1: Prevalence of chronic kidney disease (CKD) across level of education among urban and rural participants Definitions according to 2012 KDIGO (Kidney Disease: Improving Global Outcomes) guideline. CKD was defined as being in moderately increased risk, high-risk, or very high-risk groups.

279x361mm (300 x 300 DPI)

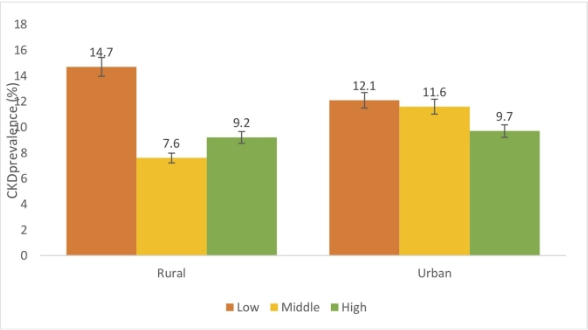

Figure 2: Prevalence of chronic kidney disease (CKD) across occupational status among urban and rural participants. Definitions according to 2012 KDIGO (Kidney Disease: Improving Global Outcomes) guideline. CKD was defined as being in moderately increased risk, high-risk, or very high-risk groups

Figure 2: Prevalence of chronic kidney disease (CKD) across occupational status among urban and rural participants. Definitions according to 2012 KDIGO (Kidney Disease: Improving Global Outcomes) guideline. CKD was defined as being in moderately increased risk, high-risk, or very high-risk groups.

279x361mm (300 x 300 DPI)

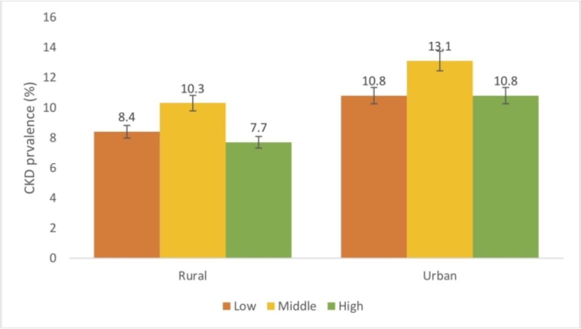

Figure 3: Prevalence of chronic kidney disease (CKD) across wealth index categories among urban and rural participants. Definitions according to 2012 KDIGO (Kidney Disease: Improving Global Outcomes) guideline. CKD was defined as being in moderately increased risk, high-risk, or very high-risk groups

Figure 3: Prevalence of chronic kidney disease (CKD) across wealth index categories among urban and rural participants. Definitions according to 2012 KDIGO (Kidney Disease: Improving Global Outcomes) guideline. CKD was defined as being in moderately increased risk, high-risk, or very high-risk groups.

279x361mm (300 x 300 DPI)

STROBE Statement—checklist of items that should be included in reports of observational studies

	Item No.	Recommendation	Page No.	Relevant text from manuscript
**Title and abstract**	1	(*a*) Indicate the study's design with a commonly used term in the title or the abstract	1,2	We have included a commonly used term in the title and abstract
		(*b*) Provide in the abstract an informative and balanced summary of what was done and what was found	2	Our study did not find any associations between SES indicators and CKD in both rural and urban Ghana after age and sex adjustment except in rural Ghana where wealth index was associated with prevalence of CKD. Consequently, the higher SES did not account for the increased rate of CKD among urban dwellers suggesting the need to identify other factors that may be driving this.
**Introduction**				
Background/rationale	2	Explain the scientific background and rationale for the investigation being reported	4	The theoretical and scientific background as well as the rationale for conducting the study have been provided in the introduction section.
Objectives	3	State specific objectives, including any prespecified hypotheses	4	We assessed the association of SES with CKD in rural and urban Ghana and studied what extent the higher SES of people in urban areas could account for differences in CKD between rural and urban populations
**Methods**				
Study design	4	Present key elements of study design early in the paper	5-6	Details given in the methods
Setting	5	Describe the setting, locations, and relevant dates, including periods of recruitment, exposure, follow-up, and data collection	5-6	Rural or urban Ghana.

For peer review only - http://bmjopen.bmj.com/site/about/guidelines.xhtml

Participants	6	(*a*) *Cohort study*—Give the eligibility criteria, and the sources and methods of selection of participants. Describe methods of follow-up<br>*Case-control study*—Give the eligibility criteria, and the sources and methods of case ascertainment and control selection. Give the rationale for the choice of cases and controls<br>*Cross-sectional study*—Give the eligibility criteria, and the sources and methods of selection of participants	6-8	A multi-centre cross-sectional study was conducted among Ghanaian adults (n=2492) aged 25-70 years residing in rural and urban Ghana.
		(*b*) *Cohort study*—For matched studies, give matching criteria and number of exposed and unexposed<br>*Case-control study*—For matched studies, give matching criteria and the number of controls per case		
Variables	7	Clearly define all outcomes, exposures, predictors, potential confounders, and effect modifiers. Give diagnostic criteria, if applicable	6-8	The main outcomes have been clearly defined.
Data sources/ measurement	8*	For each variable of interest, give sources of data and details of methods of assessment (measurement). Describe comparability of assessment methods if there is more than one group	*6-8*	We defined each variable of interest in the methods accordingly
Bias	9	Describe any efforts to address potential sources of bias	18	Potential sources of bias have discussed in the discussion section
Study size	10	Explain how the study size was arrived at	5	Given in the methods section and we have also referred to the RODAM study methods paper

Continued on next page

For peer review only - http://bmjopen.bmj.com/site/about/guidelines.xhtml

Quantitative variables	11	Explain how quantitative variables were handled in the analyses. If applicable, describe which groupings were chosen and why	8-9	Please see methods
Statistical methods	12	(*a*) Describe all statistical methods, including those used to control for confounding	8-9	Please see methods
		(*b*) Describe any methods used to examine subgroups and interactions	8-9	Please see methods
		(*c*) Explain how missing data were addressed	8-9	
		(*d*) *Cohort study*—If applicable, explain how loss to follow-up was addressed<br>*Case-control study*—If applicable, explain how matching of cases and controls was addressed<br>*Cross-sectional study*—If applicable, describe analytical methods taking account of sampling strategy	NA	We have reported non-response across sites
		(*e*) Describe any sensitivity analyses	NA	
**Results**				
Participants	13*	(a) Report numbers of individuals at each stage of study—eg numbers potentially eligible, examined for eligibility, confirmed eligible, included in the study, completing follow-up, and analysed	5	Non-response analysis was done to shed light on the differential response rates across sites
		(b) Give reasons for non-participation at each stage	5	
		(c) Consider use of a flow diagram	5	We have also referred to RODAM methods paper
Descriptive data	14*	(a) Give characteristics of study participants (eg demographic, clinical, social) and information on exposures and potential confounders	5	We have also referred to RODAM methods paper
		(b) Indicate number of participants with missing data for each variable of interest	5	We have also referred to RODAM methods paper
		(c) *Cohort study*—Summarise follow-up time (eg, average and total amount)	NA	
Outcome data	15*	*Cohort study*—Report numbers of outcome events or summary measures over time	*NA*	
		Case-control study—Report numbers in each exposure category, or summary measures of exposure	*NA*	
		Cross-sectional study—Report numbers of outcome events or summary measures	*9-10*	Summary measures are given in the results section and in tables and figures
Main results	16	(*a*) Give unadjusted estimates and, if applicable, confounder-adjusted estimates and their precision (eg, 95% confidence interval). Make clear which confounders were adjusted for and why they were included	*12-16*	Unadjusted and adjusted estimates are given in the results section and in figures
		(*b*) Report category boundaries when continuous variables were categorized	12-16	We have provided mean and corresponding standard deviations for the continuous variables.

For peer review only - http://bmjopen.bmj.com/site/about/guidelines.xhtml

		(*c*) If relevant, consider translating estimates of relative risk into absolute risk for a meaningful time	NA	

Continued on next page

Other analyses	17	Report other analyses done—eg analyses of subgroups and interactions, and sensitivity analyses	NA	
**Discussion**				
Key results	18	Summarise key results with reference to study objectives	8	
Limitations	19	Discuss limitations of the study, taking into account sources of potential bias or imprecision. Discuss both direction and magnitude of any potential bias	18	Key limitations regarding study methods including differential response rates and sampling methods in the various study sites have been provided
Interpretation	20	Give a cautious overall interpretation of results considering objectives, limitations, multiplicity of analyses, results from similar studies, and other relevant evidence	17-18	Cautious overall interpretation of the key findings have been provided.
Generalisability	21	Discuss the generalisability (external validity) of the study results	17-18	
**Other information**				
Funding	22	Give the source of funding and the role of the funders for the present study and, if applicable, for the original study on which the present article is based	19	The funders had no role in study design, data collection and analysis, decision to publish, or preparation of the manuscript

*Give information separately for cases and controls in case-control studies and, if applicable, for exposed and unexposed groups in cohort and cross-sectional studies.

Note: An Explanation and Elaboration article discusses each checklist item and gives methodological background and published examples of transparent reporting. The STROBE checklist is best used in conjunction with this article (freely available on the Web sites of PLoS Medicine at http://www.plosmedicine.org/, Annals of Internal Medicine at http://www.annals.org/, and Epidemiology at http://www.epidem.com/). Information on the STROBE Initiative is available at www.strobe-statement.org.

For peer review only - http://bmjopen.bmj.com/site/about/guidelines.xhtml
